# Effects of modified Soyo-san (Xiao-yao-san, Shoyo-san) combined with antidepressants on post-stroke depression and functional recovery: a systematic review and meta-analysis

**DOI:** 10.3389/fphar.2025.1651831

**Published:** 2026-01-02

**Authors:** Jeongrim Bak, Hyowon Jin, Jong-Min Yun, Jungtae Leem

**Affiliations:** 1 Department of Korean Internal Medicine, College of Korean Medicine, Wonkwang University, Iksan, Republic of Korea; 2 Research Center of Traditional Korean Medicine, College of Korean Medicine, Wonkwang University, Iksan, Republic of Korea; 3 Department of Diagnostics, College of Korean Medicine, Wonkwang University, Iksan, Republic of Korea; 4 Department of Il-won Integrated Medicine, Wonkwang University Korean Medicine Hospital, Iksan-si, Republic of Korea

**Keywords:** stroke, post-stroke depression (PSD), herbal medicine (HM), systematic review, soyo-san

## Abstract

**Background:**

Over one-third of stroke survivors experience Post Stroke Depression. Conventional antidepressants are effective but have adverse effects. Soyo-san is an herbal medicine used to treat neuropsychiatric diseases that may exert antidepressant effects with fewer adverse effects. However, there is insufficient evidence synthesizing existing Randomized Controlled Trials to provide comprehensive guidance on the effectiveness and safety of combination treatment with Soyo-san.

**Purpose:**

We evaluated the additional benefits and safety of combining Soyo-san with conventional antidepressants for treating Post Stroke Depression through a systematic review and meta-analysis.

**Methods:**

A comprehensive search of seven databases was conducted on October 10, 2024, followed by study selection and data extraction. Methodological quality was assessed using the Cochrane Collaboration’s risk-of-bias tool, and evidence quality was evaluated using the Grading of Recommendations Assessment, Development, and Evaluation method. Quantitative data synthesis and meta-analysis were conducted using RStudio, along with subgroup, sensitivity, and publication bias analyses.

**Results:**

The search retrieved 41 RCTs with 3,628 participants. Soyo-san had a significant effect on depression based on Hamilton Depression Scale score (MD: −4.01; 95% CI: −4.72, −3.30; *I* ^2^ = 94%) and total effective rate (RR: 1.21; 95% CI: 1.17, 1.25; *I* ^2^ = 0%) with any antidepressant. Moreover, Soyo-san improved post-stroke recovery of motor, cognitive, and sleep dysfunctions. Adverse events were reported in both treatment and control groups in most studies but were less frequent in the former.

**Conclusion:**

Modified Soyo-san combined with antidepressants was associated with improvements in depressive symptoms and variety of functional outcomes, though the certainty of evidence is low to very low. Therefore, these findings should be interpreted with caution, and high-quality international trials are needed before firm conclusions can be drawn.

**Systematic Review Registration:**

https://www.crd.york.ac.uk/PROSPERO/view/CRD42024510361, identifier CRD42024510361.

## 1 Introduction

Stroke is a neurological disorder caused by cerebrovascular occlusion, stenosis, or damage, which leads to impaired brain function. The prevalence, incidence, and mortality rates of stroke have steadily increased, imposing a significant socioeconomic burden. According to the Global Burden of Disease (2019), lower-income countries have significantly higher stroke mortality and disability rates than high-income countries (eClinicalMedicine, 2023). Stroke survivors frequently experience various complications and sequelae, and over one-third of stroke survivors experience post-stroke depression (PSD), increasing suicide risk and mortality rates. PSD is also associated with functional deterioration (Robinson and Jorge, 2016).

The Diagnostic and Statistical Manual of Mental Disorders, Fifth Edition (DSM-5) defines PSD as a mood disorder characterized by depressive features, major depression-like episodes, or a combination of mood changes that occur after the onset of stroke. Vascular depression has unique characteristics that distinguish it from PSD; however, they share commonalities and are highly correlated (Robinson and Jorge, 2016). Current treatments for PSD primarily involve antidepressants such as selective serotonin reuptake inhibitors (SSRIs), but no single class of antidepressant has been proven to be significantly more effective than others. The American Heart Association/American Stroke Association guidelines emphasize the need for further research on the most effective medications for PSD treatment (Towfighi et al., 2017). SSRIs are a commonly used class of antidepressants with various adverse effects. They are associated with vascular complications and increased risk of falls in elderly patients, and epidemiological studies have reported associations with increased mortality in stroke, myocardial infarction, and all-cause mortality (Robinson and Jorge, 2016; Starkstein and Hayhow, 2019). Notably, individuals treated with SSRIs have 51% increased risk of intracranial hemorrhage and 42% increased risk of intracerebral hemorrhage (Hackam and Mrkobrada, 2012; Kubiszewski et al., 2021).

As an alternative or complementary approach, non-pharmacological therapies, such as psychosocial interventions and neuromodulation techniques, are available. Cognitive behavioral therapy and repetitive transcranial magnetic stimulation are helpful, but whether they can substitute for pharmacotherapy remains inconclusive, and opinions on their effectiveness vary (Guo et al., 2022). Therefore, exploring alternatives to current PSD treatments and finding therapies to enhance antidepressant effectiveness while reducing adverse effects is necessary.

Herbal medicine (HM) has traditionally been used in East Asia for the treatment of neuropsychiatric diseases and is still used as a primary or alternative treatment for major depressive disorder, dementia, and other neuropsychiatric disorders (Kwon and Lee, 2021; Lee et al., 2022; Kim et al., 2023; Seung et al., 2023). Soyo-san (SYS), also known as Xiao-yao-san or Shoyo-san, is a traditional herbal formula first described in the 12th-century Chinese medical text, ‘Prescriptions from the Great Peace Imperial Grace Pharmacy (太平惠民和劑局方)’. It is composed of eight medicinal herbs: *Bupleurum chinense* DC. [Apiaceae; Bupleuri Radix]*, Paeonia lactiflora* Pall. [Paeoniaceae; Paeoniae Radix], *Angelica sinensis* (Oliv.) Diels [Apiaceae; Angelicae Sinensis Radix], *Atractylodes macrocephala* Koidz. [Asteraceae; Atractylodis Macrocephalae Rhizoma], *Wolfiporia cocos* (Schw.) Ryvarden and Gilb. [Polyporaceae; Poria] (Fungus)*, Glycyrrhiza uralensis* Fisch. [Fabaceae; Glycyrrhizae Radix et Rhizoma], *Mentha arvensis* L [Lamiaceae; Menthae Haplocalycis Herba], and *Zingiber officinale Roscoe* [Zingiberaceae; Zingiberis Rhizoma Recens] (Lee and Jeong, 2017). Preclinical studies have provided evidence for its pharmacological mechanisms; SYS has been shown to exert anxiolytic and neuroprotective effects by modulating α-synuclein and corticosterone levels in the hippocampus (Cao et al., 2016), regulate iron-dependent apoptosis by promoting GPX4 expression (Jiao et al., 2021), and activate the PI3K/Akt pathway, suggesting potential in ischemic stroke treatment (Xu et al., 2021). The specific pharmacological properties of each constituent herb are detailed in Table 1. In Korea, traditional medicine clinical practice guidelines recommend the use of Soyo-san for PSD, as an alternative or complementary treatment to conventional antidepressants (Evidence Based Korean Medicine Clinical Practice Guideline Development Committee for Depression (Evidence Based Korean Medicine Clinical Practice Guideline Development Committee for Depression, 2016).

**TABLE 1:** Pharmacological properties of constituent medicinal plants in Soyo-san.

Scientific name	Parts used	Chinese name	Pharmacological effects
*Bupleurum chinense* DC. [Apiaceae; Bupleuri Radix]	Dried root	Chaihu (柴胡)	Antidepressant properties (Kwon et al., 2010; Zhang et al., 2020)
*Paeonia lactiflora* Pall. [Paeoniaceae; Paeoniae Radix]	Dried root	Baishao (白芍)	Antidepressant properties (Zhang et al., 2020)
*Angelica sinensis (Oliv.)* Diels [Apiaceae; Angelicae Sinensis Radix]	Dried root	Danggui (当归)	Antidepressant properties (Gong et al., 2019)
*Wolfiporia cocos* (Schw.) Ryvarden and Gilb. [Polyporaceae; Poria] (Fungus)	Dried sclerotium	Fuling (茯苓)	Antidepressant properties (Huang et al., 2020)
*Atractylodes macrocephala* Koidz. [Asteraceae; Atractylodis Macrocephalae Rhizoma]	Dried rhizome	Baizhu (白朮)	Antioxidant and anti-inflammatory (Hoang et al., 2016)
*Glycyrrhiza uralensis* Fisch. [Fabaceae; Glycyrrhizae Radix et Rhizoma]	Dried root and rhizome	Gancao (甘草)	Neuroprotective effects (Kao et al., 2014)
*Mentha arvensis* L. [Lamiaceae; Menthae Haplocalycis Herba]	Dried herb	Bohe (薄荷)	Antioxidant and anti-inflammatory (Koşar et al., 2004)
*Zingiber officinale* Roscoe [Zingiberaceae; Zingiberis Rhizoma Recens]	Raw root	Shengjiang (生薑)	Antioxidant and anti-inflammatory (Van Breemen et al., 2011)

Several systematic reviews have compared the effectiveness of Soyo-san with antidepressants in treating PSD. However, most studies have combined randomized controlled trials (RCTs) administering Soyo-san and antidepressants separately (head-to-head), along with RCTs administering Soyo-san in combination with antidepressants for comparison with antidepressants alone (Zhang et al., 2012; Lai, 2018; Zhao J. et al., 2020b; Wang et al., 2022). Few studies were outdated (Zhang et al., 2012) or had limitations of inadequate assessment of the quality of evidence, such as the Grading of Recommendations Assessment, Development and Evaluation (GRADE) methodology (Jin et al., 2018; Lai, 2018; Zhao J. et al., 2020b; Wang et al., 2022), or lacked exploration of factors influencing the study effects (e.g., publication year, treatment length) through meta-regression analysis. Furthermore, the scales employed in earlier studies were limited to a maximum of seven, leading to potential confusion and the possibility of fragmented results (Zhang et al., 2012; Jin et al., 2018; Lai, 2018; Zhao J. et al., 2020b; Wang et al., 2022).

A quantitative synthesis and quality evaluation was conducted through a systematic review to assess the effectiveness and safety of Soyo-san when used in combination with antidepressants for PSD treatment compared to antidepressants alone. The GRADE methodology was applied to assess the quality of evidence. Subgroup analyses, along with meta-regression analyses, were conducted to identify the factors influencing the effectiveness of Soyo-san administration for PSD, with the aim of providing insights for further research and clinical applications.

## 2 Materials and methods

The protocols for the data and methods used in our study were preregistered with Open Science Framework and PROSPERO (CRD42024510361). We anticipated between-study differences and therefore used a random-effects model. Between-study variability was assessed with I^2^ and τ^2^. We also conducted sensitivity analyses, including leave-one-out and influence diagnostics, to examine the robustness of the findings. When heterogeneity was substantial (I^2^>75%), we explored potential sources through subgroup and meta-regression analyses.

### 2.1 Data sources and search strategy

Two researchers (JRB, HWJ) independently conducted comprehensive searches of four English databases: MEDLINE (PubMed), EMBASE (Elsevier), CENTRAL (the Cochrane Central Register of Controlled Trials), and the Cumulative Index to Nursing and Allied Health Literature, via EBSCO. Additionally, searches were performed on three non-English databases: CNKI (Chinese National Knowledge Infrastructure Database) in Chinese, CiNii (Citation Information by NII) in Japanese, and KCI (Korea Citation Index) in Korean. The initial search was conducted on February 14, 2023, and updated on October 10, 2024, to include the most recent information and relevant evidence. Relevant studies were identified by reviewing the reference lists of previous studies (Zhang et al., 2012; Jin et al., 2018; Lai, 2018; Zhao J. et al., 2020b; Wang et al., 2022). No restrictions on language, publication date, or publication status were applied. For MEDLINE, the following search strategy was used: (“depressive disorder” [MeSH Terms] OR “depression” [MeSH Terms] OR depressive OR depression) AND (“stroke” [MeSH Terms] OR stroke) AND (jia-wei-xiao-yao-san OR xiao-yao-san OR xiao-yao powder OR xiao-yao wan OR kamishoyosan OR TJ-24 OR Soyosan OR Soyo-san OR Gami-soyosan OR Gamisoyo-san) (Supplementary Table S1).

### 2.2 Inclusion criteria

#### 2.2.1 Types of studies

RCTs involving human participants were included. RCTs that mentioned “randomized (随机)” without specified description of randomization methods were included. Studies using quasi-randomization methods such as allocation by order of admission, alternate allocation, or date of birth were excluded. Both parallel and crossover designs were included. *In vivo* and *in vitro* studies, case reports, retrospective studies, and non-RCTs were excluded.

#### 2.2.2 Participant characteristics

Studies involving patients diagnosed with PSD using standardized diagnostic tools, irrespective of sex, age, or race, were included.

#### 2.2.3 Intervention types

Studies with administration of Soyo-san along with conventional pharmacological therapy in treatment group, composed of eight herbs including *Bupleurum chinense* DC. [Apiaceae; Bupleuri Radix]*, Paeonia lactiflora* Pall. [Paeoniaceae; Paeoniae Radix], *Angelica sinensis* (Oliv.) Diels [Apiaceae; Angelicae Sinensis Radix], *Atractylodes macrocephala* Koidz. [Asteraceae; Atractylodis Macrocephalae Rhizoma], *Wolfiporia cocos* (Schw.) Ryvarden and Gilb. [Polyporaceae; Poria] (Fungus)*, Glycyrrhiza uralensis* Fisch. [Fabaceae; Glycyrrhizae Radix et Rhizoma], *Mentha arvensis* L [Lamiaceae; Menthae Haplocalycis Herba], and *Zingiber officinale* Roscoe [Zingiberaceae; Zingiberis Rhizoma Recens] were adopted (Table 1). Given that HMs are often modified to enhance their effectiveness by adjusting their composition, this study considered modified versions as Soyo-san if they contained 50% or more of the standard composition. Only oral administration of Soyo-san was considered, and all preparations were allowed. Studies that combined Soyo-san with other treatments were included only if the same additional treatment was used in both the intervention and control groups. Studies in which HMs other than Soyo-san were used in the control group were excluded to assess the specific effects of Soyo-san. In the control group, only pharmacological therapy was administered.

#### 2.2.4 Outcome measures

The Hamilton Depression Scale (HAMD), designed to assess depression levels, was selected as the primary outcome measure (Bobo et al., 2016). Secondary outcome measures included: (1) level of serotonin (5-hydroxytryptamine [5-HT]), a neurotransmitter known to be associated with depression; (2) National Institutes of Health Stroke Scale (NIHSS), which evaluates the level of severity, rehabilitation, and clinical symptoms of stroke (Farooque et al., 2020), (3) Scandinavian Stroke Scale (SSS), same as NIHSS(Barber et al., 2004); (4) Fugl-Meyer Assessment Scale (FMA), a post-stroke motor function impairment assessment (Gladstone et al., 2002); (5) Modified Barthel Index (MBI), a scale to evaluate independence in activities of daily living in stroke patients (Quinn et al., 2011); (6) Mini-Mental State Examination (MMSE), a tool to evaluate post-stroke cognitive levels and the potential for dementia development (Nys et al., 2005). Considering the influence of sleep health on post-stroke cognitive impairment, (7) Pittsburgh Sleep Quality Index (PSQI), a measure of sleep health, was used (Niu et al., 2023). Additionally, (8) total effective rate (TER) is a secondarily processed, unverified result based on specific evaluation criteria, such as improvement in clinical symptoms or other quantifiable outcomes. TER was consistently calculated based on the formula: *TER = (N3 + N2 + N1)/N* where *N3, N2, N1*, and *N* represent the numbers of participants classified as “healed,” “significantly improved,” “improved,” and the total sample size, respectively.

### 2.3 Study selection

After eliminating duplicate publications, two researchers (JRB, HWJ) independently reviewed the titles and abstracts to assess their relevance. The full texts of potentially eligible studies were then examined for the final selection. Any disagreements were resolved through discussion with another researcher (JTL). For this review, a formal inter-rater agreement statistic, such as Cohen’s kappa, was not calculated; instead, any discrepancies between reviewers were resolved through discussion and consensus with a third author. All retrieved studies were organized and managed using Zotero (Roy Rosenzweig Center for History and New Media, George Mason University).

### 2.4 Data extraction

Two researchers (JRB, HWJ) independently extracted data using standardized forms, followed by cross-verification. In case of conflict, a third researcher (JTL) was consulted to reach a consensus. For this review, a formal inter-rater agreement statistic, such as Cohen’s kappa, was not calculated; instead, any discrepancies between reviewers were resolved through discussion and consensus with a third author. The extracted information included the first author’s name, publication year, sample size, dropout number, participants, HM details, control interventions, intervention duration, outcome measures, and adverse events related to the intervention. If the data were insufficient or unclear, additional information was requested from the corresponding authors via email.

### 2.5 Quality assessment

Two researchers (JRB, HWJ) independently analyzed the methodological quality and quality of evidence for each outcome of all included studies. Discrepancies were resolved through discussions with another researcher (JTL).

Methodological quality was assessed using the revised Cochrane Collaboration risk-of-bias tool for randomized trials (RoB2). The analysis involved the following domains: random sequence generation, allocation concealment, blind participants and personnel, blind outcome assessment, incomplete outcome data, selective reporting, and other potential biases. Each domain was categorized as “low risk,” “unclear,” or “high risk.” In the domain of random sequence generation, a study was considered to have “some concerns” or higher risk of bias if the term “randomization” was mentioned without an explanation of the randomization method or if there was no confirmation of double-blinding, even with an explanation.

The GRADE methodology was employed to assess the quality of evidence for each outcome. GRADEpro, an online software (https://gradepro.org/), was used to analyze risk of bias, inconsistency, indirectness, imprecision, and publication bias on a four-point scale (“very low,” “low,” “moderate,” or “high”).

#### 2.5.1 Quality assessment of intervention

Given that Soyo-san is a complex herbal intervention, assessing the quality and heterogeneity of preparations in the included studies is essential for the validity of this review. The Consensus statement on the Phytochemical Characterisation of Medicinal Plant extracts (ConPhyMP) provides guidelines for transparent reporting by defining standards for plant materials, preparation processes, and chemical profiling (Heinrich et al., 2022). In this review, we applied the ConPhyMP checklists to assess the reporting quality of all included studies.

### 2.6 Data synthesis and analysis

RStudio (version 2023.06.1 + 524; Posit, Boston, MA, United States) and R (version 4.3.0; CRAN, WU Vienna), with the ‘meta’, ‘metafor’, and ‘dmetar’ packages for meta-analysis, and the ‘robvis’ package for visualizing the risk of bias assessments. Descriptive statistical analyses were performed for participant characteristics, interventions, and outcomes across all the included studies. Meta-analyses were performed on studies in which the treatment and control groups were within the same category, and separate analyses were conducted for each outcome variable. The main method for meta-analysis in the quantitative synthesis utilized a random-effects model, considering the inherent heterogeneity in clinical trials of traditional East Asian medicine (TEAM). The results of the common-effects model were presented for sensitivity analysis. Due to the differences in treatment components, study groups, and patient selection criteria among the included studies, potential heterogeneity in the actual treatment effects was anticipated, making the random-effects model more appropriate. Continuous outcomes were recorded as mean differences (MDs) with 95% confidence intervals (CIs). For dichotomous data, the relative risk (RR) was the primary analysis method, with odds ratios (ORs) provided as supplementary information. Point estimates and 95% CIs were used for representation. Heterogeneity among the effect sizes of the studies was assessed using the *I* ^2^ statistic, with values > 75% indicating high heterogeneity.

### 2.7 Subgroup analysis

Subgroup analyses were conducted based on the following criteria to account for heterogeneity or to analyze whether effect sizes were significantly different among subgroups, provided there were sufficient data: (1) type of antidepressant used in the control group, (2) treatment duration, (3) use of TEAM diagnostic tools, (4) use of HAMD criteria for participant selection, and (5) type of Soyo-san substance. Additionally, meta-regression analyses were conducted using treatment duration as a moderating variable to assess its impact on effect size.

### 2.8 Sensitivity analysis

A cumulative meta-analysis was conducted to analyze changes in effect size chronologically and by sample size. Additionally, leave-one-out analysis was performed to compare the effect size or heterogeneity when each study was included or excluded.

### 2.9 Publication bias

Publication bias was assessed for outcome measures in >10 included studies using funnel plots. Asymmetry in the funnel plots was evaluated, and if observed, Egger’s regression was performed using the metabias () function from the ‘meta’ package (considered present if *P* < 0.05). The trim-and-fill method was used to assess the degree of publication bias using the trimfill () function, and funnel plots before and after adjustment are presented.

## 3 Results

### 3.1 Description of included studies

**FIGURE 1:**
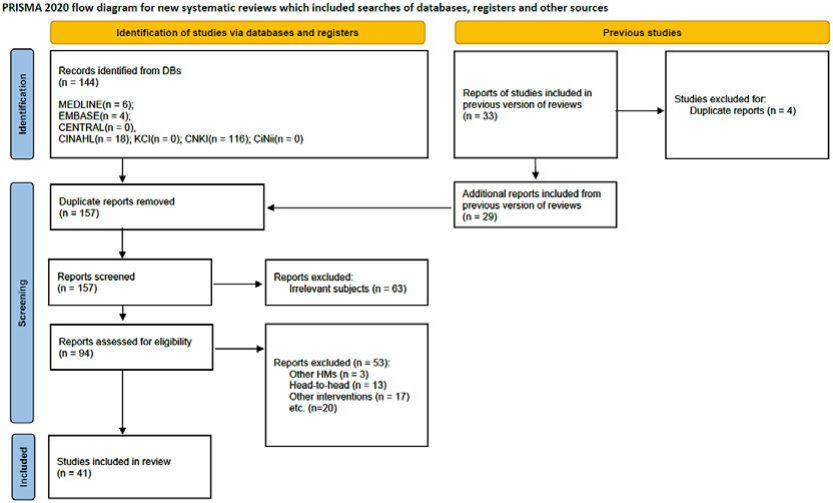
PRISMA Flow diagram.

After the exclusion of duplicates, the database searches identified 144 studies, and 33 studies were identified from reference lists of previous systematic reviews. After screening the titles and abstracts for relevance, 94 studies were excluded. Upon full-text review, three studies used HM other than Soyo-san, 13 studies compared HM alone with antidepressants, 17 studies employed treatments other than HM in the intervention group, or in combination with HM, or used treatments other than antidepressants in the control group. Twenty studies were excluded for various reasons. Finally, 41 RCTs involving 3,628 participants were included (Figure 1) (Supplementary Table S2).

The overall characteristics of the included studies are summarized in Table 2. All the RCTs were conducted in China and published in academic journals. The sample size ranged from 40 to 400 participants, with a median age of 88. The treatment duration was 2–12 weeks. Few studies applied TEAM pattern identification to categorize the signs and symptoms of each participant consistently to optimize treatment. Liver qi stagnation or phlegm in the heart in 3 studies (Xu, 2006; Ma, 2010; Gao, 2013), liver qi stagnation in 5 studies (Xu et al., 2011; Lu and Chen, 2013; Shao et al., 2017; Han et al., 2019; Hu et al., 2024), liver qi stagnation and spleen deficiency in 1 study (Jiang and Liu, 2019), liver depression and spleen deficiency in 1 study (Zhao D. et al., 2020a), and liver depression type of fire in 2 studies (Zhao S. et al., 2020c; Chen et al., 2021) were adopted as the TEAM pattern. Few studies limited participants’ conditions by severity of depression based on HAMD: mild or greater (Li et al., 2011; Lin et al., 2015; Cui et al., 2017; Xu et al., 2017; Yang et al., 2018; Zeng et al., 2018; Gong et al., 2020; Zhao D. et al., 2020a; Zhao S. et al., 2020c), moderate or greater (Li and Gao, 2006; Song and Yang, 2008; Wang, 2008; Zou et al., 2009; 2013; Zhang, 2010; Xu et al., 2011; Lu and Chen, 2013; Wang and Ni, 2014; Zhang et al., 2014; Yuan, 2015; Zhang and Zhang, 2016; Shao et al., 2017; Han et al., 2019; Wang et al., 2019), mild and moderate levels of depression (Jiang and Liu, 2019). Several studies specified the duration of PSD by indicating the time of onset before the intervention (Song and Yang, 2008; Zou et al., 2009; 2013; Ma, 2010; 2015; Zhang, 2010; Li et al., 2011; Xu et al., 2011; Zhou, 2017; Zhao D. et al., 2020a; Hu et al., 2024). In the control group, three types of antidepressants were used: SSRIs in 30 studies, flupentixol/melitracen in 9 studies (Zou et al., 2009; Li et al., 2011; Wang and Ni, 2014; Zhi et al., 2014; Lin et al., 2015; Ma, 2015; Zhang and Li, 2016; Han et al., 2019; Zhao D. et al., 2020a), and serotonin and norepinephrine reuptake inhibitors in 2 studies (Zou et al., 2013; Zhou, 2017). HAMD was the most frequently used outcome measure, followed by TER in 34 studies, MBI in 8 studies (Li and Gao, 2006; Song and Yang, 2008; Zhang and Li, 2016; Zhang and Zhang, 2016; Cui et al., 2017; Han et al., 2019; Zhao S. et al., 2020c; Zheng, 2021), 5-HT levels in 7 studies (Yang et al., 2018; Zeng et al., 2018; Han et al., 2019; Jiang and Liu, 2019; Wang et al., 2019; Zhao D. et al., 2020a; Zheng, 2021), SSS in 7 studies (Li and Gao, 2006; Song and Yang, 2008; Zou et al., 2009; 2013; Dang and Zhang, 2011; Zhang and Li, 2016; Zheng, 2021), NIHSS in 3 studies (Cui et al., 2017; Wang et al., 2019; Zhao D. et al., 2020a), PSQI in 3 studies (Yang et al., 2018; Zhao D. et al., 2020a; Zheng, 2021), FMA in 2 studies (Yang et al., 2018; Zheng, 2021), and MMSE in 2 studies (Song and Yang, 2008; Zhao S. et al., 2020c). TER was calculated based on the reduction of HAMD score only or together with symptom improvement in 28 studies, and solely on symptoms in 6 studies (Supplementary Table S3).

**TABLE 2:** Characteristics of the included studies.

Study ID	Sample size (included→analyzed)	Sex (Male/Female)	Mean age (range) (y)	Diagnostic tool for PSD/Stroke (Pattern identification) (inclusion criteria)	PSD duration	Baseline HAMD (mean ± SD) (scores) (severity)	(A) Treatment intervention (treatment period; /Follow-up period)	(B) Control intervention (antidepressant class)	Outcomes and results (post treatment)
2006_Li (Li and Gao, 2006)	85 (43:42)→ 85 (43:42)	(A) 23/20 (B) 22/20	(A)69.53 ± 7.87 (45–81) (B)68.23 ± 7.35 (47–80)	CCMD-Ⅱ-R/CT, MRI (HAMD≧18)^⒡^	NR	(A)22.3 ± 3.8 (18–29) (B)22.7 ± 3.3 (18–30)	SYS bid-tid+(B) (8 weeks; 4 weeks+4 weeks/NA)	(1) fluoxetine 20 mg qd (SSRI) (2) Psychotherapy	1. HAMD^⒝^ 2. SSS^⒝^, MBI^⒝^ 3. TER^⒝^ 4. Neurological Rehabilitation Efficacy (SSS, MBI)^⒝^
2006_Xu (Xu, 2006)	70^⒟^(35:35)→ 70^⒟^(35:35)	(A) 21/14 (B) 19/16	(A)55.2 ± 1.9 (B)56.2 ± 2.9	CCMD^⒠^/⒜, CT, MRI (Liver qi stagnation, phlegm in the heart)^⒡^	NR	(A)22.81 ± 3.63 (B)21.23 ± 2.96	SYS bid+(B) (8 weeks; 4 weeks+4 weeks/NA)	fluoxetine 20 mg qd (SSRI)	1. TER^⒝^ 2. HAMD^⒝^
2008_Song^⒥^ (Song and Yang, 2008)	72 (36:36)→ 72 (36:36)	(A) 19/17 (B) 20/16	(A)61.1 ± 10.2 (44–70) (B)63.4 ± 10.6 (46–77)	CCMD-3/CT, MRI (HAMD>17)^⒡^	(A) 73-78 days (B) 71-80 days	(A)29.52 ± 7.32 (B)28.24 ± 6.27	SYS^⒢^ bid+(B) (12 weeks; 4 weeks+8 weeks/NA)	Fluoxetine 20 mg qd (SSRI)	1. TER^⒝^ 2. HAMD^⒝^ 3. MMSE^⒝^ 4. SSS^⒝^ 5. BI^⒝^
2008_Wang (Wang, 2008)	72 (36:36)→ 72 (36:36)	(A) 19/17 (B) 18/18	(A)68.3 ± 7.3 (55–79) (B)69.3 ± 7.8 (56–83)	CCMD-3/⒜, CT, MRI (HAMD>17)^⒡^	NR	(A)28.34 ± 6.27 (B)29.52 ± 7.31	SYS bid+(B) (3 months/NA)	Fluoxetine 20 mg qd (SSRI)	1. TER 2. HAMD^⒦^ 3. Neurological Rehabilitation Efficacy (MESSS)^⒝^
2009_Zou (Zou et al., 2009)	60 (30:30)→ 60 (30:30)	(A) 18/12 (B) 19/11	(A)67.9 ± 6.1 (B)66.8 ± 7.1	CCMD^⒠^/⒜, CT, MRI (HAMD≧18)^⒡^	(A) 27 ± 7.5 months^⒣^ (B) 26 ± 6.7 months^⒣^	(A)27.38 ± 3.80 (B)27.50 ± 3.75	SYS tid+(B) (6 weeks; 3 weeks+3 weeks/NA)	flupentixol + melitracen bid (TCA)	1. HAMD^⒝^ 2. SSS^⒝^ 3. TER^⒝^
2010_Ma (Ma, 2010)	80 (40:40)→ 80 (40:40)	(A) 26/14 (B) 24/16	(A)55.2 ± 1.9 (B)56.2 ± 2.9	CCMD^⒠^/CT, MRI (Liver qi stagnation, phlegm in the heart)^⒡^	(A) 10.5 ± 6.9 months^⒣^ (B) 9.5 ± 3.2 months^⒣^	(A)22.81 ± 3.63 (B)21.23 ± 2.96	SYS^⒢^ bid+(B) (4 weeks; 2 weeks+2 weeks/NA)	Fluoxetine 20 mg qd (SSRI)	1. TER^⒝^ 2. HAMD
2010_Zhang (Zhang, 2010)	54 (36:18)→ 54 (36:18)	(A) 21/15 (B) 11/7	(A)53.24 ± 6.31 (B)54.36 ± 4.42	DSM-IV^⒡^/CT, MRI (HAMD≧20)	(A) 8.22 ± 0.65 months (6–41 months) (B) 9.01 ± 0.54 months (6–38 months)	(A)40.22 ± 3.31 (B)40.44 ± 3.43	SYS bid+(B) (2 months/NA)	Paroxetine 20 mg qd (SSRI)	1. TER^⒝^ 2. HAMD^⒝^ 3. Neurological damage score^⒝^
2011_Xu (Xu et al., 2011)	60 (30:30)→ 60 (30:30)	(A) 18/12 (B) 20/10	(A)49.05 ± 6.27 (42–73) (B)48.86 ± 5.93 (43–75)	CCMD-3/⒜, CT, MRI (Liver qi stagnation) (HAMD>17)^⒡^	(A) 18.50 ± 4.81 months (0.5–36 months) (B) 19.07 ± 5.02mo nths(0.5–38 months)	NA	SYS bid+(B) (4 weeks/NA)	Fluoxetine 20 mg qd (SSRI)	1. TER^⒝^
2011_Dang (Dang and Zhang, 2011)	102 (52:50)→ 102 (52:50)	(A) 27/25 (B) 25/25	(A)62.4 ± 6.3 (B)64.0 ± 5.2	CCMD-3/⒜, CT, MRI^⒡^	NR	(A)18.92 ± 1.50 (B)19.09 ± 1.76	SYS bid+(B) (4 weeks/NA)	Paroxetine 20 mg qd (SSRI)	1. SSS^⒝^ 2. HAMD^⒝^
2011_Li (Li et al., 2011)	66 (34:32)→ 66 (34:32)	(A) 15/19 (B) 15/17	(A) (43–70) (B) (44–70)	CCMD-3/⒜, CT, MRI^⒡^ (HAMD≧8)	(A) 10-52 days (B) 9-51 days	(A)17.97 ± 5.27 (B)17.63 ± 5.27	SYS^⒢^ bid+(B) (6 weeks; 2 weeks+2 weeks+2 weeks/NA)	flupentixol + melitracen bid (TCA)	1. TER^⒝^ 2. HAMD^⒝^
2012_Pan (Pan and Wan, 2012)	60 (30:30)→ 60 (30:30)	(A) 13/17 (B) 14/16	(A)56.20 ± 8.6 (B)54.6 ± 17.5	CCMD-3/⒜, CT, MRI	NR	(A)20.2 ± 3.8 (B)19.7 ± 4.2	SYS bid+(B) (8 weeks; 2 weeks+2 weeks+4 weeks/NA)	Fluoxetine 20 mg qd (SSRI)	1. HAMD^⒝^ 2. TER^⒝^
2013_Zou (Zou et al., 2013)	80 (40:40)→ 80 (40:40)	(A) 26/14 (B) 26/14	(A)67.2 ± 5.9 (B)66.9 ± 5.7	CT, MRI^⒡^ (HAMD≧18)	≧2 weeks	(A)28.42 ± 4.11 (B)27.97 ± 4.04	SYS tid+(B) (4 weeks; 2 weeks+2 weeks/NA)	Venlafaxin 75 mg qd (SNRI)+Oryzanolum 20 mg tid	1. HAMD^⒝^ 2. SSS^⒝^ 3. TER^⒝^
2013_Lu (Lu and Chen, 2013)	76 (39:37)→ 76 (39:37)	(A) 24/15 (B) 22/15	(A)56.28 ± 2.14 (B)57.23 ± 1.92	CCMD^⒠^/⒜, CT, MRI (Liver qi stagnation)^⒡^	NR	(A)24.52 ± 2.84 (18–30) (B)27.96 ± 2.78 (18–29)	SYS^⒢^ bid+(B) (4 weeks/NA)	Paroxetine 20 mg qd (SSRI)	1. TER^⒝^ 2. HAMD^⒝^
2013_Li (Li, 2013)	64 (32:32)→ 64 (32:32)	(A) 19/13 (B) 20/12	(A)53.24 ± 6.31 (B)54.6 ± 4.42	DSM^⒠^/⒜, CT, MRI (HAMD≧20)	(A) 8.22 ± 0.65 months (B) 9.01 ± 0.54 months	NA	SYS^⒢^ bid+(B) (60 days/NA)	Fluoxetine 20 mg qd (SSRI)	1. TER^⒝^
2013_Gao (Gao, 2013)	63 (32:31)→ 63 (32:31)	(A) 13/19 (B) 12/19	(A)60.3 ± 6.7 (B)61.2 ± 6.2	CCMD^⒠^/⒜, CT, MRI (Liver qi stagnation, phlegm in the heart)^⒡^	NR	(A)21.25 ± 1.78 (B)21.63 ± 1.83	SYS bid+(B) (8 weeks; 4 weeks+4 weeks/NA)	Mirtazapine 15 mg qd	1. TER^⒝^ 2. HAMD^⒝^
2014_Zhang (1) (Zhang, 2014)	100 (50:50)→ 100 (50:50)	NR (40/60)	NR (45.8 ± 19.7)	NA	NR	NA	SYS bid+(B) (8 weeks/NA)	Fluoxetine 20 mg qd (SSRI)	1. TER^⒝^ 2. SSS rate^⒝^ 3. ADL^⒝^
2014_Zhang (2) (Zhang et al., 2014)	80 (40:40)→ 80 (40:40)	(A) 28/12 (B) 30/10	(A)62.6 ± 4.5 (47–73) (B)63.1 ± 5.1 (49–74)	HAMD>17^⒡^	NR	NA	SYS bid (2w) →qd +(B) (8 weeks; 2 weeks+2 weeks+2 weeks +2 weeks/NA)	Sertraline 50 mg (3 days) →100 mg qd (SSRI)	1. HAMD reduction rate^⒝^ 2. TER^⒝^
2014_Zhi (Zhi et al., 2014)	110 (55:55)→ 110 (55:55)	NR (59/51)	(A)63.26 ± 8.35 (B)62.79 ± 7.92	CCMD-3/⒜, CT, MRI^⒡^	NR	(A)28.32 ± 4.27 (B)28.93 ± 5.31	SYS tid+(B) (8 weeks/NA)	flupentixol + melitracen bid (TCA)	1. HAMD^⒝^ 2. HAMA^⒝^ 3. TER (HAMD)^⒝^ 4. TER (HAMA)^⒝^
2014_Wang (Wang and Ni, 2014)	112 (60:52)→ 112 (60:52)	(A) 35/25 (B) 33/19	(A)55.1 (45–75) (B)56.7 (49–78)	CCMD^⒠^/⒜, CT, MRI (HAMD≧18)^⒡^	NR	(A)24.75 ± 8.82 (B)25.20 ± 7.25	SYS tid+(B) (14 days/NA)	flupentixol + melitracen bid (TCA)	1. TER^⒝^ 2. HAMD^⒝^
2015_Ma (Ma, 2015)	132 (68:64)→ 132 (68:64)	(A) 30/38 (B) 30/71	(A)43.12 ± 1.12 (42–46) (B)52.34 ± 0.78 (45–71)	CCMD-3/⒜, CT, MRI^⒡^	(A) 29.37 ± 1.21 days(11–53) (B) 30.25 ± 1.03 days(10–50)	(A)17.97 ± 5.27 (B)17.63 ± 5.27	SYS bid+(B) (42 days; 12 days + 12 days + 12 days/NA)	Flupentixol 0.5 mg + melitracen 10 mg bid (TCA)	1. HAMD^⒝^ 2. TER^⒝^
2015_Lin (Lin et al., 2015)	58 (29:29)→ 58 (29:29)	NR (37/21)	NR (62.2 ± 7.1)	CCMD-3^⒡^ (HAMD>7)	NR	(A)23.44 ± 5.49 (B)23.56 ± 6.29	SYS bid+(B) (4 weeks; 1 weeks+1week+1week+1week/NA)	Flupentixol + melitracen bid (TCA)	1. TER^⒝^ 2. HAMD^⒝^
2015_Li (Li, 2015)	68 (34:34)→ 68 (34:34)	NR (36/32)	NR (57.3 ± 2.2) (38–76)	CCMD-3/⒜, CT, MRI^⒡^	NR	(A)27.12 ± 2.48 (B)26.39 ± 2.48	SYS bid+(B) (8 weeks; 1 weeks+1week+2 weeks+2 weeks+2 weeks/NA)	Escitalopram 5 mg (3 days)→10 mg qd (SSRI)^⒤^	1. HAMD^⒝^ 2. Hcy^⒝^ 3. TC, TG, HDL-C, LDL-C
2015_Yuan (Yuan, 2015)	80 (40:40)→ 80 (40:40)	(A) 24/16 (B) 22/18	(A)49.05 ± 0.25 (41–74) (B)49.85 ± 5.85 (43–75)	CCMD-3/⒜, CT, MRI^⒡^ (35≧HAMD≧18)	NR	(A)28.41 ± 1.49 (B)28.12 ± 1.36	SYS qd+(B) (4 weeks/NA)	Escitalopram 10 mg qd (SSRI)	1. HAMD^⒝^ 2. TCM Symptom Score^⒝^
2016_Zhang (1) (Zhang and Li, 2016)	80 (40:40)→ 80 (40:40)	(A) 21/19 (B) 20/20	(A)55.2 ± 1.6 (B)55.6 ± 1.9	CCMD^⒠^/⒜, CT, MRI^⒡^	NR	(A)28.54 ± 3.72 (B)28.71 ± 3.63	SYS+(B) (8 weeks; 4 weeks+4 weeks/NA)	Flupentixol + melitracen (TCA)	1. HAMD^⒝^ 2. SSS^⒝^ 3. MBI^⒝^ 4. TER^⒝^
2016_Zhang (2) (Zhang and Zhang, 2016)	92 (46:46)→ 92 (46:46)	(A) 26/20 (B) 25/21	(A)57.6 ± 6.5 (40–79) (B)58.7 ± 6.9 (41–78)	CCMD-3/⒜, CT, MRI^⒡^ (HAMD>17)	NR	(A)23.39 ± 4.12 (B)23.41 ± 4.21	SYS bid+(B) (30 days; 15 days + 15 days/NA)	Fluoxetine 20 mg qd (SSRI)	1. HAMD^⒝^ 2. BI^⒝^
2017_Shao (Shao et al., 2017)	58 (29:29)→ 58 (29:29)	NA	(A)64.7 ± 8.9 (48–79) (B)63.5 ± 8.5 (45–77)	CCMD-3/⒜, CT, MRI^⒡^ (Liver qi stagnation) (HAMD≧17)	NR	(A)23.44 ± 5.49 (B)23.56 ± 6.29	SYS bid+(B) (8 weeks/NA)	Citalopram 20 mg qd (SSRI)	1. TER^⒝^ 2. HAMD^⒝^
2017_Zhou (Zhou, 2017)	68 (34:34)→ 68 (34:34)	(A) 19/15 (B) 18/16	(A)45.9 ± 5.1 (18–68) (B)45.8 ± 4.8 (18–68)	Symptoms^⒡^	(A) 2.6 ± 0.7 months (B) 2.5 ± 0.8 months	(A)29.6 ± 2.5 (B)29.5 ± 2.8	SYS tid+(B) (4 weeks/NA)	Venlafaxine 75 mg qd (SNRI)	1. TER^⒝^ 2. HAMD^⒝^
2017_Xu (Xu et al., 2017)	400 (200:200)→ 400 (200:200)	(A) 110/90 (B) 106/95^⒞^	(A)64.5 ± 4.5 (B)45.8 ± 4.8	CCMD/⒜^⒡^ (HAMD>8)	NR	(A)22.78 ± 2.17 (B)21.19 ± 3.25	SYS bid+(B) (8 weeks; 4 weeks+4 weeks/NA)	Fluoxetine qd (SSRI)	1. TER^⒝^ 2. HAMD^⒝^
2017_Sun (Sun, 2017)	102 (51:51)→ 102 (51:51)	(A) 28/23 (B) 27/24	(A)54.0 ± 3.8 (40–68) (B)55.3 ± 4.2 (41–70)	Guidelines for the treatment of depression/Chinese Guidelines for the diagnosis and treatment of acute ischemic stroke, CT, MRI^⒡^	NR	(A)28.5 ± 2.3 (B)28.9 ± 2.8	SYS^⒢^ bid+(B) (60 days/NA)	Fluoxetine 20 mg qd (SSRI)	1. TER^⒝^ 2. HAMD^⒝^
2017_Cui (Cui et al., 2017)	44^⒟^ (22:22)→ 44^⒟^ (22:22)	(A) 15/7 (B) 13/9	(A)63.55 ± 8.11 (49–77) (B)68.82 ± 5.45 (59–77)	CCMD-3/Diagnostic efficacy criteria for Chinese medical conditions, CT, MRI^⒡^ (35>HAMD>8)	NR	(A)27.27 ± 3.09 (B)27.95 ± 2.57	SYS bid+(B) (4 weeks/NA)	citalopram 20 mg qd (SSRI)	1. HAMD^⒝^ 2. TER^⒝^ 3. NIHSS 4. BI
2018_Yang (Yang et al., 2018)	40 (20:20)→ 40 (20:20)	(A) 10/10 (B) 11/9	(A)53.83 ± 3.9 (B)54.32 ± 4.3	CT, MRI^⒡^ (HAMD≧8)	NR	(A)14.63 ± 2.23 (B)14.49 ± 3.25	SYS bid+(B) (8 weeks; 4 weeks+4 weeks/NA)	Sertraline 25 mg⒢ qd (SSRI)	1. FMA^⒝^ 2. HAMD^⒝^ 3. SQI^⒝^ 4. 5-HT^⒝^ 5. NE^⒝^ 6. 5-HIAA^⒝^
2018_Zeng (Zeng et al., 2018)	86 (43:43)→ 86 (43:43)	(A) 23/20 (B) 25/18	(A)57.53 ± 4.70 (33–79) (B)59.10 ± 5.33 (36–77)	CCMD-3/⒜^⒡^ (HAMD≧8)	NR	(A)21.02 ± 2.84 (B)20.75 ± 4.17	SYS tid+(B) (30 days; 15 days + 15 days/NA)	Fluoxetine 20 mg qd (SSRI)	1. TER^⒝^ 2. HAMD^⒝^ 3. 5-HT^⒝^
2019_Han (Han et al., 2019)	60 (30:30)→ 60 (30:30)	(A) 16/14 (B) 18/12	(A)55.77 ± 7.16 (47–72) (B)56.13 ± 8.21 (45–75)	CCMD-3^⒡^ (Liver qi stagnation) (HAMD≧18)^⒡^	NR	(A)29.83 ± 3.75 (B)29.63 ± 4.99	SYS⒢ bid+(B) (4 weeks; 1 weeks+1week+1week+1week/NA)	Flupentixol 0.5 mg + melitracen 10 mg bid (TCA)	1. TER^⒝^ 2. HAMD^⒝^, BI^⒝^ 3. 5-HT, NE, DA^⒝^
2019_Jiang (Jiang and Liu, 2019)	148 (74:74)→ 148 (74:74)	(A) 42/32 (B) 40/34	(A)51.23 ± 2.36 (36–76) (B)51.15 ± 2.42 (36–76)	CCMD-3/Acute Ischemic Brain in China Stroke Diagnosis and Treatment Guidelines 2014, CT, MRI^⒡^ (Liver qi stagnation and Spleen deficiency) (23≥HAMD≥8)	NR	(A)23.79 ± 4.47 (B)23.56 ± 4.54	SYS bid+(B) (4 weeks; 2 weeks+2 weeks/NA)	Fluoxetine 20 mg qd (SSRI)	1. TER^⒝^ 2. HAMD^⒝^, BI^⒝^ 3. Neurotransmitter power^⒝^ 4. 5-HT, NE^⒝^ 5. TNF-α,IL-1β,IL-6^⒝^
2019_Wang (Wang et al., 2019)	80 (40:40)→ 80 (40:40)	(A) 20/20 (B) 27/13	(A)55.0 ± 1.4 (40–75) (B)55.5 ± 1.5 (40–74)	CCMD-3/CT, MRI^⒡^ (HAMD>17)	NR	(A)17.5 ± 2.5 (B)17.6 ± 2.7	SYS^⒢^ bid+(B) (60 days; 15 days + 15 days + 15 days + 15 days/NA)	Escitalopram 20 mg bid (SSRI)	1. TER^⒝^ 2. NIHSS^⒝^, HAMD^⒝^, HAMA^⒝^ 3. 5-HT^⒝^, IL-1^⒝^, Hcy^⒝^
2020_Zhao (1) (Zhao D. et al., 2020a)	70 (35:35)→ 70 (35:35)	(A) 21/14 (B) 22/13	(A)55.1 ± 5.9 (40–73) (B)54.6 ± 5.3 (42–71)	CCMD-3/CT, MRI^⒡^ (Liver depression and Spleen deficiency) (HAMD>7)	(A) 7.2 ± 3.5 months^⒣^ (1–20) (B) 6.9 ± 3.2 months^⒣^ (1–20)	(A)19.37 ± 2.15 (B)18.92 ± 1.76	SYS bid+(B) (4 weeks/NA)	Flupentixol 0.5 mg + melitracen 10 mg bid (TCA)	1. TER^⒝^ 2. HAMD^⒝^ 3. PSQI^⒝^ 4. NIHSS^⒝^ 5. Serum amine neurotransmitters levels (NE, 5-HT, DA)^⒝^
2020_Zhao (2) (Zhao S. et al., 2020c)	138 (69:69)→ 138 (69:69)	(A) 33/36 (B) 35/34	(A)66.86 ± 9.37 (B)67.12 ± 9.77	CCMD-3/CT, MRI^⒡^ (Liver depression type of fire) (HAMD≥8)	NR	(A)22.70 ± 1.87 (B)22.94 ± 1.56	SYS bid+(B) (8 weeks/NA)	Escitalopram 10 mg qd (SSRI)	1. HAMD^⒝^, MMSE^⒝^ 2. BI^⒝^, FAQ
2020_Gong (Gong et al., 2020)	100 (50:50)→ 100 (50:50)	(A) 31/19 (B) 28/22	(A)56.00 ± 6.20 (35–75) (B)57.00 ± 5.12 (37–75)	CCMD-3^⒡^ (35≥HAMD≥8)	NR	(A)28.3 ± 2.8 (B)27.8 ± 2.5	SYS^⒢^ bid+(B) (2 months/NA)	Fluoxetine 20 mg qd (SSRI)	1. TER^⒝^ 2. HAMD^⒝^
2021_Chen (Chen et al., 2021)	100 (50:50)→ 100 (50:50)	(A) 26/24 (B) 27/23	(A)74.2 ± 3.1 (60–84) (B)74.0 ± 3.0 (60–82)	(Liver depression type of fire)	NR	(A)22.50 ± 1.68 (B)22.49 ± 1.70	SYS tid+(B) (8 weeks; 4 weeks+4 weeks/NA)	Escitalopram 10 mg qd (SSRI)	1. HAMD^⒝^ 2. TER^⒝^ 3. Satisfaction^⒝^
2021_Zheng (Zheng, 2021)	50 (25:25)→ 50 (25:25)	(A) 14/11 (B) 13/12	(A)47.70 ± 5.81 (30–66) (B)48.52 ± 6.31 (31–69)	Symptoms	NR	(A)27.93 ± 3.77 (B)28.24 ± 3.78	SYS bid/tid+(B) (7 weeks; 3 weeks+4 weeks/NA)	Sertraline 50 mg (3 days) →100 mg qd (SSRI)	1. HAMD^⒝^, SSS^⒝^, SQI^⒝^ 2. FMA^⒝^, MBI^⒝^ 3. 5-HIAA^⒝^, 5-HT^⒝^, NE^⒝^
2024_Hu (Hu et al., 2024)	108 (54:54) →108 (54:54)	(A) 30/24 (B) 28/26	(A)58.96 ± 3.31 (43–78) (B)58.71 ± 3.59 (41–75)	Chinese expert consensus on clinical practice of post-stroke depression, 2016 (Liver qi stagnation)	(A) 11.53 ± 1.59 months (6–21) (B) 11.48 ± 1.67 months (5–19)	(A)19.13 ± 2.24 (B)18.95 ± 2.36	SYS bid+(B) (8 weeks/NA)	Escitalopram 20 mg qd (SSRI)	1. HAMD^⒝^ 2. TER

Abbreviations: 5-HIAA, 5-hydroxyindoleacetic acid; 5-HT, 5-hydroxytryptamine (serotonin); BI, Barthel index; bid, twice a day; CCMD-3: Chinese Classification and Diagnostic Criteria of Disorders(中国精神障碍分类与诊断标准), 2001; CT, computed tomography; DA, dopamine; DSM, Diagnostic and Statistical Manual of Mental Disorders; FMA, Fugl-Meyer Assessment Scale; HAMA, Hamilton Anxiety Scale; HAMD, Hamilton Depression Scale; Hcy, homocysteine; HDL-C, high-density lipoprotein cholesterol; IL, interleukin; LDL-C, low-density lipoprotein cholesterol; MBI, Modified Barthel Index; MMSE, Mini-Mental State Examination; MRI, magnetic resonance imaging; NA: data not available; NE, norepinephrine; NIHSS, National Institutes of Health Stroke Scale; NR: not reported, although it is mentioned as a result; PSD, post-stroke depression; PSQI, Pittsburgh Sleep Quality Index; qd, once a day; SD, standard deviation; SQI, Sleep Quality Index; SSRI, selective serotonin reuptake inhibitor; SSS, Scandinavian Stroke Scale; SYS, Soyo-san; TC, total cholesterol; TCA, tricyclic antidepressant; TCM, Traditional Chinese Medicine; TER, total effective rate; TG, triglyceride; tid, three times a day; TNF, tumor necrosis factor.

⒜ Diagnostic Criteria for Cerebral Hemorrhage and Cerebral Infarction formulated by the 4th National Academic Conference on Cerebrovascular Diseases. ⒝ Significantly effective outcomes or results. ⒞ Correct number unknown. ⒟ When the numbers of participants (Total, Each group) were inconsistent, we selected the one that was consistently used for outcomes. ⒠ Version unclear. ⒡ No mental disorder, depression history, or intellectual impairment. ⒢ Custom formula, without mention of detailed ingredients. ⒣ No mention if this duration is for PSD or Stroke, but the context suggests it is for PSD. ⒤ Can be taken up to 20 mg if needed, without mention of criteria. ⒥ 3-arm study. ⒦ Excluded from data analysis due to low reliability; treatment group change in HAMD score was smaller than that of Control group, but reduction percentage was larger (corresponding author could not be contacted regarding this discrepancy).

### 3.2 Methodological quality

**FIGURE 2:**
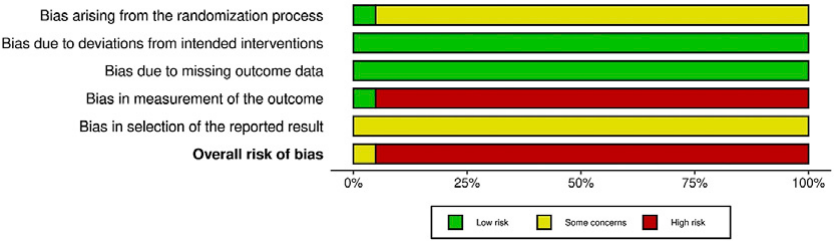
RoB2 summary plot.

Based on the RoB2 analysis, 2 studies (Zhi et al., 2014; Zhou, 2017), used appropriate random sequence generation methods and explicitly conducted double-blinding, indicating a low risk of bias in the randomization process and outcome domains. In the remaining 39 studies, there was either no description of random sequence generation or insufficient information regarding allocation concealment and blinding of participants, personnel, or outcome assessors, resulting in some level of bias. No dropouts occurred in any of the included studies, indicating a low risk of bias due to deviation from the intended interventions. Regarding missing outcome data, almost all studies had complete data for the intended interventions, indicating a low risk of bias. The remaining 39 studies lacked protocols, indicating a high risk of bias. Lack of confirmation of the measurements before unblinding caused ‘some concerns’ about bias due to selection of the reported results. Overall, 2 studies were rated as having ‘some concerns’ about bias, whereas the rest had a high level of bias (Figure 2) (Supplementary Figure S1).

### 3.3 Details of Soyo-san administration

Twenty-seven studies used decoctions, 7 used pills (Wang, 2008; Zou et al., 2009; Wang and Ni, 2014; Zhang, 2014; Zhi et al., 2014; Zeng et al., 2018; Hu et al., 2024), and 7 used granules (Xu, 2006; Gao, 2013; Jiang and Liu, 2019; Xu et al., 2017; Zou et al., 2013; Zhou, 2017; Zhang and Li, 2016). Soyo-san was provided twice a day in 29 studies, three times a day in 7 studies (Zou et al., 2009; 2013; Wang and Ni, 2014; Zhi et al., 2014; Zeng et al., 2018; Chen et al., 2021), and 2 studies provided a combination of two and three times a day during different treatment periods (Li and Gao, 2006; Zheng, 2021). One study combined once a day and twice a day administration (Zhang et al., 2014), one study had once daily administration (Yuan, 2015), and one study did not specify dosing instructions (Zhang and Li, 2016). In addition to the original Soyo-san composition, 22 additional herbs were used. Except for 5 studies with unknown dosages of each herb (Wang, 2008; Zou et al., 2009; 2013; Gao, 2013; Zeng et al., 2018) and 6 other studies using medications with unknown composition (Wang and Ni, 2014; Zhang, 2014; Zhi et al., 2014; Zhang and Li, 2016; Jiang and Liu, 2019; Hu et al., 2024), the basic eight herbs of Soyo-san were used: *Bupleurum chinense* DC. [Apiaceae; Bupleuri Radix]*, Paeonia lactiflora* Pall [Paeoniaceae; Paeoniae Radix], *Angelica sinensis* (Oliv.) Diels [Apiaceae; Angelicae Sinensis Radix], *Wolfiporia cocos* (Schw.) Ryvarden and Gilb. [Polyporaceae; Poria] (Fungus) in all studies (100%). *Atractylodes macrocephala* Koidz. [Asteraceae; Atractylodis Macrocephalae Rhizoma] in 33 (94.3%), *Glycyrrhiza uralensis* Fisch. [Fabaceae; Glycyrrhizae Radix et Rhizoma] in 33 (94.3%), *Mentha arvensis* L. [Lamiaceae; Menthae Haplocalycis Herba] in 24 (85.7%), and *Zingiber officinale Roscoe* [Zingiberaceae; Zingiberis Rhizoma Recens] in 24 (68.6%). Furthermore, 39 additional herbs were used as adjuvants based on pattern identification or symptoms (Supplementary Table S5).

### 3.4 Soyo-san combined with antidepressants *versus* antidepressants only

#### 3.4.1 Effect on depression

**FIGURE 3:**
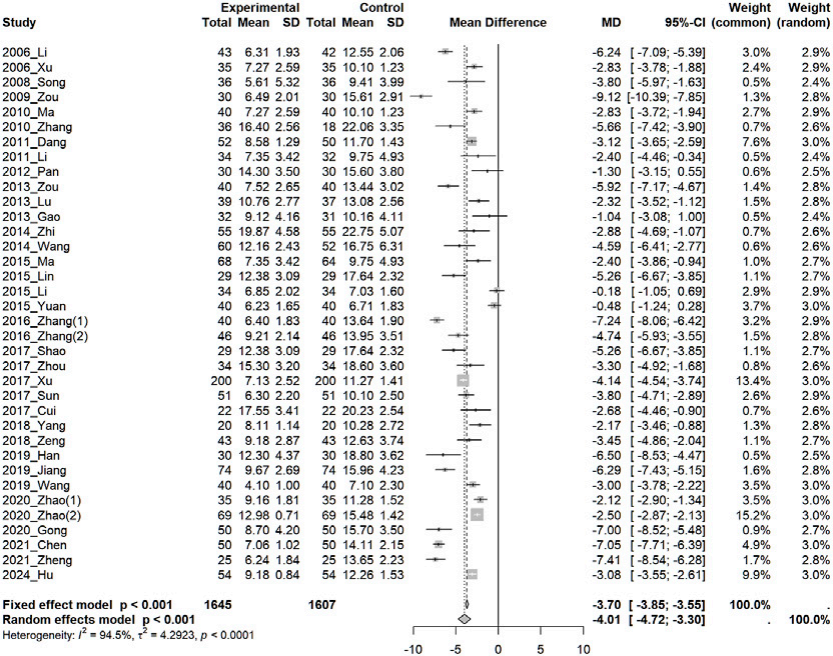
Forest plot of Hamilton Depression Scale (HAMD) CI, Confidence Interval; MD, Mean Difference; SD, Standard Deviation.

In 36 studies, HAMD scores were significantly lower in the treatment group than in the control group (MD: −4.01; 95%CI: −4.72, −3.30, *I* ^2^ = 94%) (Figure 3). Subgroup analyses were conducted based on the class of antidepressants used in the control group, treatment duration, application of the HAMD criteria for participant selection, pattern identification, and Soyo-san dosage form, and no significant differences were generally observed. Subgroup analysis was also conducted between groups with mild-to-moderate or moderate-to-severe depression as classified by the HAMD criteria used for participant selection. One study (Jiang and Liu, 2019) that adopted only moderate levels was included in the moderate-to-severe group. However, the analysis did not reveal any significant differences (Supplementary Figure S2-1). Analysis of variance (ANOVA) based on class of antidepressant used by the control group, treatment duration, application of HAMD criteria for participant selection, pattern identification, and Soyo-san dosage form also did not show significant differences. A meta-regression analysis performed using treatment duration as a moderator indicated a trend of decreasing HAMD score with longer treatment durations, although the results were not statistically significant (Supplementary Figure S3).

**FIGURE 4:**
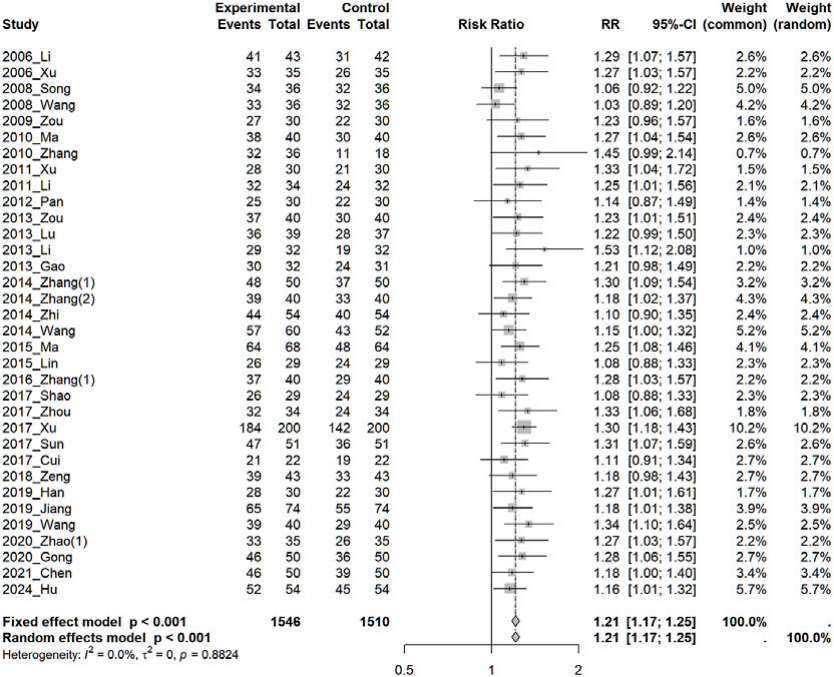
Forest plot of total effective rate (TER). RR, Risk Ratio; TER, Total Effective Rate.

In 34 studies, TER was significantly higher in the treatment group than in the control group (RR: 1.21, 95%CI: 1.17, 1.25, *I* ^2^ = 0% and OR: 3.89, 95%CI: 3.10, 4.88, *I* ^2^ = 0%) (Figure 4). Subgroup analyses were conducted based on the class of antidepressants used in the control group, treatment duration, application of the HAMD criteria for participant selection, pattern identification, and Soyo-san dosage form. Improvements were observed across all outcomes, although no significant differences were observed in the subgroup analysis. Subgroup analysis was also conducted based on the severity of depression, as classified by the HAMD criteria used for participant selection, distinguishing between the group with a severity of mild or more and moderate or more. One study (Jiang and Liu, 2019) that adopted only moderate level was included as the group of moderate or more (Supplementary Figure S2-1). This analysis showed no significant differences. ANOVA based on the class of antidepressant used for control group, treatment duration, application of HAMD criteria for participant selection, pattern identification, and SYS dosage form also did not show significant differences. Meta-regression analysis using treatment duration as a moderator showed a decreasing trend in RR with longer treatment duration, although the results were not significant (Supplementary Figure S3).

In seven studies, 5-HT levels were significantly higher in the treatment group than in the control group (MD: 105.53; 95%CI: 49.19, 161.86; *I* ^2^ = 97%) (Supplementary Figure S2-2) (Yang et al., 2018; Zeng et al., 2018; Han et al., 2019; Jiang and Liu, 2019; Wang et al., 2019; Zhao D. et al., 2020a; Zheng, 2021).

#### 3.4.2 Effect on post-stroke function

In two studies, FMA scores were significantly higher in the treatment group than in the control group. (MD: 7.92, 95%CI: 3.40, 12.45; *I* ^2^ = 0%) (Supplementary Figure S2-2) (Yang et al., 2018; Zheng, 2021).

In 8 studies, MBI scores were significantly higher in the treatment group than in the control group (MD: 14.12; 95%CI: 8.22, 20.01; *I* ^2^ = 97%). Subgroup analysis and ANOVA were conducted based on the antidepressant class used in the control group, treatment duration, application of HAMD criteria for participant selection, pattern identification, and Soyo-san dosage form. No significant differences were observed between groups (Supplementary Figure S2-1) (Li and Gao, 2006; Song and Yang, 2008; Zhang and Li, 2016; Zhang and Zhang, 2016; Cui et al., 2017; Han et al., 2019; Zhao S. et al., 2020c; Zheng, 2021).

In 3 studies, the NIHSS scores were significantly lower in the treatment group than in the control group (MD: −3.67; 95%CI: −7.29, −0.04; *I* ^2^ = 98%) (Supplementary Figure S2-2) (Cui et al., 2017; Wang et al., 2019; Zhao D. et al., 2020a).

In 7 studies, SSS scores were significantly lower in the treatment group than in the control group (MD: −4.89; 95%CI: −7.47, −2.31; *I* ^2^ = 97%). Subgroup analysis and ANOVA were conducted based on the antidepressant class used in the control group, treatment duration, application of HAMD criteria for participant selection, pattern identification, and the Soyo-san dosage form. No significant differences were observed between groups (Supplementary Figure S2-1) (Li and Gao, 2006; Song and Yang, 2008; Zou et al., 2009; 2013; Dang and Zhang, 2011; Zhang and Li, 2016; Zheng, 2021).

In 2 studies, MMSE scores were higher in the treatment group than in the control group, although the difference was not statistically significant (MD: 2.64; 95%CI: −0.07, 5.35; *I* ^2^ = 85%) (Supplementary Figure S2-2) (Song and Yang, 2008; Zhao S. et al., 2020c).

In 3 studies, PSQI scores were lower in the treatment group than in the control group, although the difference was not significant (MD: −2.70; 95% CI: −3.33, −2.06; *I* ^2^ = 50%) (Supplementary Figure S2-2) (Yang et al., 2018; Zhao D. et al., 2020a; Zheng, 2021).

#### 3.4.3 Safety

In most of the included studies, adverse events that occurred in the treatment and control groups were reported as symptom frequency. Few studies have defined treatment-emergent signs and symptoms or used them for reporting. Although quantitative synthesis for meta-analysis was not feasible due to the reporting of symptoms rather than number of individuals with adverse events, the treatment group generally exhibited fewer adverse events than the control group in all studies reporting adverse events.

#### 3.4.4 Subgroup analysis

Subgroup analyses were conducted for the HAMD, TER, MBI, and SSS, but no significant differences were observed.

### 3.5 Quality of evidence via GRADE methodology

With GRADE methodology, the quality of evidence was rated as “very low” or “low,” with no high-quality evidence available, mainly because of the high risk of bias in the RCTs included in the meta-analysis. Additionally, most outcome measures did not have an adequate number of eligible participants, leading to low precision, heterogeneity, and indirectness, which further downgraded the quality of evidence (Table 3).

**TABLE 3:** Quality assessment of evidence (GRADE).

Certainty assessment							№ of patients		Effect		Certainty	Importance
№ of studies	Study design	Risk of bias	Inconsistency	Indirectness	Imprecision	Other considerations	SYS + WM	WM	Relative (95% CI)	Absolute (95% CI)		
Depression (follow-up: range 2 weeks–12 weeks; assessed with: HAMD)												
36	Randomised trials	Serious^a^	Serious^b^	Not serious	Not serious	None	1,645	1,607	-	MD 4.01 lower (4.72 lower to 3.3 lower)	⊕⊕○○ Low^a,b^	CRITICAL
Depression (follow-up: range 2 weeks–12 weeks; assessed with: HAMD reduction rate)												
34	Randomised trials	Serious^a^	Not serious	Not serious	Not serious	None	1,428/1,546 (92.4%)	1,136/1,510 (75.2%)	RR 1.21 (1.17–1.25)	158 more per 1,000 (from 128 more to 188 more)	⊕⊕⊕○ Moderate^a^	CRITICAL
Depression (follow-up: range 4 weeks–60 days; assessed with: 5-HT (Serotonin))												
6	Randomised trials	Serious^a^	Serious^c^	Not serious	Serious^d^	None	247	247	-	MD 105.53 higher (49.19 higher to 161.86 higher)	⊕○○○ Very low^a,c,d^	CRITICAL
Cognition (follow-up: range 8 weeks–12 weeks; assessed with: MMSE)												
2	Randomised trials	Serious^a^	Serious^b^	Not serious	Serious^d^	None	105	105	-	MD 2.64 higher (0.07 lower to 5.35 higher)	⊕○○○ Very low^a,b,d^	IMPORTANT
Sleeping Condition (follow-up: range 4 weeks–8 weeks; assessed with: PSQI)												
3	Randomised trials	Serious^a^	Not serious	Not serious	Serious^d^	None	80	80	-	MD 2.7 lower (3.33 lower to 2.06 lower)	⊕⊕○○ Low^a,d^	NOT IMPORTANT
Post Stroke Recovery (follow-up: range 4 weeks–12 weeks; assessed with: MBI)												
8	Randomised trials	Serious^a^	Serious^b,e^	Not serious	Serious^d^	None	311	310	-	MD 14.12 higher (8.22 higher to 20.01 higher)	⊕○○○ Very low^a,b,d,e^	IMPORTANT
Post Stroke Recovery (follow-up: range 4 weeks–60 days; assessed with: NIHSS)												
3	Randomised trials	Serious^a^	Serious^b,f^	Not serious	Serious^d^	None	97	97	-	MD 3.67 lower (7.29 lower to 0.04 lower)	⊕○○○ Very low^a,b,d,f^	NOT IMPORTANT

CI, confidence interval; MD, mean difference; RR, risk ratio.

Explanations.

a. Most studies were figured out to be High Risk by RoB2.

b. Heterogeneity is 75% or over.

c. Studies of inconsistent MD values analysed (2018_Zeng).

d. Under 300 participants for dichotomous variables and 400 participants for continuous variables were downgraded.

e. Studies of inconsistent MD values analysed (2008_Song, 2016_Zhang (2)).

f. Studies’ CIs totally unmatched.

### 3.6 Herbal medicine report via ConPhyMP checklists

In accordance with the guideline, an assessment of the herbal medicine interventions was performed using the ConPhyMP checklists for all included studies. Among 41 included studies, 20 were classified as Type A (Li and Gao, 2006; Wang, 2008; Zou et al., 2009; 2013; Dang and Zhang, 2011; Pan and Wan, 2012; Gao, 2013; Lu and Chen, 2013; Wang and Ni, 2014; Zhang et al., 2014; Yuan, 2015; Shao et al., 2017; Zhou, 2017; Yang et al., 2018; Zeng et al., 2018; Han et al., 2019; Jiang and Liu, 2019; Wang et al., 2019; Hu et al., 2024), 21 as Type B (Xu, 2006; Song and Yang, 2008; Ma, 2010; 2015; Zhang, 2010; 2014; Li et al., 2011; Xu et al., 2011; 2017; Li, 2013; 2015; Lin et al., 2015; Zhang and Li, 2016; Zhang and Zhang, 2016; Cui et al., 2017; Sun, 2017; Gong et al., 2020; Zhao S. et al., 2020c; Zhao D. et al., 2020a; Chen et al., 2021; Zheng, 2021). As shown in Tables 4–6, the response rates were very low in general. For Checklist 1, 41.5% (17/41) of studies provided a basic description of the ingredients (Item 3), 0% (0/41) of studies reported description of the botanical drug and taxonomic authentication (Item 2) and documentation of the legal basis for collection and processing (Item 4). Furthermore, almost every items in Checklist 2a and 2b were reported zero. The detailed study-by-study analysis documents are available in *Supplementary data sheets*.

**TABLE 4:** CoPhyMP checklist 1 reports summary.

Item no.	Topic	Yes	No	Not applicable	Reporting rate (%)
1	Title and abstract	41	0	0	100
2	Description of the botanical drug and taxonomic authentication	0	41	0	0.0
3	Description of the extract and extraction process	17	24	0	41.5
4	Documentation of the legal basis for collection and processing	0	41	0	0.0
5	Description of product characteristics, in case of a finished (commercial) product	1	19	21	5.0

Reporting Rate for Item 5 calculated based on n = 20 applicable studies (41 total - 21 N/A).

**TABLE 5:** CoPhyMP checklist 2a reports summary.

Item no.	Topic	Yes	No	Not applicable	Reporting rate (%)
1	Type of extract	20	0	0	100
2–1 (a)	Preferred/main methods for extract characterisation/chemical analysis	0	20	0	0.0
2–1 (b)		0	20	0	0.0
2–1 (c)		1	11	8	8.3
2–2 (a)		0	20	0	0.0
2–2 (b)		0	20	0	0.0
3 (a)	Alternative methods for extract characterisation/chemical analysis	0	20	0	0.0
3 (b)		0	20	0	0.0
4	Use of reference standards	0	20	0	0.0
5	Comparison of different extracts/samples of the same plants	0	20	0	0.0

Reporting Rate for Item 2–1 (c) calculated based on n = 12 applicable studies (20 total - 8 N/A).

**TABLE 6:** CoPhyMP checklist 2b reports summary.

Item no.	Topic	Yes	No	Not applicable	Reporting rate (%)
1	Type of extract	21	0	0	100
2 (a)	Preferred/main methods for extract characterisation/chemical analysis	0	21	0	0.0
2 (b)		0	21	0	0.0
3 (a)	Alternative methods for extract characterisation/chemical analysis	0	21	0	0.0
3 (b)		0	21	0	0.0
4	Use of reference standards	0	21	0	0.0
5	Comparison of different extracts/samples of the same plants	0	21	0	0.0

Reporting Rate calculated against 21 Type B studies.

### 3.7 Sensitivity analysis

When models of the effect sizes of HAMD were compared, the random-effects model (MD: −4.01; 95% CI: −4.72, −3.30; *I* ^2^ = 94%) showed better improvement than the common-effects model (MD: −3.70; 95% CI: −3.85, −3.55; *I* ^2^ = 94%), with both showing significant outcomes. Outcomes other than HAMD were also similar between the random- and common-effects models.

Leave-one-out analysis revealed no significant differences in either effect size or heterogeneity. For HAMD, heterogeneity was high (*I* ^*2*^ = 94%, τ^2^ = 4.2923). Leave-one-out analyses did not identify a single study that accounted for the dispersion. In the cumulative meta-analysis based on publication year and sample size, no significant differences were found, indicating robustness of the outcomes (Supplementary Figure S5).

### 3.8 Publication bias

**FIGURE 5:**
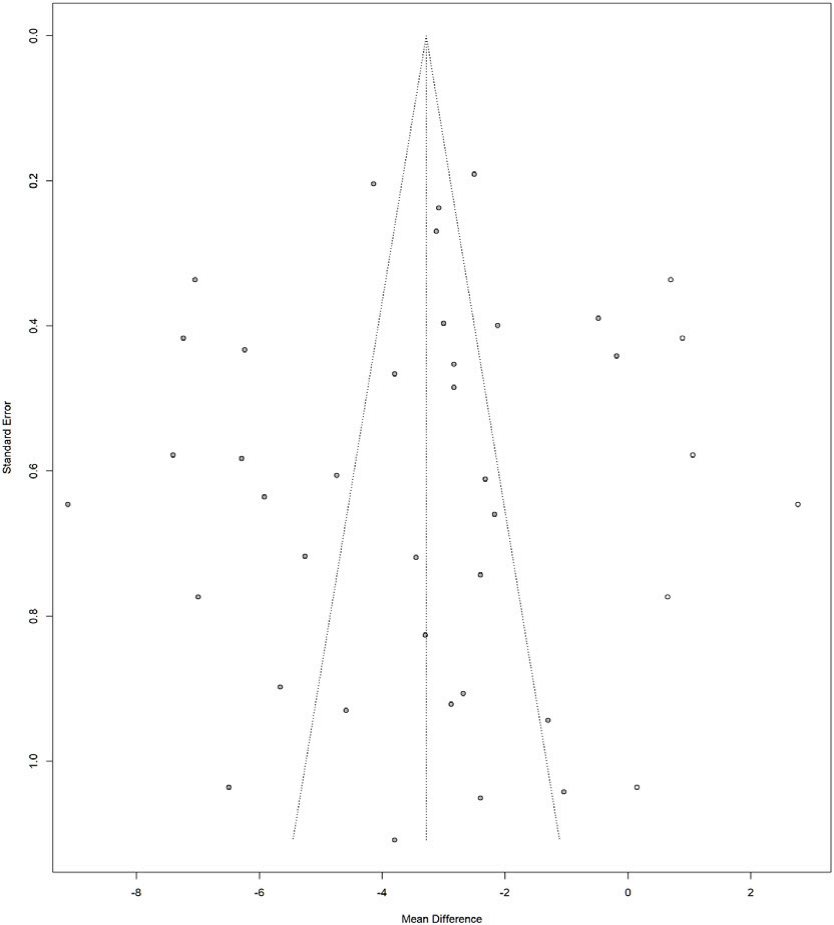
Funnel plot of Hamilton Depression Scale (HAMD).

A funnel plot was drawn for the primary outcome (HAMD) to analyze the publication bias of the included studies (Figure 5). The results of Egger’s regression, which was conducted for quantitative analysis, did not show a statistically significant or visually apparent publication bias. Similarly, no publication bias was observed for the TER (Supplementary Figure S4).

## 4 Discussion

### 4.1 Summary of findings

We systematically selected 41 RCTs that involved the combination of Soyo-san with conventional antidepressants to treat PSD and analyzed the effectiveness and safety using various methods. Combination treatment with Soyo-san showed significant effects on measures such as HAMD, TER, serotonin level, MBI, NIHSS, and SSS. The treatment group generally exhibited fewer adverse effects than the control group in all included studies. Subgroup analyses of HAMD, TER, MBI, and SSS outcomes did not reveal any significant differences. Meta-regression using treatment duration as the moderator variable for HAMD and TER did not yield significant findings. The risk of bias analysis indicated ‘some concerns’ of high risk of bias in the included studies. The quality of evidence for the outcome measures, assessed using GRADE, ranged from very low to moderate, with no high-level evidence. No publication bias was observed for HAMD and TER based on funnel plots and Egger’s regression.

### 4.2 Debate: diagnostic criteria

PSD is typically diagnosed based on the DSM-5 as mentioned in the introduction. However, the RCTs included in this study employed various diagnostic criteria, such as the 2001 Chinese Classification and Diagnostic Criteria of Disorders, in addition to or instead of the DSM-5. Few studies diagnosed depression without describing the criteria used or based on arbitrarily set symptoms and signs, indicating an overall lack of clarity and consistency in diagnosis. Furthermore, the Soyo-san dosage and frequency for concomitant therapy varied across studies, and few studies lacked detailed explanations regarding its administration. Because the patients had PSD, the interventions applied to both the treatment and control groups were not consistently described, which compromised the consistency of the patient selection criteria across studies, leading to limitations in the interpretation of the results.

### 4.3 Debate: comparison with prior research

In a systematic review and meta-analysis of Soyo-san for PSD in 2022 (Wang et al., 2022), the diagnostic criteria were not described, and assessment of the diagnostic content in the selected studies was not conducted. The 2022 study reported significant improvements in HAMD, TER, SSS, 5-HT level, and BI as outcome measures that were similar to those of the present study: HAMD (MD: −4.56; 95%CI: −6.39, −2.74; *I* ^2^ = 95% vs. MD −4.01, 95%CI −4.72, −3.30; *I* ^2^ = 94%: random-effect), TER (RR: 1.21; 95%CI: 1.13, 1.29; *I* ^2^ = 8% vs. RR: 1.21; 95%CI: 1.17, 1.25; *I* ^2^ = 0%: common-effect), SSS (MD: −5.73; 95%CI: −9.86, −1.79; *I* ^2^ = 92% vs. MD: −4.89; 95%CI: −7.47, −2.31; *I* ^2^ = 97%: random-effect), BI (MD: 15.47; 95%CI: 12.89, 18.04; *I* ^2^ = 13% vs. MD: 10.17; 95%CI: 9.58, 10.75; *I* ^2^ = 97%: common-effect), and 5-HT (SMD: 5.11; 95%CI: 3.11, 7.12; *I* ^2^ = 13% vs. SMD: 1.55; 95%CI: 1.33, 1.77; *I* ^2^ = 97%: random-effect). However, the number of included studies for each outcome measure in the present study was significantly larger than that in the 2022 study: HAMD (36 vs. 11), TER (34 vs. 10), SSS (7 vs. 2), BI (8 vs. 2), and 5-HT (7 vs. 2), thus providing more precise effect estimates and helpful information for clinical judgment. Furthermore, the present study employed additional outcome measures such as NIHSS, FMA, MMSE, and PSQI, allowing for consideration of the complexity of PSD symptoms. Finally, subgroup analyses were conducted by applying various criteria to investigate whether the study outcomes differed among specific subgroups or remained robust.

### 4.4 Debate: potential alternative therapeutic option for PSD

The combination of Soyo-san and antidepressants demonstrated significant clinical effects beyond alleviating depression to include post-stroke functional recovery. In particular, the minimum clinically significant difference for the primary outcome, HAMD, has not been previously reported. The National Institute for Health and Care Excellence of the United Kingdom recommends a 3-point difference between the treatment and placebo groups to be considered clinically significant. Other studies suggest a 2-point difference indicates a clinical correlation (Kirsch et al., 2008; Montgomery and Möller, 2009). These reports compared treatment with placebo, which is not equivalent to this study. However, our result (MD: −4.01; 95%CI: −4.72, −3.30; *I* ^2^ = 94%) can be considered clinically significant because it exceeds a 3-point difference. In TEAM, Soyo-san is prescribed to ‘Soothe the liver and relieve depression’ (疏肝解鬱), ‘fortify the spleen and nourish blood’ (健脾養血) on Liver qi stagnation with blood deficiency (肝鬱血虛), spleen failing in transportation (脾失健運) state provoked by emotional disorder (情志不暢). The antidepressant effects of Soyo-san have been observed in preclinical experiments demonstrating its ability to alleviate stress-related anxiety. *Bupleurum chinense* DC. [Apiaceae; Bupleuri Radix], a key ingredient in Soyo-san, has antidepressant properties, particularly when paired with *Paeonia lactiflora* Pall [Paeoniaceae; Paeoniae Radix] (Kwon et al., 2010; Zhang et al., 2020). *Angelica sinensis* (Oliv.) Diels [Apiaceae; Angelicae Sinensis Radix] helps regulate circulation and exhibits antidepressant effects. *Wolfiporia cocos* (Schw.) Ryvarden and Gilb. [Polyporaceae; Poria] (Fungus) enhances antidepressant function by regulating neurotransmission and decreasing inflammation in the brain (Gong et al., 2019; Huang et al., 2020). *Glycyrrhiza uralensis* Fisch. [Fabaceae; Glycyrrhizae Radix et Rhizoma] has neuroprotective effects, while *Mentha arvensis* L [Lamiaceae; Menthae Haplocalycis Herba], *Zingiber officinale* Roscoe [Zingiberaceae; Zingiberis Rhizoma Recens], and *Atractylodes macrocephala* Koidz. [Asteraceae; Atractylodis Macrocephalae Rhizoma] have antioxidant and anti-inflammatory properties (Koşar et al., 2004; Van Breemen et al., 2011; Kao et al., 2014; Hoang et al., 2016).

Patients with PSD experience various sequelae including motor impairment, cognitive impairment, and sleep disorders. Depression exacerbates their condition and reduces treatment compliance. Stroke survivors benefit from prompt and active rehabilitation to restore their ability to perform activities of daily living. PSD hinders recovery, worsens sequelae, and increases medication burden. Soyo-san may provide a comprehensive treatment that addresses this complex situation. Recent systematic reviews on SSRIs in post-stroke recovery reported improvements not only in preventing and treating depression but also in reducing anxiety and dependence in daily life and improving motor and cognitive function (Kalbouneh et al., 2022). However, SSRIs are associated with a higher risk of seizures than placebo. In this context, Soyo-san, when used in combination with SSRIs, demonstrated superior effects in improving various post-stroke sequelae, contributing to the recovery of daily life abilities. This suggests the potential of combination therapy to alleviate the burden of polypharmacy. Furthermore, the safety profile of combination therapy appears favorable, with fewer reported adverse effects than antidepressants alone. However, the effects of combined administration on seizures and increased risk of hemorrhage require further investigation.

The antidepressant effects observed with Soyo-san in this review are consistent with findings for other traditional herbal medicines. For instance, systematic reviews of Banxia-houpo-tang for depression (Kim et al., 2023) and Sihogayonggolmoryeo-tang for post stroke depression (Kwon et al., 2019) have also reported beneficial effects on depressive symptoms, although similarly limited by the methodological quality of primary studies. In particular, study of Sihogayonggolmoryeo-tang analysed effect on Barthel Index, same as our study. Situating Soyo-san within this broader context suggests a class of herbal formulas may target neuropsychiatric symptoms through shared anti-inflammatory and neuro-regulatory pathways, though direct comparative studies are lacking and urgently needed.

### 4.5 Debate: clinical indications

Regardless of the class of co-administered antidepressant, duration of administration, pattern identification, and dosage form, significant improvement in symptoms were observed based on the HAMD. Additionally, although not statistically significant, there was a trend toward better symptom improvement with administration >4 weeks and a preference for the order of dosage forms as pills, powders, and decoctions. Further research is needed to elucidate the potential differences in responses based on these factors.

Exploring the impact of severity classification, the study compared subgroups based on whether the HAMD criterion was applied during participant selection. Although not statistically significant, better outcomes were observed in the group for which the HAMD criterion was applied. Further subgroup analyses based on severity (mild, moderate) showed no significant differences; however, a tendency for better outcomes was observed in the moderate subgroup. This suggests that the co-administration of Soyo-san has a significant antidepressant effect, irrespective of severity, making it a potential treatment for various levels of PSD.

### 4.6 Strengths and limitations

This study conducted a comprehensive search without language restrictions and employed various analysis methods, including subgroup analysis, meta-regression analysis, and GRADE, methods not implemented in the previous study, to evaluate the outcomes. Moreover, the study utilized a diverse set of outcome measures to confirm the positive effects of adjunctive therapy with Soyo-san, addressing not only PSD, but also overall post-stroke symptoms. The included studies exhibited unclear or inconsistent diagnostic criteria, limited geographic diversity, high risk of bias, and generally low levels of evidence. However, this study employed methods such as subgroup analysis, sensitivity analysis, and assessment of publication bias, which confirmed that the results remained stable and robust.

In geographic diversity, all included studies were conducted in China. As local healthcare practices and cultural frameworks may influence trial conduct and outcome selection, the findings may not generalize to other countries.

For heterogeneity shown from results, methodological quality (e.g., risk of bias in allocation concealment), variability in herbal medicine formulation (e.g., composition, dose), differences in stroke characteristics (e.g., severity, subtype, time since onset), diversity in co-interventions (e.g., type of antidepressants, rehabilitation, acupuncture) are considered to be associated. Exploratory analyses suggested few trends (e.g., longer treatment duration and decoction form were associated with greater reductions in HAMD), but residual heterogeneity remained high. Given the substantial heterogeneity, the pooled effect estimate should be interpreted with caution. We present it alongside sensitivity analyses and exploratory subgroup/meta-regression findings, but emphasise that the true effect may vary across contexts.

For The Total Effective Rate (TER), while widely used in Chinese clinical trials, lacks international validation and standardization. Its cultural specificity and composite nature limit comparability to globally accepted outcome measures, reducing generalizability of our findings. Functional outcomes such as Barthel Index and NIHSS were reported in ≤3 trials, providing insufficient evidence for firm conclusions. By contrast, HAMD was assessed in nearly all trials, albeit with high heterogeneity. While all included outcome measures showed statistically significant results, no validated Minimum Clinically Important Differences (MCIDs) were available to confirm their clinical relevance to PSD.

Adverse events were inconsistently reported, often without standardized severity grading. This underreporting prevents a balanced assessment of risks. Future trials should follow CONSORT-harms reporting to ensure adequate evaluation of safety.

The ConPhyMP assessment results highlight limitations regarding the heterogeneity and low reproducibility of herbal medicines in this review. Over half of the studies failed to report the basic description of their plant material, botanical authentication, and all of the included studies failed to provide chemical information. This deficiency reporting on plant origin, chemical identity, product quality information is associated to heterogeneity and low reproducibility in clinical outcomes. Our findings strongly underscore the need for future clinical trials of Soyosan to adhere to the ConPhyMP guidelines to ensure that systematic reviews and meta-analyses can draw reliable conclusions.

### 4.7 Implications for future research

This study attempted subgroup analyses to explore the factors influencing the effects of Soyo-san combination therapy on PSD; however, no significant differences were observed. Additional research is needed to investigate potential differences based on covariates such as co-administered medication, treatment duration, baseline severity, the presence of pattern identification, and dosage form.

Limitations of the strictness of the diagnostic criteria and design of concomitant therapies for post-stroke management other than combined therapy were identified. This suggests participant selection may have been suboptimal, leading to potential distortions in outcome interpretation, including subgroup analyses. Hence, future RCTs should apply diagnostic criteria according to the DSM-5 and explicitly state stroke management for both patient and control groups.

Given the observed effects of Soyo-san beyond alleviating PSD, more proactive RCTs are needed to elucidate its effects on general post-stroke symptoms. Moreover, investigating whether Soyo-san positively addresses the issue of polypharmacy in patients with stroke is essential. Although this study confirmed that Soyo-san, when combined with antidepressants, shows superior effects in PSD, reduces the burden of adverse effects, and may resolve various symptoms related to polypharmacy, further research is required to define the specific conditions necessary to achieve these goals.

## 5 Conclusion

This study demonstrated significant improvements in various scales, including HAMD, TER, 5-HT, MBI, NIHSS, and SSS, following combined therapy with Soyo-san. Fewer adverse effects were reported in the combined treatment group. However, these promising findings must be interpreted with caution, as the majority of the included studies were assessed as having a high risk of bias and a low quality of evidence. Therefore, Soyo-san appears to be an effective and safe therapeutic alternative for managing depressive symptoms in post-stroke patients, particularly in cases where post-stroke rehabilitation treatment adherence is compromised. Future research should prioritize clear criteria for participants, treatments, and comparisons, focusing on minimizing antidepressant side effects and polypharmacy while achieving effective therapeutic outcomes.

## Data Availability

The original contributions presented in the study are included in the article/Supplementary Material, further inquiries can be directed to the corresponding authors.
