# Supplementary material for: Effects of modified Soyo-san (Xiao-yao-san, Shoyo-san) combined with antidepressants on post-stroke depression and functional recovery: a systematic review and meta-analysis
Source: Front Pharmacol. 2026 Jan 2;16:1651831. doi: 10.3389/fphar.2025.1651831 (PMC12808369; doi:10.3389/fphar.2025.1651831)
Supplement: Supplementary file 1 [file Supplementaryfile1.pdf]

Supplementary Document.

Index:

Supplementary Table 1. Search strategy

Supplementary Table 2. Excluded reports by eligibility assessment

Supplementary Table 3. Details of TER

Supplementary Table 4. Adverse effects

Supplementary Table 5. Daily dose of medicinal herbs in of SYS

Supplementary Table 6. RoB2 Domain-wise Summary

Supplementary Figure 1. RoB2 Traffic-light Plot

Supplementary Figure 2-1. Subgroup analysis

Supplementary Figure 2-2. Outcomes other than HAMD, TER(OR)

Supplementary Figure 3. Meta regression analysis

Supplementary Figure 4. Publication bias : TER(RR)

Supplementary Figure 5. Sensitivity analysis : HAMD

Supplementary Table 1. Search strategy.

DataBase : PubMed (2024.10.10.)		
**No.**	**Search Query**	**Items found**
#1	"depressive disorder"[MeSH Terms] OR "depression"[MeSH Terms] OR depressive OR depression	659,560
#2	"stroke"[MeSH Terms] OR stroke	471,868
#3	jia-wei-xiao-yao-san OR xiao-yao-san OR xiao-yao powder OR xiao-yao wan OR xiao-yao OR kamishoyosan OR Shoyo OR TJ-24 OR Soyosan OR Soyo-san OR Soyo OR Gami-soyosan OR Gamisoyo-san	1,482
#4	#1 AND #2 AND #3	6

DataBase : EmBase (2024.10.10.)		
**No.**	**Search Query**	**Items found**
#1	“depression”/exp OR “depression”	1,001,018
#2	Depressive	224,602
#3	“cerebrovascular disease”/exp OR “cerebrovascular disease”	994,220
#4	“stroke patient”/exp OR “stroke patient”	51,502
#5	Stroke	631,288

#6	"jia-wei-xiao-yao-san" OR "xiao-yao-san" OR "xiao-yao powder" OR "xiao-yao wan" OR "kamishoyosan" OR "TJ-24" OR "Soyosan" OR "Soyo-san" OR "Gami-soyosan" OR "Gamisoyo-san" OR "xiao-yao" OR "Shoyo" OR "Soyo"	534
#7	(#1 OR #2) AND (#3 OR #4 OR #5) AND #6	6

**DataBase : CENTRAL (2024.10.10.)**		
**No.**	**Search Query**	**Items found**
#1	MeSH descriptor: [depressive disorder] explode all trees	16,827
#2	MeSH descriptor: [depression] explode all trees	18,685
#3	depressive OR depression	117,984
#4	MeSH descriptor: [stroke] explode all trees	17,885
#5	stroke	89,889
#6	jia-wei-xiao-yao-san OR xiao-yao-san OR xiao-yao powder OR xiao-yao wan OR kamishoyosan OR TJ-24 OR Soyosan OR Soyo-san OR Gami-soyosan OR Gamisoyo-san OR xiao-yao OR Shoyo OR Soyo	61
#7	(#1 OR #2 OR #3) AND (#4 OR #5) AND #6 in Trials	0

DataBase : CINAHL (2024.10.10.)		
**No.**	**Search Query**	**Items found**
#1	MH depressive disorder	86,737
#2	MH depression	569,884
#3	TX depressive OR TX depression	3,340,358
#4	MH stroke	316,011
#5	TX stroke	2,232,068
#6	TX jia-wei-xiao-yao-san OR TX xiao-yao-san OR TX xiao-yao powder OR TX xiao-yao wan OR TX kamishoyosan OR TX TJ-24 OR TX Soyosan OR TX Soyo-san OR TX Gami-soyosan OR TX Gamisoyo-san OR TX xiao-yao OR TX Shoyo OR TX Soyo	4,628
#7	(S1 OR S2 OR S3) AND (S4 OR S5) AND S6	163

DataBase : CNKI (2024.10.10.)		
**No.**	**Search Query**	**Items found**
#1	(SU='中风'+'脑卒中') AND (SU='抑郁'+'忧郁') AND (SU='加味逍遙散'+'逍遙散'+'逍遙')	116

DataBase : CiNii (2024.10.10.)

No.	Search Query	Items found
#1	(脳卒中 OR 中風) AND (加味逍遙散 OR 逍遙散 OR 逍遙) AND (抑うつ OR うつ病)	0
#2	(脳卒中 OR 中風) AND (加味逍遙散 OR 逍遙散 OR 逍遙) AND 抑鬱	0

DataBase : KCI (2024.10.10.)		
**No.**	**Search Query**	**Items found**
#1	뇌졸중 AND 우울 AND 가미소요산	0
#2	중풍 AND 우울 AND 가미소요산	0
#3	뇌졸중 AND 우울 AND 소요산	0
#4	뇌졸중 AND 우울 AND 소요산	0

Supplementary Table 2. Excluded reports by eligibility assessment

Author	Subject	Reason for exclusion
2021_Ren	Clinical observation of Modified Xiao-yao-san in Treating Gan-Yu-Pi-Xu-type Post-stroke Depression	Head-to-head study
2010_Xiao	Modified Xiaoyaosan treatment of post-stroke depression(qi stagnation) in clinical study	Head-to-head study
2017_Liu	Clinical observation on the treatment of post-stroke depression by Jia wei xiaoyao san	Head-to-head study
2020_Li	The effect of treatment with Jiaweixiaoyao granules on the improvement of depression and quality of life after stroke	Head-to-head study
2019_Yin	Discussion on the therapeutic effect of Jiawei danzhi xiaoyaosan in the treatment of post-stroke depression	Head-to-head study
2009_Yang	Treatment of liver depression and qi obstruction-type post-stroke depression with Jiawei Xiaoyaosan combined with fluoxetine in 30 cases	Head-to-head study
2011_Chen	Observations on 28 cases of post-stroke depression treated with the addition and subtraction of Danzhi Xiaoyaosan	Head-to-head study
2004_Xiao	Treatment of post-stroke depression with 68 cases of Xiaoyaosan	Head-to-head study
2007_Wang	Clinical study on the treatment of post-stroke depression with the treatment of Xiaoyao antidepressant powder and its effect on plasma 5-HT content	Head-to-head study
2007_Wang	Clinical study on the treatment of post-stroke depression by Xiaoyao antidepressant powder	Head-to-head study
2018_Min	Study on the treatment of depressive disorders after cerebral infarction by Xiaoyao Pill	Head-to-head study
2016_Meng	Clinical analysis of the treatment of post-stroke depression by Jiawei Danzhi xiaoyaosan	Head-to-head study
2019_Wang	Analysis of the effect of treating 68 patients with post-stroke depression by using Xaioyaosan	Head-to-head study
2010_Bai	Meta-analysis of the efficacy of Xiaoyaosan in the treatment of post-stroke depression	Meta analysis study
2016_Zheng	Clinical study on the treatment of post-stroke depression syndrome by Shengxinjieluo method combined with Xiaoyaosan	Study’s focused on other main treatment
2014_Zuo	A clinical study on the treatment of post-stroke depression by Jiawei danzhi xiaoyaosan	Poor randomization
2016_Wu	The clinical observation of jiajianxiaoyaosan to cure the post-stroke depression(Ganyipixu)	Poor randomization
2014_Shang	Clinical Observation on 30 Cases of Post-stroke Depression Treated with Traditional Chinese Medicine Diagnosis and Treatment	Various HMs used
2006_Qiao	Treatment of 98 cases of post-stroke affective disorder by Jiawei Xiaoyaosan	Comparison group designed poorly
2004_Geng	Early application of Xiaoyao Pill for post-stroke depressive disorder	Comparison group designed poorly
2009_Zeng	Clinical research of the method of soothing the liver on post-stroke depression	Poor randomization
2013_Gao	60 cases of post-stroke depression treated with danzhi xiaoyaosan combined with escitalopram tablets	No mention of randomization
2009_Xu	Clinical observation on the treatment of post-stroke depression with the addition and subtraction of Xiaoyaosan	No mention of randomization
2016_Huang	Comparison of the efficacy of Black Xiaoyaosan in the treatment of post-stroke depression in left hemiparesis and right hemiparesis	No mention of randomization
2016_Liu	Treatment of post-stroke depression by bu yang huan wu tang combined with xiaoyaosan	HM used other than SYS

2015_Zhang	Clinical observation on the treatment of post-stroke depression by bu yang huan wu tang combined with xiaoyaosan	HM used other than SYS
2005_Zhang	Clinical study on the treatment of post-stroke depression with the method of benefiting the kidney and sparing the liver	SYS as comparison intervention
2018_Wang	Discussion on the clinical efficacy and some mechanisms of electro-needle combined with xiaoyaosan on post-stroke depression	Electroacupuncture as comparison intervention, not conventional medicine
2021_Yuan	Clinical treatment of depressed patients after stroke	Review article
2021_Liu	An overview of liver-based treatment of post-stroke depression	Review article
2006_Zhang	Clinical observation of fluoxetine combined with Chinese and Tibetan medicines in the treatment of geriatric depression in highland areas	Research for different disease
2010_Cai	The protective mechanisms experimental study of Chang Yu Xiaoyaosan in post-stroke depression mouse	Mouse experiment
2016_Kim	Clinical observation on the treatment of post-stroke depression with Changpu Yujin Decoction combined with the addition and subtraction of Xaioyaosan	HM used other than SYS
2018_Yao	Clinical efficacy observation on the treatment of post-stroke depression by Xiaoyaosan with acupuncture	Acupuncture used only for treatment group
2005_Tai	Treatment of post-stroke depression in the elderly with acupuncture and Xiaoyaosan 50 cases	Acupuncture used only for treatment group
2018_Zhang	Clinical effect of Danzhi Xiaoyao powder combined with acupuncture in the treatment of patients with post stroke depression	Acupuncture used only for treatment group
2016_Liu	Clinical Observation on the Treatment of Post Stroke Depression With Acupuncture and Xiaoyaosan	Acupuncture used only for treatment group
2021_Wu	Clinical observation on the treatment of post-stroke depression by eye acupuncture with Xiaoyaosan	Acupuncture used only for treatment group
2014_Zhang	Danzhi Xiaoyao Powder Combined with acupuncture therapy in the treatment of depression after stroke(qi stagnation fire type) clinical study	Acupuncture used only for treatment group
2016_Xue	Clinical efficacy of acupuncture-medicine concurrent intervention for post-stroke depression (liver-depression and spleen-deficiency type) in the community	Acupuncture used only for treatment group
2010_Xu	Traditional treatment of stroke Depression(liver Qi stagnation) in Clinical Research	HM versus HM
2012_Jiang	The clinical observation of Jiaweixiaoyaosan to cure the disease depression after stroke(Ganyupixuxing)	HM versus HM
2019_Liu	Evaluation of therapeutic effects on post - stroke depression treated with xiaoyao san and auricular acupuncture	Ear acupuncture used only for treatment group
2016_Wu	Xiaoyaosan Joint after Auricular Acupressure Treatment Depression of Stroke Randomized Controlled Study	Ear acupuncture used only for treatment group
2019_Tseung	Effect of Danzhi Xiaoyaosan combined with acupuncture therapy on the expression of BDNF and 5-HT in patients with	Acupuncture used only for treatment

	post-stroke depression	group
2014_Wan	The clinical therapeutic effects of Zhuyu Xiaoyao Tang and acupuncture Baihui on Qi-stagnationi and blood stasis type of Post Cerebral Infarction Depression(PCID)	Acupuncture used only for treatment group
2023_Yang	Mechanism of Xiaoyao Powder in the Treatment of Post-stroke Depression Based on Network Pharmacology	Pharmacological mechanism research
2024_Guo	Peng Tao's experience in treating post-stroke depression from the perspective of liver, spleen, and stomach and case study analysis	Case report
2023_Wang	Efficacy of the treatment of post-stroke depression with the addition of the flavoring of Xiaoyu San and its effect on long-term quality of life	No mention of randomization
2024_Erdene	Analysis of the effect of combined psychotherapy with Mongolian drug Zadi-5 and Prosperity San in the treatment of post-stroke depression	Used other type treatments with HM
2024_Huang	Thirty cases of "brain and intestines treated together" acupuncture therapy combined with dande jasmino prophylactic dispersal for the treatment of qi depression and fire type mild-moderate post-stroke depression	Used other type treatments with HM
2024_Wang	Clinical study on the treatment of patients with mild post-stroke depression by Danghaji Yuanshan combined with five elements music therapy	Used other type treatments with HM

Supplementary Table 3. Details of TER.

no	study	TER criteria	b1	n1	b2	n2
1	2006_Li	Cured: symptoms completely disappeared and emotions returned to normal; >75%<br>Significantly Effective: main symptoms improved significantly and emotions were basically stable; >50%<br>Effective: some symptoms improved and emotions improved slightly; ≥25%<br>Ineffective: symptoms and emotions did not improve; <25%	95.35(41/43)	43	73.81(31/42)	42
2	2006_Xu	Cured: symptoms completely disappeared and emotions returned to normal;<br>Significantly Effective: main symptoms improved significantly and emotions were basically stable;<br>Effective: some symptoms improved and emotions improved slightly;<br>Ineffective: symptoms and emotions did not improve<br>"Diagnostic and Efficacy Criteria for Chinese Medicine", 1994	94.30(33/35)	35	74.29(26/35)	35
3	2008_Song①	Based on the reduction rate of the HAMD score.<br>Cured: ≥75% reduction rate. Significantly Effective: ≥50% reduction rate.<br>Effective: ≥25% reduction rate. Ineffective: score reduction rate <25%.	94.4(34/36)	36	88.9(30/34)	36
4	2008_Wang	The reduction rate of HAMD score (%) = (total score before treatment - total score after treatment)/total score before treatment*100%.<br>Cured: disappearance of psychiatric symptoms with a reduction rate of ≥75%;<br>Significantly Effective: basic disappearance of psychiatric symptoms with a reduction rate of 50%-70%;<br>Effective: reduction of psychiatric symptoms with a reduction rate of 25%-49%;<br>Ineffective: no change or aggravation of the condition with a reduction rate of <25%.	91.7(33/36)	36	88.9(32/36)	36
5	2009_Zou	The efficacy was assessed by the reduction rate of the scores:<br>Cured: reduction rate ≥ 75%, Significantly Effective: reduction rate < 75%,<br>Effective: reduction rate < 50%, Ineffective: reduction rate < 25%.	93.33(27/30)	30	73.33(22/30)	30
6	2010_Ma	Cured: symptoms completely disappeared and emotions returned to normal;<br>Significantly Effective: main symptoms improved significantly and emotions were basically stable;<br>Effective: some symptoms improved and emotions improved slightly;<br>Ineffective: symptoms and emotions did not improve<br>"Diagnostic and Efficacy Criteria for Chinese Medicine", 1994	95(38/40)	40	75(30/40)	40
7	2010_Zhang	The efficacy was assessed by the reduction rate of the HAMD scores:<br>Cured: ≥75% reduction rate. Significantly Effective: ≥50% reduction rate.<br>Effective: ≥25% reduction rate. Ineffective: score reduction rate <25%.	88.89(32/36)	36	61.11(11/18)	18
8	2011_Xu	The HAMD score was used as a reference.	93.33(28/30)	30	70.00(21/30)	30

		Cured: complete disappearance of symptoms, score reduction rate ≥ 75%;<br>Significantly Effective: basic disappearance of symptoms, score reduction rate of 50%-74%;<br>Effective: symptom reduction or partial disappearance, score reduction rate of 25%-49%;<br>Ineffective: no improvement of symptoms or no significant improvement, score reduction rate < 25%.				
10	2011_Li	The efficacy was assessed by the reduction rate of the HAMD scores:<br>Cured: reduction rate ≥ 75%; Significantly Effective: reduction rate of 50%-74%;<br>Effective: reduction rate of 25%-49%; Ineffective: reduction rate < 25%.	94.12(32/34)	34	75.00(24/32)	32
11	2012_Pan	The HAMD score was measured before treatment, at the end of the second week of treatment, at the end of the fourth week of treatment, and at the end of the eighth week of treatment, and the HAMD reduction rate = [(pre-treatment score - post-treatment score) / pre-treatment score]*100%.<br>The reduction rate of ≥75% was considered as cure, 50- 75% was considered as significantly effective, 25-50% was considered as effective, and <25% was considered as ineffective.	83.3(25/30)	30	73.3(22/30)	30
12	2013_Zou	The clinical efficacy was evaluated before treatment, at the second week and at the end of the fourth week after treatment. The rate of score reduction was used as the criteria:<br>≥75% was considered as cured, 50%-74% was considered as significantly effective, 25%-49% was considered as effective, and less than 24% was considered as ineffective.<br>Total effective rate=(cured+significantly effective+effective)/total number of cases*100%. The degree of improvement in depressive symptoms was assessed by the HAMD scale.	92.5(37/40)	40	75.0(30/40)	40
13	2013_Lu	Cured: symptoms completely disappeared and emotions returned to normal;<br>Significantly Effective: main symptoms improved significantly and emotions were basically stable;<br>Effective: some symptoms improved and emotions improved slightly;<br>Ineffective: symptoms and emotions did not improve<br>"Diagnostic and Efficacy Criteria for Chinese Medicine", 1994<br>The depression efficacy standard is the reduction rate: (total score before treatment-total score after treatment)/total score before treatment*100 %.<br>cured≥75%; significantly effective≥50%; effective≥25%; ineffective<25 %.	92.31(36/39)	39	75.68(28/37)	37
14	2013_Li	The rate is defined as the reduction rate of HAMD score.<br>Cured: symptoms completely disappeared and emotions returned to normal; ≥75%<br>Significantly Effective: main symptoms improved significantly and emotions were basically stable; 50~74%<br>Effective: some symptoms improved and emotions improved slightly; 35~49%<br>Ineffective: symptoms and emotions did not improve; <25%	90.6(29/32)	32	59.4(19/32)	32
15	2013_Gao	The rate is defined as the reduction rate of HAMD score.<br>Cured: symptoms completely disappeared and emotions returned to normal; >75%	93.75(30/32)	32	77.42(24/31)	31

		Significantly Effective: main symptoms improved significantly and emotions were basically stable; >50%<br>Effective: some symptoms improved and emotions improved slightly; ≥25%<br>Ineffective: symptoms and emotions did not improve; <25%<br>"Diagnostic and Efficacy Criteria for Chinese Medicine", 2nd Edition				
16	2014_Zhang(1)	The rate is defined as the reduction rate of HAMD score.<br>Cured: symptoms completely disappeared and emotions returned to normal; ≥75%<br>Significantly Effective: main symptoms improved significantly and emotions were basically stable; 50~74%<br>Effective: some symptoms improved and emotions improved slightly; 25~49%<br>Ineffective: symptoms and emotions did not improve; <25%<br>Total effective rate=(cured+significantly effective+effective)/total number of cases*100%.	96(48/50)	50	74(37/50)	50
17	2014_Zhang(2)	The rate is defined as the reduction rate of HAMD score.<br>≥75% was considered as cured, 50%-74% was considered as significantly effective, 25%-49% was considered as effective, and less than 24% was considered as ineffective.	97.5(39/40)	40	82.5(33/40)	40
18	2014_Zhi	HAMD before and after 8 weeks of treatment, and the efficacy was evaluated according to the reduction rate, which was = (total score before treatment - total score after treatment) / total score before treatment*100%.<br>Cured ≥75%; Significantly effective 50~74%; Effective 25~49%; Ineffective <25%.	81.48(44/54)	54	74.07(40/54)	54
19	2014_Wang	The rate is defined as the reduction rate of HAMD score.<br>Total effective rate=(cured+significantly effective+effective)/total number of cases*100%.<br>Cured ≥75%; Significantly effective 50~74%; Effective 25~49%; Ineffective <25%.	95.0(57/60)	60	82.7(43/52)	52
20	2015_Ma	The rate is defined as the reduction rate of HAMD score.<br>Cured >75%; Significantly effective 50~75%; Effective 25~50%; Ineffective <25%.	94.12(64/68)	68	75.00(48/64)	64
21	2015_Lin	The rate is defined as the reduction rate of HAMD score.<br>(total score before treatment - total score after treatment) / total score before treatment*100%.<br>Cured ≥75%; Significantly effective≥50; Effective≥25; Ineffective <25%.	89.7(26/29)	29	82.7(24/29)	29
24	2016_Zhang(1)	The rate is defined as the reduction rate of HAMD score.<br>Cured>75%; Significantly effective>50%; Effective≥25%; Ineffective<25%.	92.5(37/40)	40	72.5(29/40)	40
26	2017_Shao	The rate is defined as the reduction rate of HAMD score.<br>Cured≥75%; Significantly effective≥50%; Effective≥25%; Ineffective<25%.	89.7(26/29)	29	82.8(24/29)	29
27	2017_Zhou	The rate is defined as the reduction rate of HAMD score.<br>Cured ≥75%; Significantly effective 50~74%; Effective 25~49%; Ineffective <25%.	94.1(32/34)	34	70.6(24/34)	34
28	2017_Xu	The rate is defined based on the HAMD score;<br>Cured: <8, symptoms completely disappeared and emotions returned to normal;	92.0(184/200)	200	71.0(142/200)	200

		Significantly Effective: 8~10, main symptoms improved significantly and emotions were basically stable;<br>Effective: 10~18, some symptoms improved and emotions improved slightly;<br>Ineffective: No difference in score, symptoms and emotions did not improve<br>Total effective rate=(cured+significantly effective+effective)/total number of cases*100%.<br>"Diagnostic and Efficacy Criteria for Chinese Medicine", 1994				
29	2017_Sun	The rate is defined based on the HAMD score;<br>Significantly Effective: <8, main symptoms improved significantly and emotions were basically stable;<br>Effective: >20, some symptoms improved and emotions improved slightly;<br>Ineffective: >35, symptoms and emotions did not improve	92.16(47/51)	51	70.59(36/51)	51
30	2017_Cui	The rate is defined as the reduction rate of HAMD score.<br>Cured ≥75%; 75%>Significantly effective≥50; 50%>Effective≥25; Ineffective <25%.	95.5(21/22)	22	86.4(19/22)	22
32	2018_Zeng	The rate is defined as the reduction rate of HAMD score(%) = (total score before treatment - total score after treatment)/total score before treatment*100%.<br>Total effective rate=(cured+significantly effective+effective)/total number of cases*100%.<br>Cured ≥75%; Significantly effective 50~74%; Effective 25~49%; Ineffective <25%.	90.69(39/43)	43	76.74(33/43)	43
33	2019_Han	The rate is defined as the reduction rate of HAMD score.<br>Cured >75%; 75%≥Significantly effective≥50; 50%>Effective≥25; Ineffective <25%.	93.33(28/30)	30	73.33(22/30)	30
34	2019_Jiang	The rate is defined as the reduction rate of HAMD score.<br>Cured: symptoms completely disappeared; ≥75%<br>Significantly Effective: symptoms improved significantly; 51~74%<br>Effective: emotions improved back to stable state; 30~50%<br>Ineffective: clinical symptoms were improved nothing; <30%	87.84(65/74)	74	74.32(55/74)	74
35	2019_Wang	The reduction rate of HAMD≥75% was considered as cure, 50- 75% was considered as significantly effective, 25-50% was considered as effective, and <25% was considered as ineffective.<br>Total effective rate=(cured+significantly effective+effective)/total number of cases*100%.	97.5(39/40)	40	72.5(29/40)	40
36	2020_Zhao(1)	The rate is defined as the reduction rate of HAMD score(%) = (total score before treatment - total score after treatment)/total score before treatment*100%.<br>Cured: HAMD score<7, ≥75% symptoms completely disappeared;<br>Significantly Effective: <75%, ≥50%, symptoms improved significantly;<br>Effective: <50%, ≥25%, symptoms mildly improved;<br>Ineffective: <25%, symptoms did not improve	94.29(33/35)	35	74.29(26/35)	35
38	2020_Gong	Cured: symptoms completely disappeared and emotions returned to normal;<br>Significantly Effective: main symptoms improved significantly and emotions were basically stable;	92.0(46/50)	50	72.0(36/50)	50

		Effective: some symptoms improved and emotions improved slightly;<br>Ineffective: symptoms and emotions did not improve				
39	2021_Chen	Significantly effective : symptoms completely cured or significantly improved without adverse effects.<br>Effective : symptoms improved with only mild adverse effects then healed naturally.<br>Ineffective : symptoms not improved or aggravated with significant adverse effects.<br>Total effective rate=(significantly effective+effective)/total number of cases*100%.	92.00(46/50)	50	78.00(39/50)	50
40	2024_Hu	The rate is defined as the reduction rate of HAMD score.<br>Cured: symptoms completely disappeared; >75%<br>Significantly Effective: symptoms improved significantly; 50~75%<br>Effective: emotions improved back to stable state; 25~49%<br>Ineffective: clinical symptoms were improved nothing; <25%	96.30(52/54)	54	83.33(45/54)	54

Supplementary Table 4. Adverse effects.

no	study	Adverse effect
1	2006_Li	(A) : Headache(2), Nausea&Anorexia(2)<br>(B) : Headache(2), Nausea&Anorexia(3)
4	2008_Wang	(A) : Anorexia(5), Nausea(4), Abdomen distension(3)<br>(B) : Anorexia(5), Nausea(3), Abdomen distension(4)
7	2010_Zhang	(A) : Indigestion(8; 22.22%), Autonomic dysfunction(10; 27.78%)<br>(B) : Indigestion(10; 55.56%), Autonomic dysfinction(9; 50.00%)
8	2011_Xu	(A) : Upper abdomen discomfort(3)<br>(B) : Dry mouth, Anorexia, Nausea, Insomnia, Fatigue, Headache(6)
10	2011_Li	(A) : None<br>(B) : Insomnia(1)
12	2013_Zou	(A) : Narcolepsy(1), Insomnia(1), Dry mouth(1)<br>(B) : Nausea(1), Vomiting(1), Dizziness(2), Sweating(1), Anorexia(1), Papule(1)
17	2014_Zhang(2)	TESS<br>(A) : Nausea&Vomiting(1), Headache&Fatigue(1), Diarrhea(1), Transient palpitation(1)<br>(B) : Nausea&Vomiting(2), Headache&Fatigue(2), Insomnia(4), Diarrhea(2), Constipation(1), Transient palpitation(1)
18	2014_Zhi	(A) : Transient anxiety&Insomnia(1), Dry mouth(2), Constipation(1)<br>(B) : Transient anxiety&Insomnia(1), Dry mouth(2), Constipation(1)
19	2014_Wang	(A) : None<br>(B) : Insomnia(2)
20	2015_Ma	(A) : None<br>(B) : Insomnia(2)
21	2015_Lin	(A) : Dizziness(2), Fatigue(2), Insomnia(2)<br>(B) : Dizziness(3), Fatigue(1), Insomnia(1)
22	2015_Li	(A) : Indigestion(3), Dry mouth(1), Narcolepsy(1)<br>(B) : Indigestion(2), Dry mouth(1), Dysuria(1)
23	2015_Yuan	TESS<br>(A) : Headache(4), Dizziness(3), Narcolepsy(3), Blurred vision(1), Nausea&Vomiting(5), Fatigue(4), Constipation(4), Bradycardia(4), Weight gain(3), Orthostatic hypotension(4), At least one adverse event(5)<br>(B) : Headache(4), Dizziness(3), Narcolepsy(4), Blurred vision(1), Nausea&Vomiting(4), Fatigue(3), Constipation(5), Bradycardia(3), Weight gain(3), Orthostatic hypotension(4), At least one adverse event(6)

24	2016_Zhang(1)	(A) : Nausea(2), Anorexia(2)<br>(B) : Nausea(3), Anorexia(3)
27	2017_Zhou	(A) : Bradycardia(1), Insomnia(1), Dry mouth(1) : 8.8%<br>(B) : Bradycardia(2), Insomnia(1) : 8.8%
32	2018_Zeng	(A) : None<br>(B) : None
33	2019_Han	(A) : Nausea&Vomiting(1), Fatigue(1)<br>(B) : Nausea&Vomiting(4), Fatigue(3), Anxiety&Tremor(1)
36	2020_Zhao(1)	(A) : Dizziness(1)<br>(B) : Abdomen distension(1), Constipation(1), Dizziness(1), Insomnia(1), Dry mouth(1)
39	2021_Chen	(A) : Mental disorders(1)<br>(B) : Heart burn&Anxiety(2), Insomnia(2), Mental disorders(4)
40	2021_Zheng	(A) : Insomnia(1), Fatigue(1)<br>(B) : Diarrhea(1), Insomnia(2), Constipation(1), Fatigue(2)
41	2024_Hu	(A) : Vomiting(1), Diarrhea(1), Headache(2), Nausea(1)<br>(B) : Diarrhea(1), Headache(1)

Supplementary Table 5. Daily dose of each medicinal herbs in SYS

(1) Dose of herbs in SYS for each RCTs.

	**A1(柴胡)**	**A2(芍藥)**	**A3(當歸)**	**A4(白朮)**	**A5(茯苓)**	**A6(甘草)**	**A7(薄荷)**	**A8(生薑)**	**A9(牡丹皮)**	**A10(梔子)**	**A11(天南星)**	**A12(石菖蒲)**	**A13(川芎)**	**A14(香附子)**	**A15(蒼朮)**	**Substance (Preparation)**
2006_Li	10	10	10	10	10	5	3	5								decoction
2006_Xu	6	6	6	6	6	6	6	6	6	6						granule
2008_Song	15	15	15	15	30		6	6	15	15	12	30	30			decoction
2008_Wang	NR	NR	NR	NR	NR	NR	NR		NR	NR						pill
2009_Zou	NR	NR	NR	NR	NR	NR	NR	NR								pill
2010_Ma (a)	15	15	15	15	30		6	6	15	15	12	30	30			decoction
2010_Zhang	10	15	15		15	6						10	10	6		decoction
2011_Xu (b)	12	15	15	10	15	5	10	3								decoction
2011_Dang (c)	15	15	15	15	10	6								10		decoction
2011_Li (d)	15	15	15	15	12	6	6					15	12			decoction
2012_Pan (e)	14	10	10	8	15	6	6					10	9	6		decoction
2013_Zou	NR	NR	NR	NR	NR	NR	NR	NR								granule

2013_Lu（f）	15	15	15	15	15	6	6	15								decoction
2013_Li	15	15	15	15	15	6	6	15								decoction
2013_Gao	NR	NR	NR	NR	NR	NR	NR	NR	NR	NR						granule
2014_Zhang(1)	15	15	15	15	15	6	6	15								decoction
2014_Zhang(2)																pill
2014_Zhi																pill
2014_Wang																pill
2015_Ma（g）	15	15	15	15	12	6	6				12	15	12			decoction
2015_Lin（h）	12	12	10		12	6			10	10						decoction
2015_Li（i）	15	15	20	15	15	6	15	15	15	15						decoction
2015_Yuan	15	10	10	10	10	5	5	5								decoction
2016_Zhang(1)																granule
2016_Zhang(2)（j）	10	15	10	10	10	5	3		10	10	10	15		10		decoction
2017_Shao	10	10	10	10	10	10			10	10						decoction
2017_Zhou	15	15	15	15	15	6	6	15								granule
2017_Xu（k）	15	10	10	10	10	5	5	10	10	15		10				granule

2017_Sun	6	15	15	15	15	6	6	15								decoction
2017_Cui (l)	10	12	12	12	10	10	6	6	6							decoction
2018_Yang	30	30	9	15	15	6		3(pieces)								decoction
2018_Zeng	NR	NR	NR	NR	NR	NR	NR	NR								pill
2019_Han (m)	15	15	10	10	16	6	6	3								decoction
2019_Jiang																granule
2019_Wang	6	10	10	10	15	6	3									decoction
2020_Zhao(1) (n)	12	12	12	12	12	6	12			10			10	10	10	decoction
2020_Zhao(2) (o)	12	12	12	12	12	8	6	6	12	8				10		decoction
2020_Gong (p)	15	10	10	10	15	6	6	3				12	10			decoction
2021_Chen (q)	15	15	20	15	15	10	10	10	15	15			15			decoction
2021_Zheng	3	3	3	3	10	1.5	3	5	1.5	1.5						decoction
2024_Hu																pill

* **NR** : None reported; **A1**：柴胡 (Chaihu) *Bupleurum chinense DC.* [Apiaceae; Bupleuri Radix]; **A2**：芍藥 (Shaoyao) *Paeonia lactiflora* Pall. [Paeoniaceae; Paeoniae Radix]; **A3**：當歸 (Danggui) *Angelica sinensis* (Oliv.) Diels [Apiaceae; Angelicae Sinensis Radix]; **A4**：白朮 (Baizhu) *Atractylodes macrocephala* Koidz. [Asteraceae; Atractylodis Macrocephalae Rhizoma]; **A5**：茯苓 (Fuling) *Wolfiporia cocos* (Schw.) Ryvarden & Gilb. [Polyporaceae; Poria] (Fungus); **A6**：甘草 (Gancao) *Glycyrrhiza uralensis* Fisch. [Fabaceae; Glycyrrhizae Radix et Rhizoma]; **A7**：薄荷 (Bohe) *Mentha arvensis* L. [Lamiaceae; Menthae Haplocalycis Herba]; **A8**：

生薑 (Shengjiang) *Zingiber officinale* Roscoe [Zingiberaceae; Zingiberis Rhizoma Recens]; **A9** : 牡丹皮 (Mudanpi) *Paeonia suffruticosa* Andr. [Paeoniaceae; Moutan Cortex]; **A10** : 栀子 (Zhizi) *Gardenia jasminoides* J.Ellis [Rubiaceae; Gardeniae Fructus]; **A11** : 天南星 (Tiannanxing) *Arisaema erubescens* (Wall.) Schott [Araceae; Arisaematis Rhizoma]; **A12** : 石菖蒲 (Shichangpu) *Acorus tatarinowii* Schott [Acoraceae; Acori Tatarinowii Rhizoma]; **A13** : 川芎 (Chuanxiong) *Ligusticum chuanxiong* Hort. [Apiaceae; Chuanxiong Rhizoma]; **A14** : 香附子 (Xiangfuzi) *Cyperus rotundus* L. [Cyperaceae; Cyperi Rhizoma]; **A15** : 蒼朮 (Cangzhu) *Atractylodes lancea* (Thunb.) DC. [Asteraceae; Atractylodis Rhizoma]

[Additional herbs in SYS of each study]

(a) 黄芩 *Scutellaria baicalensis* Georgi [Lamiaceae; Scutellariae Radix] 10; 半夏 *Pinellia ternata* (Thunb.) Makino [Araceae; Pinelliae Rhizoma] 10; 小麦 *Triticum aestivum* L. [Poaceae; Tritici Fructus Levis] 20; 百合 *Lilium lancifolium* Thunb. [Liliaceae; Lilii Bulbus] 20; 水蛭 *Hirudo nipponia* Whitman [Hirudinidae; Hirudo] 3; 太子参 *Pseudostellaria heterophylla* (Miq.) Pax [Caryophyllaceae; Pseudostellariae Radix] 30; 白芷 *Angelica dahurica* (Fisch. ex Hoffm.) Benth. & Hook.f. ex Franch. & Sav. [Apiaceae; Angelicae Dahuricae Radix] 6; 枳壳 *Citrus × aurantium* L. [Rutaceae; Aurantii Fructus] 6

(b) 酸枣仁 *Ziziphus jujuba* var. spinosa (Bunge) Hu ex H.F.Chow [Rhamnaceae; Ziziphi Spinosae Semen] 15; 合欢皮 *Albizia julibrissin* Durazz. [Fabaceae; Albiziae Cortex] 30; 郁金 *Curcuma wenyujin* Y.H.Chen & C.Ling [Zingiberaceae; Curcumae Radix] 15;

(c) 郁金 *Curcuma wenyujin* Y.H.Chen & C.Ling [Zingiberaceae; Curcumae Radix] 10; 枳壳 *Citrus × aurantium* L. [Rutaceae; Aurantii Fructus] 10; 合欢皮 *Albizia julibrissin* Durazz. [Fabaceae; Albiziae Cortex] 10; 夜交藤 *Reynoutria multiflora* (Thunb.) Moldenke [Polygonaceae; Polygoni Multiflori Caulis] 20; 酸枣仁 *Ziziphus jujuba* var. spinosa (Bunge) Hu ex H.F.Chow [Rhamnaceae; Ziziphi Spinosae Semen] 20

(d) 天南星 *Arisaema erubescens* (Wall.) Schott [Araceae; Arisaematis Rhizoma] 12; 桃仁 *Prunus persica* (L.) Batsch [Rosaceae; Persicae Semen] 10; 红花 *Carthamus tinctorius* L. [Asteraceae; Carthami Flos] 10

(e) 琥珀 Succinum [Mineral/Resin; Succinum] 6

(f) 郁金 *Curcuma wenyujin* Y.H.Chen & C.Ling [Zingiberaceae; Curcumae Radix]10

(g) 桃仁 *Prunus persica* (L.) Batsch [Rosaceae; Persicae Semen] 10; 红花 *Carthamus tinctorius* L. [Asteraceae; Carthami Flos] 10

(h) 枳壳 *Citrus × aurantium* L. [Rutaceae; Aurantii Fructus] 10; 合欢皮 *Albizia julibrissin* Durazz. [Fabaceae; Albiziae Cortex] 12; 竹茹 *Bambusa tuldoides* Munro [Poaceae; Bambusae Caulis in Taeniam] 10

(i) 钩藤 *Uncaria rhynchophylla* (Miq.) Miq. ex Havil. [Rubiaceae; Uncariae Ramulus cum Unco] 15; 郁金 *Curcuma wenyujin* Y.H.Chen & C.Ling [Zingiberaceae; Curcumae Radix] 15

(j) 郁金 *Curcuma wenyujin* Y.H.Chen & C.Ling [Zingiberaceae; Curcumae Radix] 10; 枳实 *Citrus × aurantium* L. [Rutaceae; Aurantii Fructus Immaturus] 10

(k) 远志 *Polygala tenuifolia* Willd. [Polygalaceae; Polygalae Radix] 10

(l) 党参 *Codonopsis pilosula* (Franch.) Nannf. [Campanulaceae; Codonopsis Radix] 6

(m) 郁金 *Curcuma wenyujin* Y.H.Chen & C.Ling [Zingiberaceae; Curcumae Radix] 10

(n) 神曲 Massa Medicata Fermentata [Fermented Preparation; Massa Medicata Fermentata] 10

(o) 红花 *Carthamus tinctorius* L. [Asteraceae; Carthami Flos] 10; 郁金 *Curcuma wenyujin* Y.H.Chen & C.Ling [Zingiberaceae; Curcumae Radix] 10

(p) 郁金 *Curcuma wenyujin* Y.H.Chen & C.Ling [Zingiberaceae; Curcumae Radix] 15

(q) 郁金 *Curcuma wenyujin* Y.H.Chen & C.Ling [Zingiberaceae; Curcumae Radix] 15

(2) Standards for modification of SYS in each RCTs

no	study	Modifying standards and herbs
3	2008_Song	Phlegm and turbidity inside：青礞石 Lapis Chloriti [Mineral; Lapis Chloriti]; 鲜竹沥 *Phyllostachys nuda* McClure [Poaceae; Bambusae Succus]<br>Heart and spleen deficiency：黄芪 *Astragalus mongholicus* Bunge [Fabaceae; Astragali Radix]; 太子参 *Pseudostellaria heterophylla* (Miq.) Pax [Caryophyllaceae; Pseudostellariae Radix]<br>Heart and kidney disconnection :：黄连 *Coptis chinensis* Franch. [Ranunculaceae; Coptidis Rhizoma]; 肉桂 *Cinnamomum cassia* (L.) J.Presl [Lauraceae; Cinnamomi Cortex]<br>Dry stools：大黄 *Rheum palmatum* L. [Polygonaceae; Rhei Radix et Rhizoma]<br>Frightened and uneasy：龙齿 Fossilia Ossis Mastodi [Fossil; Fossilia Ossis Mastodi]; 牡蛎 *Magallana gigas* (Thunb.) [Ostreidae; Ostreae Concha]
6	2010_Ma	Phlegm and turbidity inside：青礞石 Lapis Chloriti [Mineral; Lapis Chloriti]; 鲜竹沥 *Phyllostachys nuda* McClure [Poaceae; Bambusae Succus]<br>Heart and spleen deficiency：黄芪 *Astragalus mongholicus* Bunge [Fabaceae; Astragali Radix]; 太子参 *Pseudostellaria heterophylla* (Miq.) Pax [Caryophyllaceae; Pseudostellariae Radix]<br>Heart and kidney disconnection :：黄连 *Coptis chinensis* Franch. [Ranunculaceae; Coptidis Rhizoma]; 肉桂 *Cinnamomum cassia* (L.) J.Presl [Lauraceae; Cinnamomi Cortex]<br>Dry stools：大黄 *Rheum palmatum* L. [Polygonaceae; Rhei Radix et Rhizoma]<br>Frightened and uneasy：龙齿 Fossilia Ossis Mastodi [Fossil; Fossilia Ossis Mastodi]; 牡蛎 *Magallana gigas* (Thunb.) [Ostreidae; Ostreae Concha]
10	2011_Li	Phlegm and dampness in abundance：半夏 *Pinellia ternata* (Thunb.) Makino [Araceae; Pinelliae Rhizoma] 12g; 苍术 *Atractylodes lancea* (Thunb.) DC. [Asteraceae; Atractylodis Rhizoma] 12g<br>Anxiety and irritability：龙齿 Fossilia Ossis Mastodi [Fossil; Fossilia Ossis Mastodi] 15g; 牡蛎 *Magallana gigas* (Thunb.) [Ostreidae; Ostreae Concha] 30g<br>Sleepless：夜交藤 *Reynoutria multiflora* (Thunb.) Moldenke [Polygonaceae; Polygoni Multiflori Caulis] 30g; 酸枣仁 *Ziziphus jujuba* var. spinosa (Bunge) Hu ex H.F.Chow [Rhamnaceae; Ziziphi Spinosae Semen] 15g<br>Dry stools：大黄 *Rheum palmatum* L. [Polygonaceae; Rhei Radix et Rhizoma] 6g
13	2013_Lu	Qi stagnation turns into fire; irritability, dry mouth and tongue, constipation：栀子 *Gardenia jasminoides* J.Ellis [Rubiaceae; Gardeniae Fructus] 10g; 大黄 *Rheum palmatum* L. [Polygonaceae; Rhei Radix et Rhizoma] 10g<br>Prolonged physical deficiency, deficiency of qi and blood; fatigue, pale complexion, lazy speech: 黄芪 *Astragalus mongholicus* Bunge [Fabaceae; Astragali Radix] 15g; 山药 *Dioscorea polystachya* Turcz. [Dioscoreaceae; Dioscoreae Rhizoma] 30g<br>Deficiency of the liver and kidney; weakness in the lower back and knees, tinnitus, drained urine, and dry eyes.: 枸杞子 *Lycium barbarum* L. [Solanaceae; Lycii Fructus] 15g; 山茱萸 *Cornus officinalis* Siebold & Zucc. [Cornaceae; Corni Fructus] 10g<br>Liver stagnation, spleen deficiency, phlegm and dampness blocking the orifices; indifference, stagnation and silence, generalized malignant phlegm, salivation：广陈皮 *Citrus reticulata* 'Chachi' [Rutaceae; Citri Reticulatae Pericarpium] 10g; 半夏 *Pinellia ternata* (Thunb.) Makino [Araceae; Pinelliae Rhizoma] 10g<br>Blood stasis; limbs pain：藿香 *Agastache rugosa* (Fisch. & C.A.Mey.) Kuntze [Lamiaceae; Agastachis Herba] 10g; 没药 *Commiphora myrrha* (Nees) Engl. [Burseraceae; Myrrha] 10g<br>Sleeping difficulties：酸枣仁 *Ziziphus jujuba* var. spinosa (Bunge) Hu ex H.F.Chow [Rhamnaceae; Ziziphi Spinosae Semen] 20g; 柏子仁 *Platycladus orientalis* (L.) Franco [Cupressaceae; Platycladi Semen] 20g<br>Irritable：靈磁石 Magnetitum [Mineral; Magnetitum] 30g; 钩藤 *Uncaria rhynchophylla* (Miq.) Miq. ex Havil. [Rubiaceae; Uncariae Ramulus cum Unco] 20g
14	2013_Li	Liver Qi stagnation, hypochondriacal swelling and stuffiness：郁金 *Curcuma wenyujin* Y.H.Chen & C.Ling [Zingiberaceae; Curcumae Radix]; 川芎 *Ligusticum chuanxiong*

		Hort. [Apiaceae; Chuanxiong Rhizoma]; 香附 *Cyperus rotundus* L. [Cyperaceae; Cyperi Rhizoma]<br>Liver stagnation turns into fire, bitter mouth, constipation: 牡丹皮 *Paeonia × suffruticosa* Andrews [Paeoniaceae; Moutan Cortex]; 栀子 *Gardenia jasminoides* J.Ellis [Rubiaceae; Gardeniae Fructus]<br>Blood stasis and tingling pain : 丹参 *Salvia miltiorrhiza* Bunge [Lamiaceae; Salviae Miltiorrhizae Radix et Rhizoma]; 桃仁 *Prunus persica* (L.) Batsch [Rosaceae; Persicae Semen]<br>Heart and mind deficiency, Dizziness, Insomnia : 百合 *Lilium lancifolium* Thunb. [Liliaceae; Lilii Bulbus]; 远志 *Polygala tenuifolia* Willd. [Polygalaceae; Polygalae Radix]; 龙骨 Fossilia Ossis Mastodi [Fossil; Fossilia Ossis Mastodi]; 牡蛎 *Magallana gigas* (Thunb.) [Ostreidae; Ostreae Concha]<br>Weakness of the spleen and anorexia : 党参 *Codonopsis pilosula* (Franch.) Nannf. [Campanulaceae; Codonopsis Radix] 黄芪 *Astragalus mongholicus* Bunge [Fabaceae; Astragali Radix] 龙眼肉 *Dimocarpus longan* Lour. [Sapindaceae; Longan Arillus]
29	2017_Sun	Liver Qi stagnation, hypochondriacal swelling and stuffiness : 郁金 *Curcuma wenyujin* Y.H.Chen & C.Ling [Zingiberaceae; Curcumae Radix]; 川芎 *Ligusticum chuanxiong* Hort. [Apiaceae; Chuanxiong Rhizoma]; 香附 *Cyperus rotundus* L. [Cyperaceae; Cyperi Rhizoma]<br>Liver stagnation turns into fire, bitter mouth, constipation: 牡丹皮 *Paeonia × suffruticosa* Andrews [Paeoniaceae; Moutan Cortex]; 栀子 *Gardenia jasminoides* J.Ellis [Rubiaceae; Gardeniae Fructus]<br>Blood stasis and tingling pain : 丹参 *Salvia miltiorrhiza* Bunge [Lamiaceae; Salviae Miltiorrhizae Radix et Rhizoma]; 桃仁 *Prunus persica* (L.) Batsch [Rosaceae; Persicae Semen]<br>Heart and mind deficiency, Dizziness, Insomnia : 百合 *Lilium lancifolium* Thunb. [Liliaceae; Lilii Bulbus]; 远志 *Polygala tenuifolia* Willd. [Polygalaceae; Polygalae Radix]; 龙骨 Fossilia Ossis Mastodi [Fossil; Fossilia Ossis Mastodi]; 牡蛎 *Magallana gigas* (Thunb.) [Ostreidae; Ostreae Concha]<br>Weakness of the spleen and anorexia : 党参 *Codonopsis pilosula* (Franch.) Nannf. [Campanulaceae; Codonopsis Radix] 黄芪 *Astragalus mongholicus* Bunge [Fabaceae; Astragali Radix] 龙眼肉 *Dimocarpus longan* Lour. [Sapindaceae; Longan Arillus]
33	2019_Han	Frequent urination and night sweats : 熟地黄 *Rehmannia glutinosa* (Gaertn.) DC. [Orobanchaceae; Rehmanniae Radix Praeparata] 10g; 山茱萸 *Cornus officinalis* Siebold & Zucc. [Cornaceae; Corni Fructus] 10g<br>Dizziness and discomfort in the head, adverse speech : 石菖蒲 *Acorus tatarinowii* Schott [Acoraceae; Acori Tatarinowii Rhizoma] 6g; 远志 *Polygala tenuifolia* Willd. [Polygalaceae; Polygalae Radix] 6g<br>Abdominal distension, belching : 麦芽 *Hordeum vulgare* L. [Poaceae; Hordei Fructus Germinatus] 30g; 六神曲 Massa Medicata Fermentata [Fermented Preparation; Massa Medicata Fermentata] 30g; 鸡内金 *Gallus gallus* (Linnaeus, 1758) [Phasianidae; Galli Gigeriae Endothelium Corneum] 10g
35	2019_Wang	Accompanied by insomnia : 远志 *Polygala tenuifolia* Willd. [Polygalaceae; Polygalae Radix] 10g; 茯神 *Wolfiporia cocos* (Schwein.) Ryvarden & Gilb. [Polyporaceae; Poria cum Radix Pino] 30g<br>Liver Stagnation and Blood Stasis : 丹参 *Salvia miltiorrhiza* Bunge [Lamiaceae; Salviae Miltiorrhizae Radix et Rhizoma] 30g; 郁金 *Curcuma wenyujin* Y.H.Chen & C.Ling [Zingiberaceae; Curcumae Radix] 10g

Supplementary Table 6. RoB2 Domain-wise Summary

Unique ID	Reviewer	Study ID	Reference	Experimental	Comparator	Outcome	Result	Aim	Effect of adhering to intervention?	Weight	Randomization process	Comment for randomization process	Deviations from intended interventions	Comment for deviations from intended interventions	Missing outcome data	Comment for missing outcome data	Measurement of the outcome	Comment for measurement of the outcome	Selection of the reported result	Comment for selection of the reported result	Overall Bias	Comment for over all bias
1		2006_Ying						assignment to intervention (the 'intention-to-treat' effect)	NA	1	Some concerns		Low		Low		High		Some concerns		High	
2		2006_Xu						assignment to intervention (the 'intention-to-treat' effect)	NA	1	Some concerns		Low		Low		High		Some concerns		High	
3		2008_Song						assignment to intervention (the 'intention-to-treat' effect)	NA	1	Some concerns		Low		Low		High		Some concerns		High	

4	2008_Wang	assignment to intervention (the 'intention-to-treat' effect)	NA	1	Some concerns	Low	Low	High	Some concerns	High
5	2009_Zou	assignment to intervention (the 'intention-to-treat' effect)	NA	1	Some concerns	Low	Low	High	Some concerns	High
6	2010_Ma	assignment to intervention (the 'intention-to-treat' effect)	NA	1	Some concerns	Low	Low	High	Some concerns	High
7	2010_Zhang	assignment to intervention (the 'intention-to-treat' effect)	NA	1	Some concerns	Low	Low	High	Some concerns	High
8	2011_Seo	assignment to intervention (the 'intentio	NA	1	Some concerns	Low	Low	High	Some concerns	High

		n-to-treat' effect)								
9	2011_Dang	assignment to intervention (the 'intention-to-treat' effect)	NA	1	Some concerns	Low	Low	High	Some concerns	High
10	2011_Li	assignment to intervention (the 'intention-to-treat' effect)	NA	1	Some concerns	Low	Low	High	Some concerns	High
11	2012_Pan	assignment to intervention (the 'intention-to-treat' effect)	NA	1	Some concerns	Low	Low	High	Some concerns	High
12	2013_Zou	assignment to intervention (the 'intention-to-treat' effect)	NA	1	Some concerns	Low	Low	High	Some concerns	High

13	2013_Lu		assignment to intervention (the 'intention-to-treat' effect)	NA	1	Some concerns	Low	Low	High	Some concerns	High
14	2013_Li		assignment to intervention (the 'intention-to-treat' effect)	NA	1	Some concerns	Low	Low	High	Some concerns	High
15	2013_Gao		assignment to intervention (the 'intention-to-treat' effect)	NA	1	Some concerns	Low	Low	High	Some concerns	High
16	2014_Zhang(1)		assignment to intervention (the 'intention-to-treat' effect)	NA	1	Some concerns	Low	Low	High	Some concerns	High
17	2014_Zhang(2)		assignment to intervention (the 'intentio	NA	1	Some concerns	Low	Low	High	Some concerns	High

			n-to-treat' effect)								
18	2014_Zhi		assignment to intervention (the 'intention-to-treat' effect)	NA	1	Low	Low	Low	Low	Some concerns	Some concerns
19	2014_Wang		assignment to intervention (the 'intention-to-treat' effect)	NA	1	Some concerns	Low	Low	High	Some concerns	High
20	2015_Ma		assignment to intervention (the 'intention-to-treat' effect)	NA	1	Some concerns	Low	Low	High	Some concerns	High
21	2015_Lin		assignment to intervention (the 'intention-to-treat' effect)	NA	1	Some concerns	Low	Low	High	Some concerns	High

22	2015_Li	assignment to intervention (the 'intention-to-treat' effect)	NA	1	Some concerns	Low	Low	High	Some concerns	High
23	2015_Yuan	assignment to intervention (the 'intention-to-treat' effect)	NA	1	Some concerns	Low	Low	High	Some concerns	High
24	2016_Zhang(1)	assignment to intervention (the 'intention-to-treat' effect)	NA	1	Some concerns	Low	Low	High	Some concerns	High
25	2016_Zhang(2)	assignment to intervention (the 'intention-to-treat' effect)	NA	1	Some concerns	Low	Low	High	Some concerns	High
26	2017_Shao	assignment to intervention (the 'intentio	NA	1	Some concerns	Low	Low	High	Some concerns	High

			n-to-treat' effect)							
27	2017_Zhou		assignment to intervention (the 'intention-to-treat' effect)	NA	1	Low	Low	Low	Low	Some concerns	Some concerns
28	2017_Xu		assignment to intervention (the 'intention-to-treat' effect)	NA	1	Some concerns	Low	Low	High	Some concerns	High
29	2017_Sun		assignment to intervention (the 'intention-to-treat' effect)	NA	1	Some concerns	Low	Low	High	Some concerns	High
30	2017_Cui		assignment to intervention (the 'intention-to-treat' effect)	NA	1	Some concerns	Low	Low	High	Some concerns	High

31	2018_Yang	assignment to intervention (the 'intention-to-treat' effect)	NA	1	Some concerns	Low	Low	High	Some concerns	High
32	2018_Zeng	assignment to intervention (the 'intention-to-treat' effect)	NA	1	Some concerns	Low	Low	High	Some concerns	High
33	2019_Han	assignment to intervention (the 'intention-to-treat' effect)	NA	1	Some concerns	Low	Low	High	Some concerns	High
34	2019_Jiang	assignment to intervention (the 'intention-to-treat' effect)	NA	1	Some concerns	Low	Low	High	Some concerns	High
35	2019_Wang	assignment to intervention (the 'intentio	NA	1	Some concerns	Low	Low	High	Some concerns	High

		n-to-treat' effect)								
36	2020_Zhao(1)	assignment to intervention (the 'intention-to-treat' effect)	NA	1	Some concerns	Low	Low	High	Some concerns	High
37	2020_Zhao(2)	assignment to intervention (the 'intention-to-treat' effect)	NA	1	Some concerns	Low	Low	High	Some concerns	High
38	2020_Gong	assignment to intervention (the 'intention-to-treat' effect)	NA	1	Some concerns	Low	Low	High	Some concerns	High
39	2021_Chen	assignment to intervention (the 'intention-to-treat' effect)	NA	1	Some concerns	Low	Low	High	Some concerns	High

40	2021_Zheng		assignment to intervention (the 'intention-to-treat' effect)	NA	1	Some concerns	Low	Low	High	Some concerns	High
41	2024_Hu		assignment to intervention (the 'intention-to-treat' effect)	NA	1	Some concerns	Low	Low	High	Some concerns	High

Supplementary Figure 1. RoB2 Traffic-light Plot

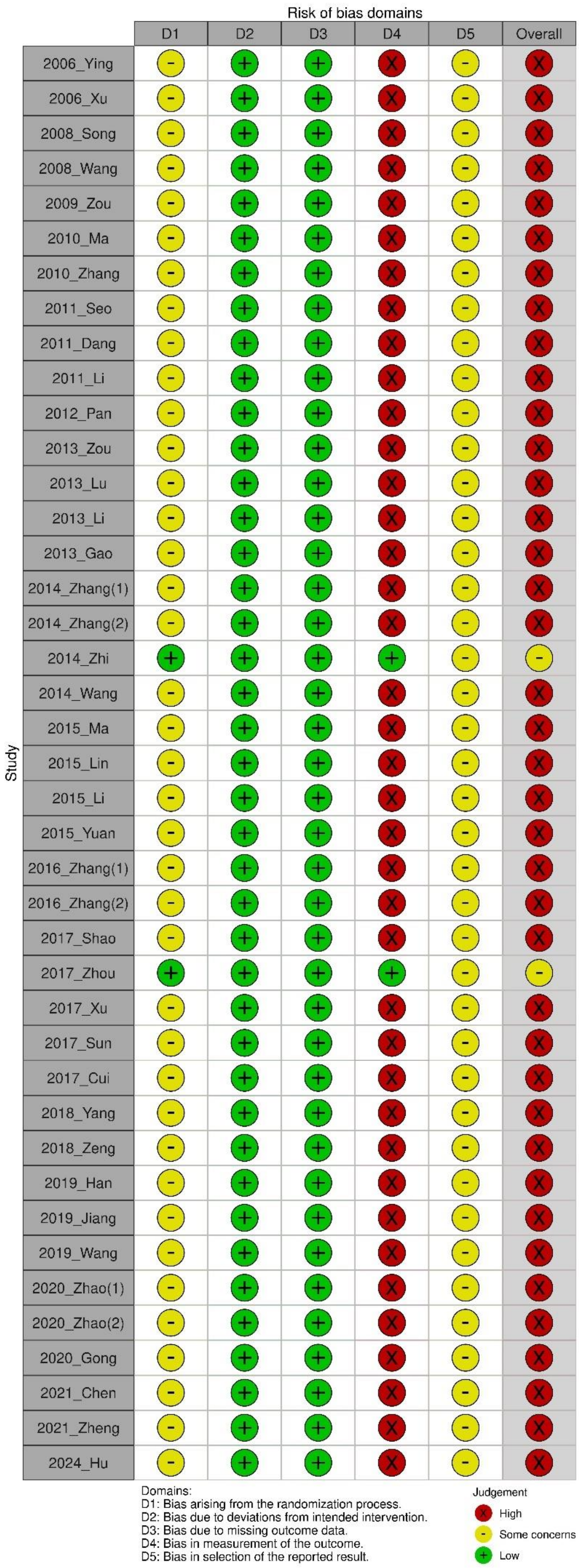

Supplementary Figure 2-1. Subgroup analysis

2.1.1 HAMD subgroup analysis

2.1.2 HAMD Outcome with VS without Criteria

2.1.3 HAMD Outcome with Criteria : Mild VS Moderate

2.1.4 TER subgroup analysis

2.1.5 TER Outcome with VS without Criteria

2.1.6 TER Outcome with Criteria : Mild VS Moderate

2.1.7 MBI subgroup analysis

2.1.8 SSS subgroup analysis

2.1.1

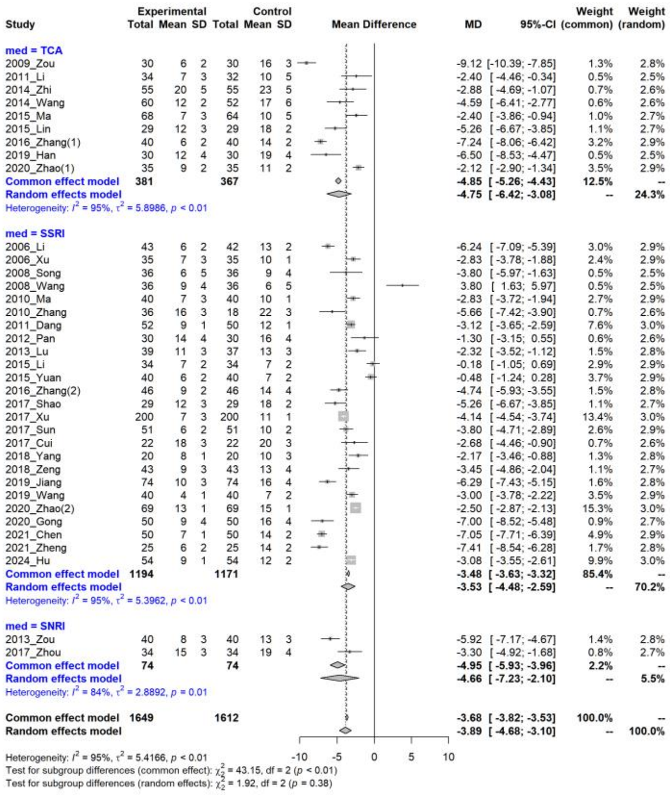
Study | Experimental Total Mean SD | Control Total Mean SD | Mean Difference | MD 95%-CI | Weight (common) | Weight (random)
med = TCA
2009_Zou 30 6 2 30 16 3 -9.12 [-10.39; -7.85] 1.3% 2.8%
2011_Li 34 7 3 32 10 5 -2.40 [-4.46; -0.34] 0.5% 2.5%
2014_Zhi 55 20 5 55 23 5 -2.88 [-4.69; -1.07] 0.7% 2.6%
2014_Wang 60 12 2 52 17 6 -4.59 [-6.41; -2.77] 0.6% 2.6%
2015_Ma 68 7 3 64 10 5 -2.40 [-3.86; -0.94] 1.0% 2.7%
2015_Lin 29 12 3 29 18 2 -5.26 [-6.67; -3.85] 1.1% 2.7%
2016_Zhang(1) 40 6 2 40 14 2 -7.24 [-8.06; -6.42] 3.2% 2.9%
2019_Han 30 12 4 30 19 4 -6.50 [-8.53; -4.47] 0.5% 2.5%
2020_Zhao(1) 35 9 2 35 11 2 -2.12 [-2.90; -1.34] 3.5% 2.9%
Common effect model 381 367 -4.85 [-5.26; -4.43] 12.5% --
Random effects model -4.75 [-6.42; -3.08] -- 24.3%
Heterogeneity: I^2 = 95%, τ^2 = 5.8986, p < 0.01
med = SSRI
2006_Li 43 6 2 42 13 2 -6.24 [-7.09; -5.39] 3.0% 2.9%
2006_Xu 35 7 3 35 10 1 -2.83 [-3.78; -1.88] 2.4% 2.9%
2008_Song 36 6 5 36 9 4 -3.80 [-5.97; -1.63] 0.5% 2.5%
2008_Wang 36 9 4 36 6 5 3.80 [1.63; 5.97] 0.5% 2.5%
2010_Ma 40 7 3 40 10 1 -2.83 [-3.72; -1.94] 2.7% 2.9%
2010_Zhang 36 16 3 18 22 3 -5.66 [-7.42; -3.90] 0.7% 2.6%
2011_Dang 52 9 1 50 12 1 -3.12 [-3.65; -2.59] 7.6% 3.0%
2012_Pan 30 14 4 30 16 4 -1.30 [-3.15; 0.55] 0.6% 2.6%
2013_Lu 39 11 3 37 13 3 -2.32 [-3.52; -1.12] 1.5% 2.8%
2015_Li 34 7 2 34 7 2 -0.18 [-1.05; 0.69] 2.9% 2.9%
2015_Yuan 40 6 2 40 7 2 -0.48 [-1.24; 0.28] 3.7% 2.9%
2016_Zhang(2) 46 9 2 46 14 4 -4.74 [-5.93; -3.55] 1.5% 2.8%
2017_Shao 29 12 3 29 18 2 -5.26 [-6.67; -3.85] 1.1% 2.7%
2017_Xu 200 7 3 200 11 1 -4.14 [-4.54; -3.74] 13.4% 3.0%
2017_Sun 51 6 2 51 10 2 -3.80 [-4.71; -2.89] 2.6% 2.9%
2017_Cui 22 18 3 22 20 3 -2.68 [-4.46; -0.90] 0.7% 2.6%
2018_Yang 20 8 1 20 10 3 -2.17 [-3.46; -0.88] 1.3% 2.8%
2018_Zeng 43 9 3 43 13 4 -3.45 [-4.86; -2.04] 1.1% 2.7%
2019_Jiang 74 10 3 74 16 4 -6.29 [-7.43; -5.15] 1.6% 2.8%
2019_Wang 40 4 1 40 7 2 -3.00 [-3.78; -2.22] 3.5% 2.9%
2020_Zhao(2) 69 13 1 69 15 1 -2.50 [-2.87; -2.13] 15.3% 3.0%
2020_Gong 50 9 4 50 16 4 -7.00 [-8.52; -5.48] 0.9% 2.7%
2021_Chen 50 7 1 50 14 2 -7.05 [-7.71; -6.39] 4.9% 2.9%
2021_Zheng 25 6 2 25 14 2 -7.41 [-8.54; -6.28] 1.7% 2.8%
2024_Hu 54 9 1 54 12 2 -3.08 [-3.55; -2.61] 9.9% 3.0%
Common effect model 1194 1171 -3.48 [-3.63; -3.32] 85.4% --
Random effects model -3.53 [-4.48; -2.59] -- 70.2%
Heterogeneity: I^2 = 95%, τ^2 = 5.3962, p < 0.01
med = SNRI
2013_Zou 40 8 3 40 13 3 -5.92 [-7.17; -4.67] 1.4% 2.8%
2017_Zhou 34 15 3 34 19 4 -3.30 [-4.92; -1.68] 0.8% 2.7%
Common effect model 74 74 -4.95 [-5.93; -3.96] 2.2% --
Random effects model -4.66 [-7.23; -2.10] -- 5.5%
Heterogeneity: I^2 = 84%, τ^2 = 2.8892, p = 0.01
Common effect model 1649 1612 -3.68 [-3.82; -3.53] 100.0% --
Random effects model -3.89 [-4.68; -3.10] -- 100.0%
-10 -5 0 5 10
Heterogeneity: I^2 = 95%, τ^2 = 5.4166, p < 0.01
Test for subgroup differences (common effect): χ^2_2 = 43.15, df = 2 (p < 0.01)
Test for subgroup differences (random effects): χ^2_2 = 1.92, df = 2 (p = 0.38)

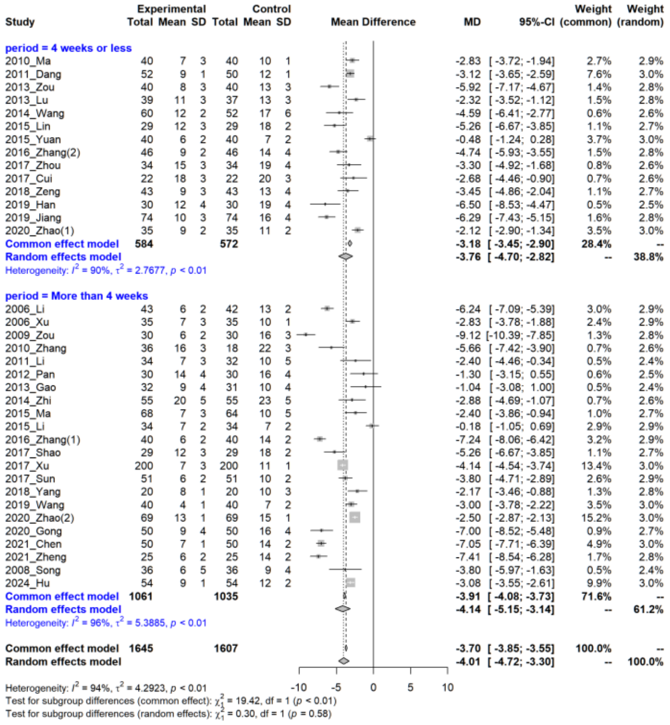
Study | Experimental Total Mean SD | Control Total Mean SD | Mean Difference | MD 95%-CI | Weight (common) | Weight (random)
period = 4 weeks or less
2010_Ma 40 7 3 40 10 1 -2.83 [-3.72; -1.94] 2.7% 2.9%
2011_Dang 52 9 1 50 12 1 -3.12 [-3.65; -2.59] 7.6% 3.0%
2013_Zou 40 8 3 40 13 3 -5.92 [-7.17; -4.67] 1.4% 2.8%
2013_Lu 39 11 3 37 13 3 -2.32 [-3.52; -1.12] 1.5% 2.8%
2014_Wang 60 12 2 52 17 6 -4.59 [-6.41; -2.77] 0.6% 2.6%
2015_Lin 29 12 3 29 18 2 -5.26 [-6.67; -3.85] 1.1% 2.7%
2015_Yuan 40 6 2 40 7 2 -0.48 [-1.24; 0.28] 3.7% 3.0%
2016_Zhang(2) 46 9 2 46 14 4 -4.74 [-5.93; -3.55] 1.5% 2.8%
2017_Zhou 34 15 3 34 19 4 -3.30 [-4.92; -1.68] 0.8% 2.6%
2017_Cui 22 18 3 22 20 3 -2.68 [-4.46; -0.90] 0.7% 2.6%
2018_Zeng 43 9 3 43 13 4 -3.45 [-4.86; -2.04] 1.1% 2.7%
2019_Han 30 12 4 30 19 4 -6.50 [-8.53; -4.47] 0.5% 2.5%
2019_Jiang 74 10 3 74 16 4 -6.29 [-7.43; -5.15] 1.6% 2.8%
2020_Zhao(1) 35 9 2 35 11 2 -2.12 [-2.90; -1.34] 3.5% 3.0%
Common effect model 584 572 -3.18 [-3.45; -2.90] 28.4% --
Random effects model -3.76 [-4.70; -2.82] -- 38.8%
Heterogeneity: I^2 = 90%, τ^2 = 2.7677, p < 0.01
period = More than 4 weeks
2006_Li 43 6 2 42 13 2 -6.24 [-7.09; -5.39] 3.0% 2.9%
2006_Xu 35 7 3 35 10 1 -2.83 [-3.78; -1.88] 2.4% 2.9%
2009_Zou 30 6 2 30 16 3 -9.12 [-10.39; -7.85] 1.3% 2.8%
2010_Zhang 36 16 3 18 22 3 -5.66 [-7.42; -3.90] 0.7% 2.6%
2011_Li 34 7 3 32 10 5 -2.40 [-4.46; -0.34] 0.5% 2.4%
2012_Pan 30 14 4 30 16 4 -1.30 [-3.15; 0.55] 0.6% 2.5%
2013_Gao 32 9 4 31 10 4 -1.04 [-3.08; 1.00] 0.5% 2.4%
2014_Zhi 55 20 5 55 23 5 -2.88 [-4.69; -1.07] 0.7% 2.6%
2015_Ma 68 7 3 64 10 5 -2.40 [-3.86; -0.94] 1.0% 2.7%
2015_Li 34 7 2 34 7 2 -0.18 [-1.05; 0.69] 2.9% 2.9%
2016_Zhang(1) 40 6 2 40 14 2 -7.24 [-8.06; -6.42] 3.2% 2.9%
2017_Shao 29 12 3 29 18 2 -5.26 [-6.67; -3.85] 1.1% 2.7%
2017_Xu 200 7 3 200 11 1 -4.14 [-4.54; -3.74] 13.4% 3.0%
2017_Sun 51 6 2 51 10 2 -3.80 [-4.71; -2.89] 2.6% 2.9%
2018_Yang 20 8 1 20 10 3 -2.17 [-3.46; -0.88] 1.3% 2.8%
2019_Wang 40 4 1 40 7 2 -3.00 [-3.78; -2.22] 3.5% 3.0%
2020_Zhao(2) 69 13 1 69 15 1 -2.50 [-2.87; -2.13] 15.2% 3.0%
2020_Gong 50 9 4 50 16 4 -7.00 [-8.52; -5.48] 0.9% 2.7%
2021_Chen 50 7 1 50 14 2 -7.05 [-7.71; -6.39] 4.9% 3.0%
2021_Zheng 25 6 2 25 14 2 -7.41 [-8.54; -6.28] 1.7% 2.8%
2008_Song 36 6 5 36 9 4 -3.80 [-5.97; -1.63] 0.5% 2.4%
2024_Hu 54 9 1 54 12 2 -3.08 [-3.55; -2.61] 9.9% 3.0%
Common effect model 1061 1035 -3.91 [-4.08; -3.73] 71.6% --
Random effects model -4.14 [-5.15; -3.14] -- 61.2%
Heterogeneity: I^2 = 96%, τ^2 = 5.3885, p < 0.01
Common effect model 1645 1607 -3.70 [-3.85; -3.55] 100.0% --
Random effects model -4.01 [-4.72; -3.30] -- 100.0%
-10 -5 0 5 10
Heterogeneity: I^2 = 94%, τ^2 = 4.2923, p < 0.01
Test for subgroup differences (common effect): χ^2_1 = 19.42, df = 1 (p < 0.01)
Test for subgroup differences (random effects): χ^2_1 = 0.30, df = 1 (p = 0.58)

Study	Experimental Total	Experimental Mean	Experimental SD	Control Total	Control Mean	Control SD	MD	95%-CI	Weight (common)	Weight (random)
**team = None**										
2006_Li	43	6	2	42	13	2	-6.24	[-7.09; -5.39]	3.0%	2.9%
2008_Song	36	6	5	36	9	4	-3.80	[-5.97; -1.63]	0.5%	2.4%
2009_Zou	30	6	2	30	16	3	-9.12	[-10.39; -7.85]	1.3%	2.8%
2010_Zhang	36	16	3	18	22	3	-5.66	[-7.42; -3.90]	0.7%	2.6%
2011_Dang	52	9	1	50	12	1	-3.12	[-3.65; -2.59]	7.6%	3.0%
2011_Li	34	7	3	32	10	5	-2.40	[-4.46; -0.34]	0.5%	2.4%
2012_Pan	30	14	4	30	16	4	-1.30	[-3.15; 0.55]	0.6%	2.5%
2013_Zou	40	8	3	40	13	3	-5.92	[-7.17; -4.67]	1.4%	2.8%
2014_Zhi	55	20	5	55	23	5	-2.88	[-4.69; -1.07]	0.7%	2.6%
2014_Wang	60	12	2	52	17	6	-4.59	[-6.41; -2.77]	0.6%	2.6%
2015_Ma	68	7	3	64	10	5	-2.40	[-3.86; -0.94]	1.0%	2.7%
2015_Lin	29	12	3	29	18	2	-5.26	[-6.67; -3.85]	1.1%	2.7%
2015_Li	34	7	2	34	7	2	-0.18	[-1.05; 0.69]	2.9%	2.9%
2015_Yuan	40	6	2	40	7	2	-0.48	[-1.24; 0.28]	3.7%	3.0%
2016_Zhang(1)	40	6	2	40	14	2	-7.24	[-8.06; -6.42]	3.2%	2.9%
2016_Zhang(2)	46	9	2	46	14	4	-4.74	[-5.93; -3.55]	1.5%	2.8%
2017_Zhou	34	15	3	34	19	4	-3.30	[-4.92; -1.68]	0.8%	2.6%
2017_Xu	200	7	3	200	11	1	-4.14	[-4.54; -3.74]	13.4%	3.0%
2017_Sun	51	6	2	51	10	2	-3.80	[-4.71; -2.89]	2.6%	2.9%
2017_Cui	22	18	3	22	20	3	-2.68	[-4.46; -0.90]	0.7%	2.6%
2018_Yang	20	8	1	20	10	3	-2.17	[-3.46; -0.88]	1.3%	2.8%
2018_Zeng	43	9	3	43	13	4	-3.45	[-4.86; -2.04]	1.1%	2.7%
2019_Wang	40	4	1	40	7	2	-3.00	[-3.78; -2.22]	3.5%	3.0%
2020_Gong	50	9	4	50	16	4	-7.00	[-8.52; -5.48]	0.9%	2.7%
2021_Zheng	25	6	2	25	14	2	-7.41	[-8.54; -6.28]	1.7%	2.8%
**Common effect model**	**1158**			**1123**			**-3.95**	**[-4.14; -3.75]**	**56.1%**	**--**
**Random effects model**							**-4.10**	**[-5.00; -3.21]**	**--**	**68.9%**
Heterogeneity: $I^2 = 94\%$, $\tau^2 = 4.6839$, $p < 0.01$										
**team = TEAM**										
2006_Xu	35	7	3	35	10	1	-2.83	[-3.78; -1.88]	2.4%	2.9%
2010_Ma	40	7	3	40	10	1	-2.83	[-3.72; -1.94]	2.7%	2.9%
2013_Lu	39	11	3	37	13	3	-2.32	[-3.52; -1.12]	1.5%	2.8%
2013_Gao	32	9	4	31	10	4	-1.04	[-3.08; 1.00]	0.5%	2.4%
2017_Shao	29	12	3	29	18	2	-5.26	[-6.67; -3.85]	1.1%	2.7%
2019_Han	30	12	4	30	19	4	-6.50	[-8.53; -4.47]	0.5%	2.5%
2019_Jiang	74	10	3	74	16	4	-6.29	[-7.43; -5.15]	1.6%	2.8%
2020_Zhao(1)	35	9	2	35	11	2	-2.12	[-2.90; -1.34]	3.5%	3.0%
2020_Zhao(2)	69	13	1	69	15	1	-2.50	[-2.87; -2.13]	15.2%	3.0%
2021_Chen	50	7	1	50	14	2	-7.05	[-7.71; -6.39]	4.9%	3.0%
2024_Hu	54	9	1	54	12	2	-3.08	[-3.55; -2.61]	9.9%	3.0%
**Common effect model**	**487**			**484**			**-3.38**	**[-3.60; -3.16]**	**43.9%**	**--**
**Random effects model**							**-3.79**	**[-4.98; -2.60]**	**--**	**31.1%**
Heterogeneity: $I^2 = 95\%$, $\tau^2 = 3.7246$, $p < 0.01$										
**Common effect model**	**1645**			**1607**			**-3.70**	**[-3.85; -3.55]**	**100.0%**	**--**
**Random effects model**							**-4.01**	**[-4.72; -3.30]**	**--**	**100.0%**

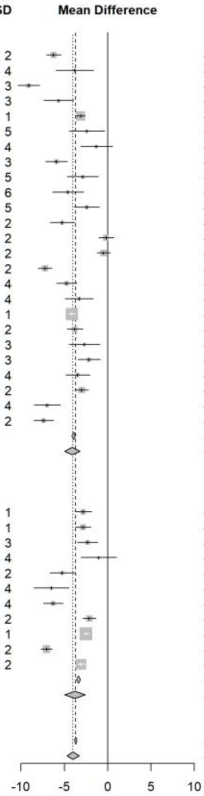

Heterogeneity: $I^2 = 94\%$, $\tau^2 = 4.2923$, $p < 0.01$
Test for subgroup differences (common effect): $\chi^2_1 = 14.00$, df = 1 ($p < 0.01$)
Test for subgroup differences (random effects): $\chi^2_1 = 0.17$, df = 1 ($p = 0.68$)

Study	Experimental Total	Experimental Mean	Experimental SD	Control Total	Control Mean	Control SD	MD	95%-CI	Weight (common)	Weight (random)
**substance = decoction**										
2006_Li	43	6	2	42	13	2	-6.24	[-7.09; -5.39]	3.0%	2.9%
2008_Song	36	6	5	36	9	4	-3.80	[-5.97; -1.63]	0.5%	2.4%
2010_Ma	40	7	3	40	10	1	-2.83	[-3.72; -1.94]	2.7%	2.9%
2010_Zhang	36	16	3	18	22	3	-5.66	[-7.42; -3.90]	0.7%	2.6%
2011_Dang	52	9	1	50	12	1	-3.12	[-3.65; -2.59]	7.6%	3.0%
2011_Li	34	7	3	32	10	5	-2.40	[-4.46; -0.34]	0.5%	2.4%
2012_Pan	30	14	4	30	16	4	-1.30	[-3.15; 0.55]	0.6%	2.5%
2013_Lu	39	11	3	37	13	3	-2.32	[-3.52; -1.12]	1.5%	2.8%
2015_Ma	68	7	3	64	10	5	-2.40	[-3.86; -0.94]	1.0%	2.7%
2015_Lin	29	12	3	29	18	2	-5.26	[-6.67; -3.85]	1.1%	2.7%
2015_Li	34	7	2	34	7	2	-0.18	[-1.05; 0.69]	2.9%	2.9%
2015_Yuan	40	6	2	40	7	2	-0.48	[-1.24; 0.28]	3.7%	3.0%
2016_Zhang(2)	46	9	2	46	14	4	-4.74	[-5.93; -3.55]	1.5%	2.8%
2017_Shao	29	12	3	29	18	2	-5.26	[-6.67; -3.85]	1.1%	2.7%
2017_Sun	51	6	2	51	10	2	-3.80	[-4.71; -2.89]	2.6%	2.9%
2017_Cui	22	18	3	22	20	3	-2.68	[-4.46; -0.90]	0.7%	2.6%
2018_Yang	20	8	1	20	10	3	-2.17	[-3.46; -0.88]	1.3%	2.8%
2019_Han	30	12	4	30	19	4	-6.50	[-8.53; -4.47]	0.5%	2.5%
2019_Wang	40	4	1	40	7	2	-3.00	[-3.78; -2.22]	3.5%	3.0%
2020_Zhao(1)	35	9	2	35	11	2	-2.12	[-2.90; -1.34]	3.5%	3.0%
2020_Zhao(2)	69	13	1	69	15	1	-2.50	[-2.87; -2.13]	15.2%	3.0%
2020_Gong	50	9	4	50	16	4	-7.00	[-8.52; -5.48]	0.9%	2.7%
2021_Chen	50	7	1	50	14	2	-7.05	[-7.71; -6.39]	4.9%	3.0%
2021_Zheng	25	6	2	25	14	2	-7.41	[-8.54; -6.28]	1.7%	2.8%
**Common effect model**	**948**			**919**			**-3.35**	**[-3.54; -3.17]**	**63.1%**	**--**
**Random effects model**							**-3.74**	**[-4.59; -2.89]**	**--**	**66.7%**
Heterogeneity: $I^2 = 95\%$, $\tau^2 = 4.1204$, $p < 0.01$										
**substance = granule**										
2006_Xu	35	7	3	35	10	1	-2.83	[-3.78; -1.88]	2.4%	2.9%
2013_Zou	40	8	3	40	13	3	-5.92	[-7.17; -4.67]	1.4%	2.8%
2013_Gao	32	9	4	31	10	4	-1.04	[-3.08; 1.00]	0.5%	2.4%
2014_Wang	60	12	2	52	17	6	-4.59	[-6.41; -2.77]	0.6%	2.6%
2017_Zhou	34	15	3	34	19	4	-3.30	[-4.92; -1.68]	0.8%	2.6%
2017_Xu	200	7	3	200	11	1	-4.14	[-4.54; -3.74]	13.4%	3.0%
2019_Jiang	74	10	3	74	16	4	-6.29	[-7.43; -5.15]	1.6%	2.8%
**Common effect model**	**475**			**466**			**-4.18**	**[-4.50; -3.86]**	**20.7%**	**--**
**Random effects model**							**-4.10**	**[-5.38; -2.81]**	**--**	**19.2%**
Heterogeneity: $I^2 = 85\%$, $\tau^2 = 2.5161$, $p < 0.01$										
**substance = pill**										
2009_Zou	30	6	2	30	16	3	-9.12	[-10.39; -7.85]	1.3%	2.8%
2014_Zhi	55	20	5	55	23	5	-2.88	[-4.69; -1.07]	0.7%	2.6%
2016_Zhang(1)	40	6	2	40	14	2	-7.24	[-8.06; -6.42]	3.2%	2.9%
2018_Zeng	43	9	3	43	13	4	-3.45	[-4.86; -2.04]	1.1%	2.7%
2024_Hu	54	9	1	54	12	2	-3.08	[-3.55; -2.61]	9.9%	3.0%
**Common effect model**	**222**			**222**			**-4.42**	**[-4.79; -4.06]**	**16.1%**	**--**
**Random effects model**							**-5.17**	**[-7.67; -2.68]**	**--**	**14.1%**
Heterogeneity: $I^2 = 97\%$, $\tau^2 = 7.6999$, $p < 0.01$										
**Common effect model**	**1645**			**1607**			**-3.70**	**[-3.85; -3.55]**	**100.0%**	**--**
**Random effects model**							**-4.01**	**[-4.72; -3.30]**	**--**	**100.0%**

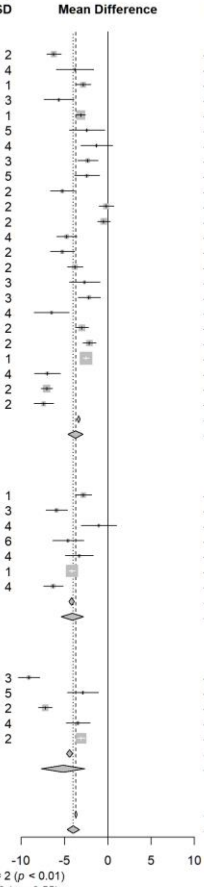

Heterogeneity: $I^2 = 94\%$, $\tau^2 = 4.2923$, $p < 0.01$
Test for subgroup differences (common effect): $\chi^2_2 = 37.25$, df = 2 ($p < 0.01$)
Test for subgroup differences (random effects): $\chi^2_2 = 1.21$, df = 2 ($p = 0.55$)

2.1.2

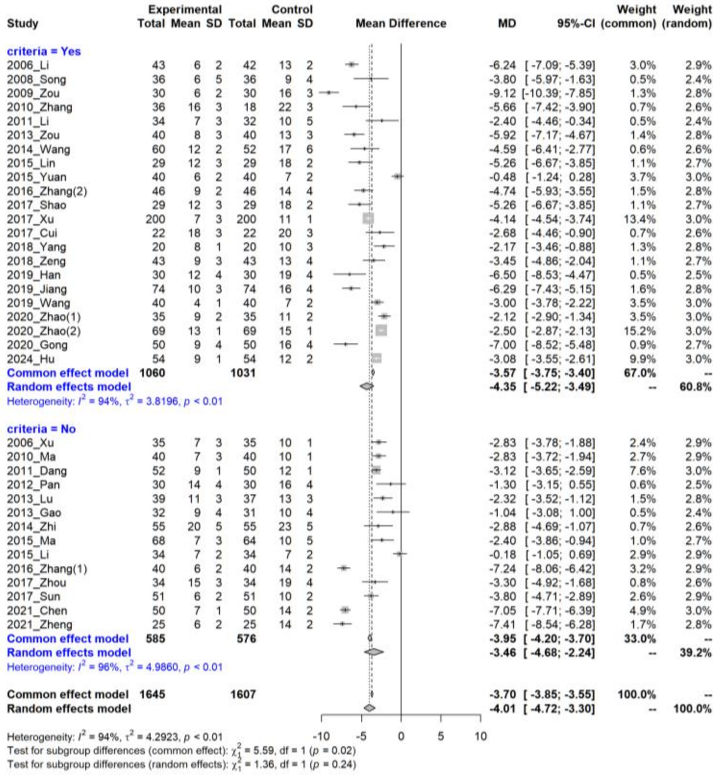
Study Experimental Total Mean SD Control Total Mean SD Mean Difference MD 95%-CI Weight (common) Weight (random)
criteria = Yes
2006_Li 43 6 2 42 13 2 -6.24 [-7.09; -5.39] 3.0% 2.9%
2008_Song 36 6 5 36 9 4 -3.80 [-5.97; -1.63] 0.5% 2.4%
2009_Zou 30 6 2 30 16 3 -9.12 [-10.39; -7.85] 1.3% 2.8%
2010_Zhang 36 16 3 18 22 3 -5.66 [-7.42; -3.90] 0.7% 2.6%
2011_Li 34 7 3 32 10 5 -2.40 [-4.46; -0.34] 0.5% 2.4%
2013_Zou 40 8 3 40 13 3 -5.92 [-7.17; -4.67] 1.4% 2.8%
2014_Wang 60 12 2 52 17 6 -4.59 [-6.41; -2.77] 0.6% 2.6%
2015_Lin 29 12 3 29 18 2 -5.26 [-6.67; -3.85] 1.1% 2.7%
2015_Yuan 40 6 2 40 7 2 -0.48 [-1.24; 0.28] 3.7% 3.0%
2016_Zhang(2) 46 9 2 46 14 4 -4.74 [-5.93; -3.55] 1.5% 2.8%
2017_Shao 29 12 3 29 18 2 -5.26 [-6.67; -3.85] 1.1% 2.7%
2017_Xu 200 7 3 200 11 1 -4.14 [-4.54; -3.74] 13.4% 3.0%
2017_Cui 22 18 3 22 20 3 -2.68 [-4.46; -0.90] 0.7% 2.6%
2018_Yang 20 8 1 20 10 3 -2.17 [-3.46; -0.88] 1.3% 2.8%
2018_Zeng 43 9 3 43 13 4 -3.45 [-4.86; -2.04] 1.1% 2.7%
2019_Han 30 12 4 30 19 4 -6.50 [-8.53; -4.47] 0.5% 2.5%
2019_Jiang 74 10 3 74 16 4 -6.29 [-7.43; -5.15] 1.6% 2.8%
2019_Wang 40 4 1 40 7 2 -3.00 [-3.78; -2.22] 3.5% 3.0%
2020_Zhao(1) 35 9 2 35 11 2 -2.12 [-2.90; -1.34] 3.5% 3.0%
2020_Zhao(2) 69 13 1 69 15 1 -2.50 [-2.87; -2.13] 15.2% 3.0%
2020_Gong 50 9 4 50 16 4 -7.00 [-8.52; -5.48] 0.9% 2.7%
2024_Hu 54 9 1 54 12 2 -3.08 [-3.55; -2.61] 9.9% 3.0%
Common effect model 1060 1031 -3.57 [-3.75; -3.40] 67.0% --
Random effects model -4.35 [-5.22; -3.49] -- 60.8%
Heterogeneity: I² = 94%, τ² = 3.8196, p < 0.01
criteria = No
2006_Xu 35 7 3 35 10 1 -2.83 [-3.78; -1.88] 2.4% 2.9%
2010_Ma 40 7 3 40 10 1 -2.83 [-3.72; -1.94] 2.7% 2.9%
2011_Dang 52 9 1 50 12 1 -3.12 [-3.65; -2.59] 7.6% 3.0%
2012_Pan 30 14 4 30 16 4 -1.30 [-3.15; 0.55] 0.6% 2.5%
2013_Lu 39 11 3 37 13 3 -2.32 [-3.52; -1.12] 1.5% 2.8%
2013_Gao 32 9 4 31 10 4 -1.04 [-3.08; 1.00] 0.5% 2.4%
2014_Zhi 55 20 5 55 23 5 -2.88 [-4.69; -1.07] 0.7% 2.6%
2015_Ma 68 7 3 64 10 5 -2.40 [-3.86; -0.94] 1.0% 2.7%
2015_Li 34 7 2 34 7 2 -0.18 [-1.05; 0.69] 2.9% 2.9%
2016_Zhang(1) 40 6 2 40 14 2 -7.24 [-8.06; -6.42] 3.2% 2.9%
2017_Zhou 34 15 3 34 19 4 -3.30 [-4.92; -1.68] 0.8% 2.6%
2017_Sun 51 6 2 51 10 2 -3.80 [-4.71; -2.89] 2.6% 2.9%
2021_Chen 50 7 1 50 14 2 -7.05 [-7.71; -6.39] 4.9% 3.0%
2021_Zheng 25 6 2 25 14 2 -7.41 [-8.54; -6.28] 1.7% 2.8%
Common effect model 585 576 -3.95 [-4.20; -3.70] 33.0% --
Random effects model -3.46 [-4.68; -2.24] -- 39.2%
Heterogeneity: I² = 96%, τ² = 4.9860, p < 0.01
Common effect model 1645 1607 -3.70 [-3.85; -3.55] 100.0% --
Random effects model -4.01 [-4.72; -3.30] -- 100.0%
Heterogeneity: I² = 94%, τ² = 4.2923, p < 0.01
-10 -5 0 5 10
Test for subgroup differences (common effect): χ²₁ = 5.59, df = 1 (p = 0.02)
Test for subgroup differences (random effects): χ²₁ = 1.36, df = 1 (p = 0.24)

2.1.3

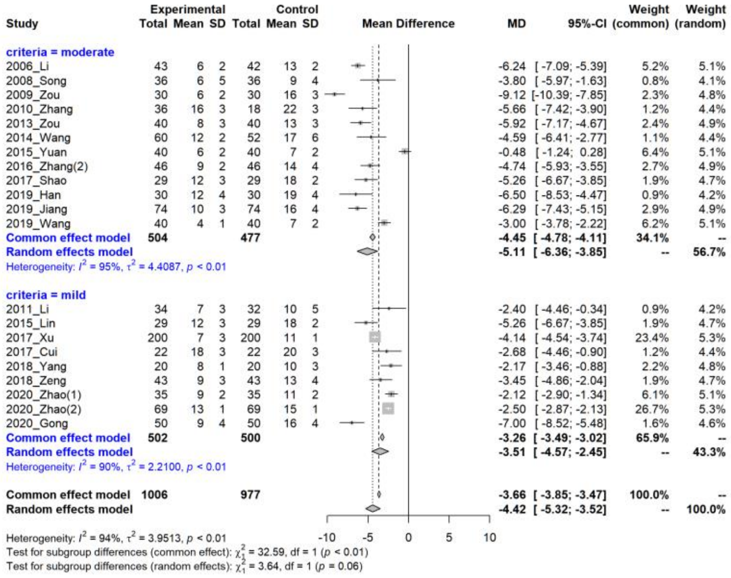
Study Experimental Total Mean SD Control Total Mean SD Mean Difference MD 95%-CI Weight (common) Weight (random)
criteria = moderate
2006_Li 43 6 2 42 13 2 -6.24 [-7.09; -5.39] 5.2% 5.1%
2008_Song 36 6 5 36 9 4 -3.80 [-5.97; -1.63] 0.8% 4.1%
2009_Zou 30 6 2 30 16 3 -9.12 [-10.39; -7.85] 2.3% 4.8%
2010_Zhang 36 16 3 18 22 3 -5.66 [-7.42; -3.90] 1.2% 4.4%
2013_Zou 40 8 3 40 13 3 -5.92 [-7.17; -4.67] 2.4% 4.9%
2014_Wang 60 12 2 52 17 6 -4.59 [-6.41; -2.77] 1.1% 4.4%
2015_Yuan 40 6 2 40 7 2 -0.48 [-1.24; 0.28] 6.4% 5.1%
2016_Zhang(2) 46 9 2 46 14 4 -4.74 [-5.93; -3.55] 2.7% 4.9%
2017_Shao 29 12 3 29 18 2 -5.26 [-6.67; -3.85] 1.9% 4.7%
2019_Han 30 12 4 30 19 4 -6.50 [-8.53; -4.47] 0.9% 4.2%
2019_Jiang 74 10 3 74 16 4 -6.29 [-7.43; -5.15] 2.9% 4.9%
2019_Wang 40 4 1 40 7 2 -3.00 [-3.78; -2.22] 6.2% 5.1%
Common effect model 504 477 -4.45 [-4.78; -4.11] 34.1% --
Random effects model -5.11 [-6.36; -3.85] -- 56.7%
Heterogeneity: I² = 95%, τ² = 4.4087, p < 0.01
criteria = mild
2011_Li 34 7 3 32 10 5 -2.40 [-4.46; -0.34] 0.9% 4.2%
2015_Lin 29 12 3 29 18 2 -5.26 [-6.67; -3.85] 1.9% 4.7%
2017_Xu 200 7 3 200 11 1 -4.14 [-4.54; -3.74] 23.4% 5.3%
2017_Cui 22 18 3 22 20 3 -2.68 [-4.46; -0.90] 1.2% 4.4%
2018_Yang 20 8 1 20 10 3 -2.17 [-3.46; -0.88] 2.2% 4.8%
2018_Zeng 43 9 3 43 13 4 -3.45 [-4.86; -2.04] 1.9% 4.7%
2020_Zhao(1) 35 9 2 35 11 2 -2.12 [-2.90; -1.34] 6.1% 5.1%
2020_Zhao(2) 69 13 1 69 15 1 -2.50 [-2.87; -2.13] 26.7% 5.3%
2020_Gong 50 9 4 50 16 4 -7.00 [-8.52; -5.48] 1.6% 4.6%
Common effect model 502 500 -3.26 [-3.49; -3.02] 65.9% --
Random effects model -3.51 [-4.57; -2.45] -- 43.3%
Heterogeneity: I² = 90%, τ² = 2.2100, p < 0.01
Common effect model 1006 977 -3.66 [-3.85; -3.47] 100.0% --
Random effects model -4.42 [-5.32; -3.52] -- 100.0%
Heterogeneity: I² = 94%, τ² = 3.9513, p < 0.01
-10 -5 0 5 10
Test for subgroup differences (common effect): χ²₁ = 32.59, df = 1 (p < 0.01)
Test for subgroup differences (random effects): χ²₁ = 3.64, df = 1 (p = 0.06)

2.1.4

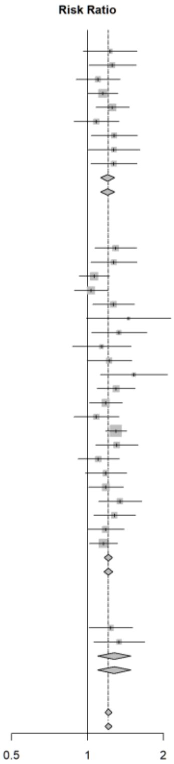

Study	Experimental Events	Experimental Total	Control Events	Control Total	RR	95%-CI	Weight (common)	Weight (random)
**med = TCA**								
2009_Zou	27	30	22	30	1.23	[0.96; 1.57]	1.6%	1.6%
2011_Li	32	34	24	32	1.25	[1.01; 1.56]	2.1%	2.1%
2014_Zhi	44	54	40	54	1.10	[0.90; 1.35]	2.4%	2.4%
2015_Wang	57	60	43	52	1.15	[1.00; 1.32]	5.3%	5.3%
2015_Ma	64	68	48	64	1.25	[1.08; 1.46]	4.2%	4.2%
2015_Lin	26	29	24	29	1.08	[0.88; 1.33]	2.3%	2.3%
2019_Zhang(1)	37	40	29	40	1.28	[1.03; 1.57]	2.3%	2.3%
2019_Han	28	30	22	30	1.27	[1.01; 1.61]	1.8%	1.8%
2020_Zhao(1)	33	35	26	35	1.27	[1.03; 1.57]	2.2%	2.2%
**Common effect model**		**380**		**366**	**1.20**	**[1.13; 1.28]**	**24.3%**	--
**Random effects model**					**1.20**	**[1.13; 1.28]**	--	**24.3%**
Heterogeneity: $I^2 = 0\%$, $\tau^2 = 0$, $p = 0.91$								
**med = SSRI**								
2006_Li	41	43	31	42	1.29	[1.07; 1.57]	2.7%	2.7%
2006_Xu	33	35	26	35	1.27	[1.03; 1.57]	2.2%	2.2%
2008_Song	34	36	32	36	1.06	[0.92; 1.22]	5.1%	5.1%
2008_Wang	33	36	32	36	1.03	[0.89; 1.20]	4.3%	4.3%
2010_Ma	38	40	30	40	1.27	[1.04; 1.54]	2.7%	2.7%
2010_Zhang	32	36	11	18	1.45	[0.99; 2.14]	0.7%	0.7%
2011_Xu	28	30	21	30	1.33	[1.04; 1.72]	1.6%	1.6%
2012_Pan	25	30	22	30	1.14	[0.87; 1.49]	1.4%	1.4%
2013_Lu	36	39	28	37	1.22	[0.99; 1.50]	2.4%	2.4%
2013_Li	29	32	19	32	1.53	[1.12; 2.08]	1.1%	1.1%
2014_Zhang(1)	48	50	37	50	1.30	[1.09; 1.54]	3.3%	3.3%
2014_Zhang(2)	39	40	33	40	1.18	[1.02; 1.37]	4.4%	4.4%
2017_Shao	26	29	24	29	1.08	[0.88; 1.33]	2.3%	2.3%
2017_Xu	184	200	142	200	1.30	[1.18; 1.43]	10.5%	10.5%
2017_Sun	47	51	36	51	1.31	[1.07; 1.59]	2.6%	2.6%
2017_Cui	21	22	19	22	1.11	[0.91; 1.34]	2.8%	2.8%
2018_Zeng	39	43	33	43	1.18	[0.98; 1.43]	2.7%	2.7%
2019_Jiang	65	74	55	74	1.18	[1.01; 1.38]	4.0%	4.0%
2019_Wang	39	40	29	40	1.34	[1.10; 1.64]	2.6%	2.6%
2020_Gong	46	50	36	50	1.28	[1.06; 1.55]	2.7%	2.7%
2021_Chen	46	50	39	50	1.18	[1.00; 1.40]	3.5%	3.5%
2024_Hu	52	54	45	54	1.16	[1.01; 1.32]	5.9%	5.9%
**Common effect model**		**1060**		**1039**	**1.21**	**[1.16; 1.26]**	**71.4%**	--
**Random effects model**					**1.21**	**[1.16; 1.26]**	--	**71.4%**
Heterogeneity: $I^2 = 0\%$, $\tau^2 = 0.0006$, $p = 0.55$								
**med = SNRI**								
2013_Zou	37	40	30	40	1.23	[1.01; 1.51]	2.5%	2.5%
2017_Zhou	32	34	24	34	1.33	[1.06; 1.68]	1.8%	1.8%
**Common effect model**		**74**		**74**	**1.27**	**[1.10; 1.48]**	**4.3%**	--
**Random effects model**					**1.27**	**[1.10; 1.48]**	--	**4.3%**
Heterogeneity: $I^2 = 0\%$, $\tau^2 = 0$, $p = 0.62$								
**Common effect model**		**1514**		**1479**	**1.21**	**[1.17; 1.25]**	**100.0%**	--
**Random effects model**					**1.21**	**[1.17; 1.25]**	--	**100.0%**

Heterogeneity: $I^2 = 0\%$, $\tau^2 = 0$, $p = 0.85$
Test for subgroup differences (common effect): $\chi^2_2 = 0.51$, df = 2 ($p = 0.77$)
Test for subgroup differences (random effects): $\chi^2_2 = 0.52$, df = 2 ($p = 0.77$)

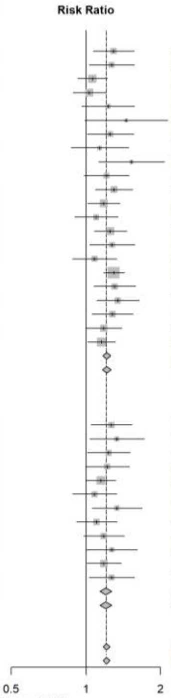

Study	Experimental Events	Experimental Total	Control Events	Control Total	RR	95%-CI	Weight (common)	Weight (random)
**period = More than 4 weeks**								
2006_Li	41	43	31	42	1.29	[1.07; 1.57]	2.6%	2.6%
2006_Xu	33	35	26	35	1.27	[1.03; 1.57]	2.2%	2.2%
2008_Song	34	36	32	36	1.06	[0.92; 1.22]	5.0%	5.0%
2008_Wang	33	36	32	36	1.03	[0.89; 1.20]	4.2%	4.2%
2009_Zou	27	30	22	30	1.23	[0.96; 1.57]	1.6%	1.6%
2010_Zhang	32	36	11	18	1.45	[0.99; 2.14]	0.7%	0.7%
2011_Li	32	34	24	32	1.25	[1.01; 1.56]	2.1%	2.1%
2012_Pan	25	30	22	30	1.14	[0.87; 1.49]	1.4%	1.4%
2013_Li	29	32	19	32	1.53	[1.12; 2.08]	1.0%	1.0%
2013_Gao	30	32	24	31	1.21	[0.98; 1.49]	2.2%	2.2%
2014_Zhang(1)	48	50	37	50	1.30	[1.09; 1.54]	3.2%	3.2%
2014_Zhang(2)	39	40	33	40	1.18	[1.02; 1.37]	4.3%	4.3%
2014_Zhi	44	54	40	54	1.10	[0.90; 1.35]	2.4%	2.4%
2015_Ma	64	68	48	64	1.25	[1.08; 1.46]	4.1%	4.1%
2016_Zhang(1)	37	40	29	40	1.28	[1.03; 1.57]	2.2%	2.2%
2017_Shao	26	29	24	29	1.08	[0.88; 1.33]	2.3%	2.3%
2017_Xu	184	200	142	200	1.30	[1.18; 1.43]	10.2%	10.2%
2017_Sun	47	51	36	51	1.31	[1.07; 1.59]	2.6%	2.6%
2019_Wang	39	40	29	40	1.34	[1.10; 1.64]	2.5%	2.5%
2020_Gong	46	50	36	50	1.28	[1.06; 1.55]	2.7%	2.7%
2021_Chen	46	50	39	50	1.18	[1.00; 1.40]	3.4%	3.4%
2024_Hu	52	54	45	54	1.16	[1.01; 1.32]	5.7%	5.7%
**Common effect model**		**1070**		**1044**	**1.21**	**[1.17; 1.26]**	**68.6%**	--
**Random effects model**					**1.21**	**[1.17; 1.26]**	--	**68.6%**
Heterogeneity: $I^2 = 0\%$, $\tau^2 = 0.0007$, $p = 0.57$								
**period = 4 weeks or less**								
2010_Ma	38	40	30	40	1.27	[1.04; 1.54]	2.6%	2.6%
2011_Xu	28	30	21	30	1.33	[1.04; 1.72]	1.5%	1.5%
2013_Zou	37	40	30	40	1.23	[1.01; 1.51]	2.4%	2.4%
2013_Lu	36	39	28	37	1.22	[0.99; 1.50]	2.3%	2.3%
2014_Wang	57	60	43	52	1.15	[1.00; 1.32]	5.2%	5.2%
2015_Lin	26	29	24	29	1.08	[0.88; 1.33]	2.3%	2.3%
2017_Zhou	32	34	24	34	1.33	[1.06; 1.68]	1.8%	1.8%
2017_Cui	21	22	19	22	1.11	[0.91; 1.34]	2.7%	2.7%
2018_Zeng	39	43	33	43	1.18	[0.98; 1.43]	2.7%	2.7%
2019_Han	28	30	22	30	1.27	[1.01; 1.61]	1.7%	1.7%
2019_Jiang	65	74	55	74	1.18	[1.01; 1.38]	3.9%	3.9%
2020_Zhao(1)	33	35	26	35	1.27	[1.03; 1.57]	2.2%	2.2%
**Common effect model**		**476**		**466**	**1.20**	**[1.14; 1.27]**	**31.4%**	--
**Random effects model**					**1.20**	**[1.14; 1.27]**	--	**31.4%**
Heterogeneity: $I^2 = 0\%$, $\tau^2 = 0$, $p = 0.95$								
**Common effect model**		**1546**		**1510**	**1.21**	**[1.17; 1.25]**	**100.0%**	--
**Random effects model**					**1.21**	**[1.17; 1.25]**	--	**100.0%**

Heterogeneity: $I^2 = 0\%$, $\tau^2 = 0$, $p = 0.88$
Test for subgroup differences (common effect): $\chi^2_1 = 0.08$, df = 1 ($p = 0.77$)
Test for subgroup differences (random effects): $\chi^2_1 = 0.08$, df = 1 ($p = 0.78$)

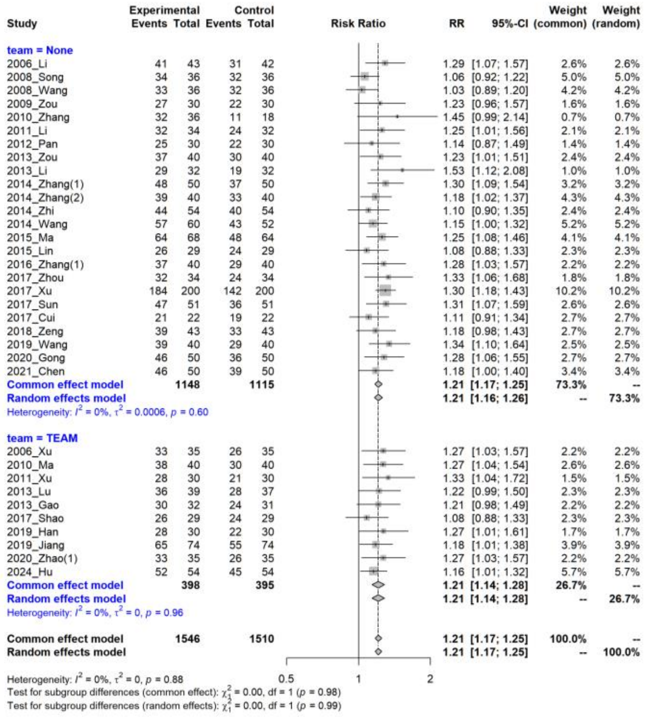
Experimental Control
Study Events Total Events Total Risk Ratio RR 95%-CI Weight (common) Weight (random)
team = None
2006_Li 41 43 31 42 1.29 [1.07; 1.57] 2.6% 2.6%
2008_Song 34 36 32 36 1.06 [0.92; 1.22] 5.0% 5.0%
2008_Wang 33 36 32 36 1.03 [0.89; 1.20] 4.2% 4.2%
2009_Zou 27 30 22 30 1.23 [0.96; 1.57] 1.6% 1.6%
2010_Zhang 32 36 11 18 1.45 [0.99; 2.14] 0.7% 0.7%
2011_Li 32 34 24 32 1.25 [1.01; 1.56] 2.1% 2.1%
2012_Pan 25 30 22 30 1.14 [0.87; 1.49] 1.4% 1.4%
2013_Zou 37 40 30 40 1.23 [1.01; 1.51] 2.4% 2.4%
2013_Li 29 32 19 32 1.53 [1.12; 2.08] 1.0% 1.0%
2014_Zhang(1) 48 50 37 50 1.30 [1.09; 1.54] 3.2% 3.2%
2014_Zhang(2) 39 40 33 40 1.18 [1.02; 1.37] 4.3% 4.3%
2014_Zhi 44 54 40 54 1.10 [0.90; 1.35] 2.4% 2.4%
2014_Wang 57 60 43 52 1.15 [1.00; 1.32] 5.2% 5.2%
2015_Ma 64 68 48 64 1.25 [1.08; 1.46] 4.1% 4.1%
2015_Lin 26 29 24 29 1.08 [0.88; 1.33] 2.3% 2.3%
2016_Zhang(1) 37 40 29 40 1.28 [1.03; 1.57] 2.2% 2.2%
2017_Zhou 32 34 24 34 1.33 [1.06; 1.68] 1.8% 1.8%
2017_Xu 184 200 142 200 1.30 [1.18; 1.43] 10.2% 10.2%
2017_Sun 47 51 36 51 1.31 [1.07; 1.59] 2.6% 2.6%
2017_Cui 21 22 19 22 1.11 [0.91; 1.34] 2.7% 2.7%
2018_Zeng 39 43 33 43 1.18 [0.98; 1.43] 2.7% 2.7%
2019_Wang 39 40 29 40 1.34 [1.10; 1.64] 2.5% 2.5%
2020_Gong 46 50 36 50 1.28 [1.06; 1.55] 2.7% 2.7%
2021_Chen 46 50 39 50 1.18 [1.00; 1.40] 3.4% 3.4%
Common effect model 1148 1115 1.21 [1.17; 1.25] 73.3% --
Random effects model 1.21 [1.16; 1.26] -- 73.3%
Heterogeneity: I^2 = 0%, τ^2 = 0.0006, p = 0.60
team = TEAM
2006_Xu 33 35 26 35 1.27 [1.03; 1.57] 2.2% 2.2%
2010_Ma 38 40 30 40 1.27 [1.04; 1.54] 2.6% 2.6%
2011_Xu 28 30 21 30 1.33 [1.04; 1.72] 1.5% 1.5%
2013_Lu 36 39 28 37 1.22 [0.99; 1.50] 2.3% 2.3%
2013_Gao 30 32 24 31 1.21 [0.98; 1.49] 2.2% 2.2%
2017_Shao 26 29 24 29 1.08 [0.88; 1.33] 2.3% 2.3%
2019_Han 28 30 22 30 1.27 [1.01; 1.61] 1.7% 1.7%
2019_Jiang 65 74 55 74 1.18 [1.01; 1.38] 3.9% 3.9%
2020_Zhao(1) 33 35 26 35 1.27 [1.03; 1.57] 2.2% 2.2%
2024_Hu 52 54 45 54 1.16 [1.01; 1.32] 5.7% 5.7%
Common effect model 398 395 1.21 [1.14; 1.28] 26.7% --
Random effects model 1.21 [1.14; 1.28] -- 26.7%
Heterogeneity: I^2 = 0%, τ^2 = 0, p = 0.96
Common effect model 1546 1510 1.21 [1.17; 1.25] 100.0% --
Random effects model 1.21 [1.17; 1.25] -- 100.0%
Heterogeneity: I^2 = 0%, τ^2 = 0, p = 0.88
0.5 1 2
Test for subgroup differences (common effect): χ^2_1 = 0.00, df = 1 (p = 0.98)
Test for subgroup differences (random effects): χ^2_1 = 0.00, df = 1 (p = 0.99)

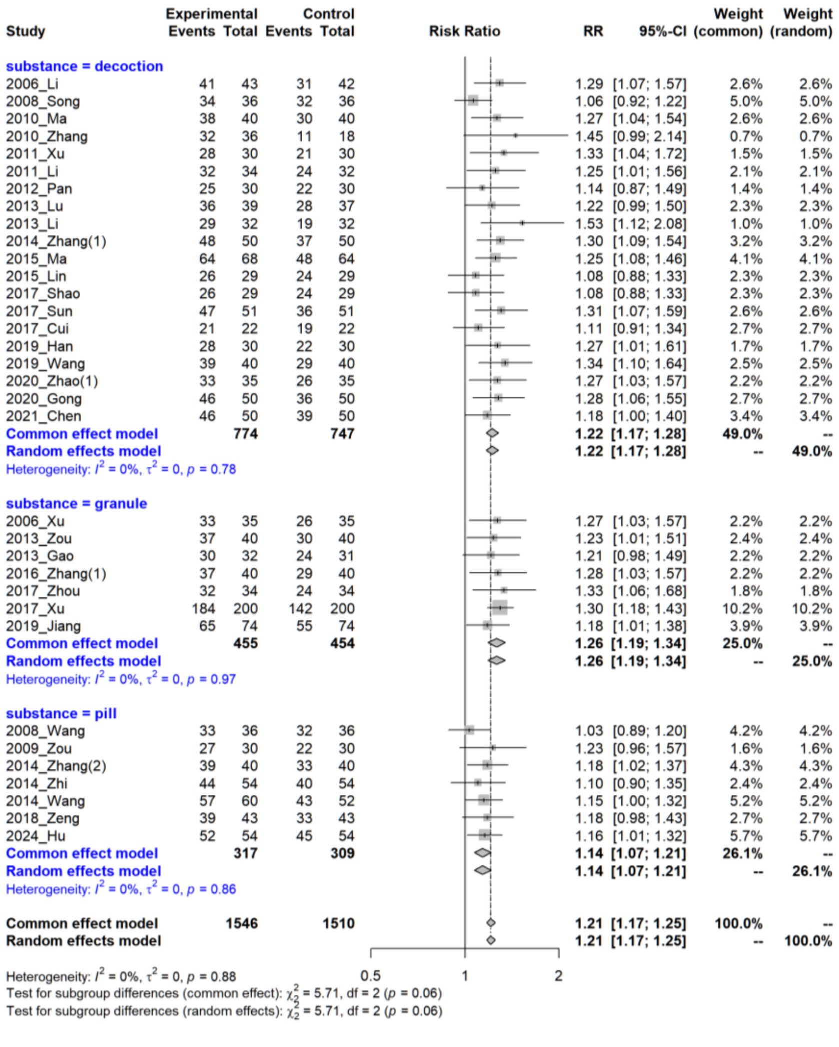
Experimental Control
Study Events Total Events Total Risk Ratio RR 95%-CI Weight (common) Weight (random)
substance = decoction
2006_Li 41 43 31 42 1.29 [1.07; 1.57] 2.6% 2.6%
2008_Song 34 36 32 36 1.06 [0.92; 1.22] 5.0% 5.0%
2010_Ma 38 40 30 40 1.27 [1.04; 1.54] 2.6% 2.6%
2010_Zhang 32 36 11 18 1.45 [0.99; 2.14] 0.7% 0.7%
2011_Xu 28 30 21 30 1.33 [1.04; 1.72] 1.5% 1.5%
2011_Li 32 34 24 32 1.25 [1.01; 1.56] 2.1% 2.1%
2012_Pan 25 30 22 30 1.14 [0.87; 1.49] 1.4% 1.4%
2013_Lu 36 39 28 37 1.22 [0.99; 1.50] 2.3% 2.3%
2013_Li 29 32 19 32 1.53 [1.12; 2.08] 1.0% 1.0%
2014_Zhang(1) 48 50 37 50 1.30 [1.09; 1.54] 3.2% 3.2%
2015_Ma 64 68 48 64 1.25 [1.08; 1.46] 4.1% 4.1%
2015_Lin 26 29 24 29 1.08 [0.88; 1.33] 2.3% 2.3%
2017_Shao 26 29 24 29 1.08 [0.88; 1.33] 2.3% 2.3%
2017_Sun 47 51 36 51 1.31 [1.07; 1.59] 2.6% 2.6%
2017_Cui 21 22 19 22 1.11 [0.91; 1.34] 2.7% 2.7%
2019_Han 28 30 22 30 1.27 [1.01; 1.61] 1.7% 1.7%
2019_Wang 39 40 29 40 1.34 [1.10; 1.64] 2.5% 2.5%
2020_Zhao(1) 33 35 26 35 1.27 [1.03; 1.57] 2.2% 2.2%
2020_Gong 46 50 36 50 1.28 [1.06; 1.55] 2.7% 2.7%
2021_Chen 46 50 39 50 1.18 [1.00; 1.40] 3.4% 3.4%
Common effect model 774 747 1.22 [1.17; 1.28] 49.0% --
Random effects model 1.22 [1.17; 1.28] -- 49.0%
Heterogeneity: I^2 = 0%, τ^2 = 0, p = 0.78
substance = granule
2006_Xu 33 35 26 35 1.27 [1.03; 1.57] 2.2% 2.2%
2013_Zou 37 40 30 40 1.23 [1.01; 1.51] 2.4% 2.4%
2013_Gao 30 32 24 31 1.21 [0.98; 1.49] 2.2% 2.2%
2016_Zhang(1) 37 40 29 40 1.28 [1.03; 1.57] 2.2% 2.2%
2017_Zhou 32 34 24 34 1.33 [1.06; 1.68] 1.8% 1.8%
2017_Xu 184 200 142 200 1.30 [1.18; 1.43] 10.2% 10.2%
2019_Jiang 65 74 55 74 1.18 [1.01; 1.38] 3.9% 3.9%
Common effect model 455 454 1.26 [1.19; 1.34] 25.0% --
Random effects model 1.26 [1.19; 1.34] -- 25.0%
Heterogeneity: I^2 = 0%, τ^2 = 0, p = 0.97
substance = pill
2008_Wang 33 36 32 36 1.03 [0.89; 1.20] 4.2% 4.2%
2009_Zou 27 30 22 30 1.23 [0.96; 1.57] 1.6% 1.6%
2014_Zhang(2) 39 40 33 40 1.18 [1.02; 1.37] 4.3% 4.3%
2014_Zhi 44 54 40 54 1.10 [0.90; 1.35] 2.4% 2.4%
2014_Wang 57 60 43 52 1.15 [1.00; 1.32] 5.2% 5.2%
2018_Zeng 39 43 33 43 1.18 [0.98; 1.43] 2.7% 2.7%
2024_Hu 52 54 45 54 1.16 [1.01; 1.32] 5.7% 5.7%
Common effect model 317 309 1.14 [1.07; 1.21] 26.1% --
Random effects model 1.14 [1.07; 1.21] -- 26.1%
Heterogeneity: I^2 = 0%, τ^2 = 0, p = 0.86
Common effect model 1546 1510 1.21 [1.17; 1.25] 100.0% --
Random effects model 1.21 [1.17; 1.25] -- 100.0%
Heterogeneity: I^2 = 0%, τ^2 = 0, p = 0.88
0.5 1 2
Test for subgroup differences (common effect): χ^2_2 = 5.71, df = 2 (p = 0.06)
Test for subgroup differences (random effects): χ^2_2 = 5.71, df = 2 (p = 0.06)

2.1.5

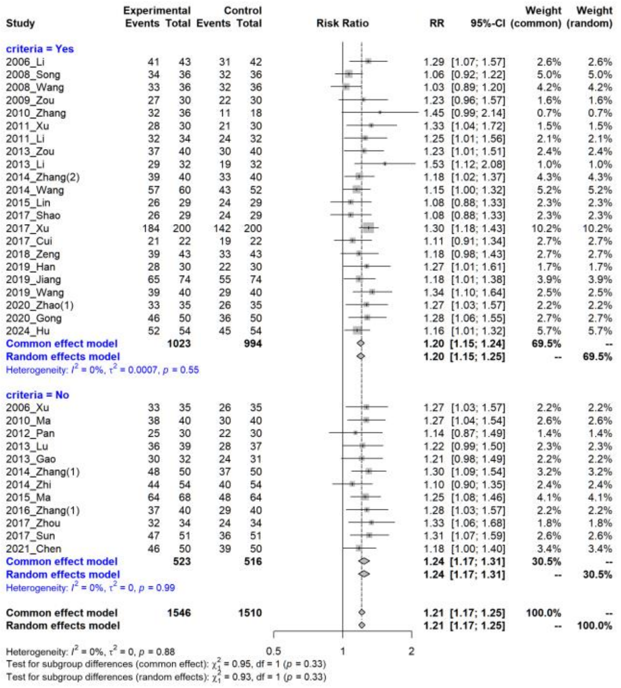
Study
Experimental Events Total
Control Events Total
Risk Ratio
RR 95%-CI
Weight (common)
Weight (random)
criteria = Yes
2006_Li 41 43 31 42 1.29 [1.07; 1.57] 2.6% 2.6%
2008_Song 34 36 32 36 1.06 [0.92; 1.22] 5.0% 5.0%
2008_Wang 33 36 32 36 1.03 [0.89; 1.20] 4.2% 4.2%
2009_Zou 27 30 22 30 1.23 [0.96; 1.57] 1.6% 1.6%
2010_Zhang 32 36 11 18 1.45 [0.99; 2.14] 0.7% 0.7%
2011_Xu 28 30 21 30 1.33 [1.04; 1.72] 1.5% 1.5%
2011_Li 32 34 24 32 1.25 [1.01; 1.56] 2.1% 2.1%
2013_Zou 37 40 30 40 1.23 [1.01; 1.51] 2.4% 2.4%
2013_Li 29 32 19 32 1.53 [1.12; 2.08] 1.0% 1.0%
2014_Zhang(2) 39 40 33 40 1.18 [1.02; 1.37] 4.3% 4.3%
2014_Wang 57 60 43 52 1.15 [1.00; 1.32] 5.2% 5.2%
2015_Lin 26 29 24 29 1.08 [0.88; 1.33] 2.3% 2.3%
2017_Shao 26 29 24 29 1.08 [0.88; 1.33] 2.3% 2.3%
2017_Xu 184 200 142 200 1.30 [1.18; 1.43] 10.2% 10.2%
2017_Cui 21 22 19 22 1.11 [0.91; 1.34] 2.7% 2.7%
2018_Zeng 39 43 33 43 1.18 [0.98; 1.43] 2.7% 2.7%
2019_Han 28 30 22 30 1.27 [1.01; 1.61] 1.7% 1.7%
2019_Jiang 65 74 55 74 1.18 [1.01; 1.38] 3.9% 3.9%
2019_Wang 39 40 29 40 1.34 [1.10; 1.64] 2.5% 2.5%
2020_Zhao(1) 33 35 26 35 1.27 [1.03; 1.57] 2.2% 2.2%
2020_Gong 46 50 36 50 1.28 [1.06; 1.55] 2.7% 2.7%
2024_Hu 52 54 45 54 1.16 [1.01; 1.32] 5.7% 5.7%
Common effect model 1023 994 1.20 [1.15; 1.24] 69.5% --
Random effects model 1.20 [1.15; 1.25] -- 69.5%
Heterogeneity: I^2 = 0%, τ^2 = 0.0007, p = 0.55
criteria = No
2006_Xu 33 35 26 35 1.27 [1.03; 1.57] 2.2% 2.2%
2010_Ma 38 40 30 40 1.27 [1.04; 1.54] 2.6% 2.6%
2012_Pan 25 30 22 30 1.14 [0.87; 1.49] 1.4% 1.4%
2013_Lu 36 39 28 37 1.22 [0.99; 1.50] 2.3% 2.3%
2013_Gao 30 32 24 31 1.21 [0.98; 1.49] 2.2% 2.2%
2014_Zhang(1) 48 50 37 50 1.30 [1.09; 1.54] 3.2% 3.2%
2014_Zhi 44 54 40 54 1.10 [0.90; 1.35] 2.4% 2.4%
2015_Ma 64 68 48 64 1.25 [1.08; 1.46] 4.1% 4.1%
2016_Zhang(1) 37 40 29 40 1.28 [1.03; 1.57] 2.2% 2.2%
2017_Zhou 32 34 24 34 1.33 [1.06; 1.68] 1.8% 1.8%
2017_Sun 47 51 36 51 1.31 [1.07; 1.59] 2.6% 2.6%
2021_Chen 46 50 39 50 1.18 [1.00; 1.40] 3.4% 3.4%
Common effect model 523 516 1.24 [1.17; 1.31] 30.5% --
Random effects model 1.24 [1.17; 1.31] -- 30.5%
Heterogeneity: I^2 = 0%, τ^2 = 0, p = 0.99
Common effect model 1546 1510 1.21 [1.17; 1.25] 100.0% --
Random effects model 1.21 [1.17; 1.25] -- 100.0%
0.5
1
2
Heterogeneity: I^2 = 0%, τ^2 = 0, p = 0.88
Test for subgroup differences (common effect): χ^2_1 = 0.95, df = 1 (p = 0.33)
Test for subgroup differences (random effects): χ^2_1 = 0.93, df = 1 (p = 0.33)

2.1.6

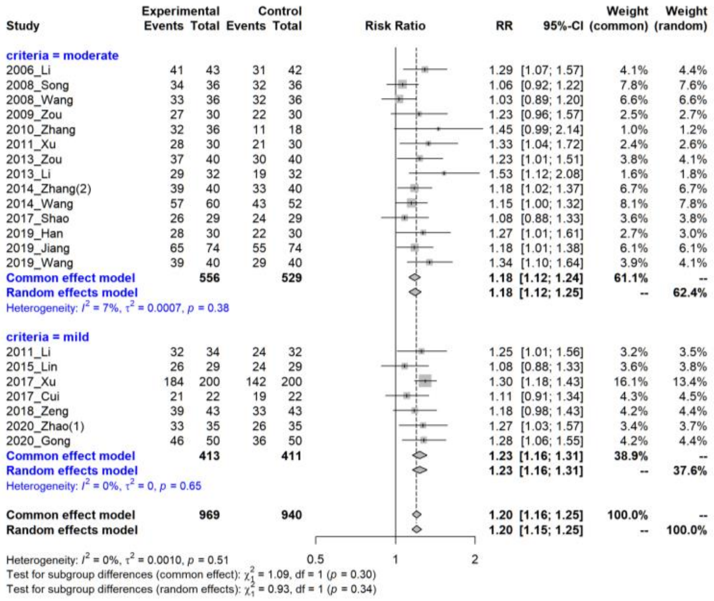
Study
Experimental Events Total
Control Events Total
Risk Ratio
RR 95%-CI
Weight (common)
Weight (random)
criteria = moderate
2006_Li 41 43 31 42 1.29 [1.07; 1.57] 4.1% 4.4%
2008_Song 34 36 32 36 1.06 [0.92; 1.22] 7.8% 7.6%
2008_Wang 33 36 32 36 1.03 [0.89; 1.20] 6.6% 6.6%
2009_Zou 27 30 22 30 1.23 [0.96; 1.57] 2.5% 2.7%
2010_Zhang 32 36 11 18 1.45 [0.99; 2.14] 1.0% 1.2%
2011_Xu 28 30 21 30 1.33 [1.04; 1.72] 2.4% 2.6%
2013_Zou 37 40 30 40 1.23 [1.01; 1.51] 3.8% 4.1%
2013_Li 29 32 19 32 1.53 [1.12; 2.08] 1.6% 1.8%
2014_Zhang(2) 39 40 33 40 1.18 [1.02; 1.37] 6.7% 6.7%
2014_Wang 57 60 43 52 1.15 [1.00; 1.32] 8.1% 7.8%
2017_Shao 26 29 24 29 1.08 [0.88; 1.33] 3.6% 3.8%
2019_Han 28 30 22 30 1.27 [1.01; 1.61] 2.7% 3.0%
2019_Jiang 65 74 55 74 1.18 [1.01; 1.38] 6.1% 6.1%
2019_Wang 39 40 29 40 1.34 [1.10; 1.64] 3.9% 4.1%
Common effect model 556 529 1.18 [1.12; 1.24] 61.1% --
Random effects model 1.18 [1.12; 1.25] -- 62.4%
Heterogeneity: I^2 = 7%, τ^2 = 0.0007, p = 0.38
criteria = mild
2011_Li 32 34 24 32 1.25 [1.01; 1.56] 3.2% 3.5%
2015_Lin 26 29 24 29 1.08 [0.88; 1.33] 3.6% 3.8%
2017_Xu 184 200 142 200 1.30 [1.18; 1.43] 16.1% 13.4%
2017_Cui 21 22 19 22 1.11 [0.91; 1.34] 4.3% 4.5%
2018_Zeng 39 43 33 43 1.18 [0.98; 1.43] 4.2% 4.4%
2020_Zhao(1) 33 35 26 35 1.27 [1.03; 1.57] 3.4% 3.7%
2020_Gong 46 50 36 50 1.28 [1.06; 1.55] 4.2% 4.4%
Common effect model 413 411 1.23 [1.16; 1.31] 38.9% --
Random effects model 1.23 [1.16; 1.31] -- 37.6%
Heterogeneity: I^2 = 0%, τ^2 = 0, p = 0.65
Common effect model 969 940 1.20 [1.16; 1.25] 100.0% --
Random effects model 1.20 [1.15; 1.25] -- 100.0%
0.5
1
2
Heterogeneity: I^2 = 0%, τ^2 = 0.0010, p = 0.51
Test for subgroup differences (common effect): χ^2_1 = 1.09, df = 1 (p = 0.30)
Test for subgroup differences (random effects): χ^2_1 = 0.93, df = 1 (p = 0.34)

2.1.7

Study	Experimental Total	Experimental Mean	Experimental SD	Control Total	Control Mean	Control SD	MD	95%-CI	Weight (common)	Weight (random)
**med = SSRI**										
2006_Li	43	66	22	42	46	19	20.02	[11.30; 28.74]	0.4%	11.3%
2008_Song	36	64	8	36	62	7	2.23	[-1.38; 5.84]	2.6%	14.2%
2016_Zhang(2)	46	86	6	46	62	5	24.40	[22.24; 26.56]	7.3%	14.7%
2017_Cui	22	56	20	22	52	20	3.86	[-7.84; 15.56]	0.2%	9.4%
2020_Zhao(2)	69	82	2	69	73	2	8.80	[8.16; 9.44]	84.1%	15.0%
2021_Zheng	25	66	24	25	47	18	19.22	[7.66; 30.78]	0.3%	9.5%
**Common effect model**	**241**			**240**			**9.88**	**[9.28; 10.48]**	**94.9%**	**--**
**Random effects model**							**13.07**	**[5.33; 20.81]**	**--**	**74.3%**
Heterogeneity: $I^2 = 98\%$, $\tau^2 = 80.0623$, $p < 0.01$										
**med = TCA**										
2016_Zhang(1)	40	67	22	40	47	19	20.02	[10.98; 29.06]	0.4%	11.1%
2019_Han	30	68	3	30	53	7	15.03	[12.33; 17.73]	4.7%	14.6%
**Common effect model**	**70**			**70**			**15.44**	**[12.85; 18.02]**	**5.1%**	**--**
**Random effects model**							**15.58**	**[12.51; 18.65]**	**--**	**25.7%**
Heterogeneity: $I^2 = 7\%$, $\tau^2 = 0.8596$, $p = 0.30$										
**Common effect model**	**311**			**310**			**10.17**	**[9.58; 10.75]**	**100.0%**	**--**
**Random effects model**							**14.12**	**[8.22; 20.01]**	**--**	**100.0%**

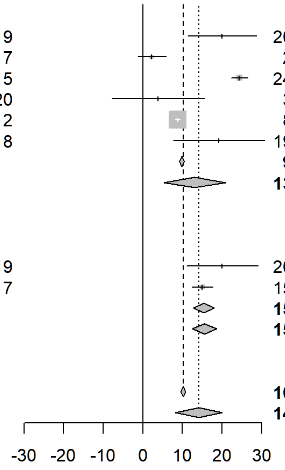

Heterogeneity: $I^2 = 97\%$, $\tau^2 = 60.0738$, $p < 0.01$
Test for subgroup differences (common effect): $\chi^2_1 = 16.83$, df = 1 ($p < 0.01$)
Test for subgroup differences (random effects): $\chi^2_1 = 0.35$, df = 1 ($p = 0.55$)

Study	Experimental Total	Experimental Mean	Experimental SD	Control Total	Control Mean	Control SD	MD	95%-CI	Weight (common)	Weight (random)
**period = More than 4 weeks**										
2006_Li	43	66	22	42	46	19	20.02	[11.30; 28.74]	0.4%	11.3%
2008_Song	36	64	8	36	62	7	2.23	[-1.38; 5.84]	2.6%	14.2%
2016_Zhang(1)	40	67	22	40	47	19	20.02	[10.98; 29.06]	0.4%	11.1%
2020_Zhao(2)	69	82	2	69	73	2	8.80	[8.16; 9.44]	84.1%	15.0%
2021_Zheng	25	66	24	25	47	18	19.22	[7.66; 30.78]	0.3%	9.5%
**Common effect model**	**213**			**212**			**8.75**	**[8.12; 9.37]**	**87.8%**	**--**
**Random effects model**							**12.99**	**[5.50; 20.48]**	**--**	**61.2%**
Heterogeneity: $I^2 = 86\%$, $\tau^2 = 59.2419$, $p < 0.01$										
**period = 4 weeks or less**										
2016_Zhang(2)	46	86	6	46	62	5	24.40	[22.24; 26.56]	7.3%	14.7%
2017_Cui	22	56	20	22	52	20	3.86	[-7.84; 15.56]	0.2%	9.4%
2019_Han	30	68	3	30	53	7	15.03	[12.33; 17.73]	4.7%	14.6%
**Common effect model**	**98**			**98**			**20.39**	**[18.72; 22.06]**	**12.2%**	**--**
**Random effects model**							**15.58**	**[4.75; 26.42]**	**--**	**38.8%**
Heterogeneity: $I^2 = 94\%$, $\tau^2 = 81.1553$, $p < 0.01$										
**Common effect model**	**311**			**310**			**10.17**	**[9.58; 10.75]**	**100.0%**	**--**
**Random effects model**							**14.12**	**[8.22; 20.01]**	**--**	**100.0%**

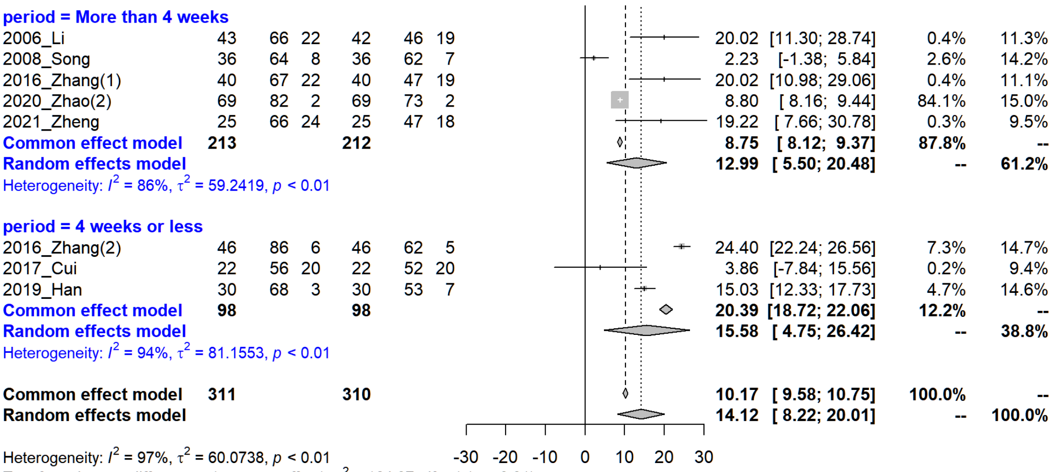

Heterogeneity: $I^2 = 97\%$, $\tau^2 = 60.0738$, $p < 0.01$
Test for subgroup differences (common effect): $\chi^2_1 = 164.07$, df = 1 ($p < 0.01$)
Test for subgroup differences (random effects): $\chi^2_1 = 0.15$, df = 1 ($p = 0.70$)

Study	Experimental Total	Experimental Mean	Experimental SD	Control Total	Control Mean	Control SD	MD	95%-CI	Weight (common)	Weight (random)
**team = None**										
2006_Li	43	66	22	42	46	19	20.02	[11.30; 28.74]	0.4%	11.3%
2008_Song	36	64	8	36	62	7	2.23	[-1.38; 5.84]	2.6%	14.2%
2016_Zhang(1)	40	67	22	40	47	19	20.02	[10.98; 29.06]	0.4%	11.1%
2016_Zhang(2)	46	86	6	46	62	5	24.40	[22.24; 26.56]	7.3%	14.7%
2017_Cui	22	56	20	22	52	20	3.86	[-7.84; 15.56]	0.2%	9.4%
**Common effect model**	**187**			**186**			**18.33**	**[16.57; 20.09]**	**11.0%**	**--**
**Random effects model**							**14.33**	**[5.02; 23.63]**	**--**	**60.9%**
Heterogeneity: $I^2 = 96\%$, $\tau^2 = 97.8810$, $p < 0.01$										
**team = TEAM**										
2019_Han	30	68	3	30	53	7	15.03	[12.33; 17.73]	4.7%	14.6%
2020_Zhao(2)	69	82	2	69	73	2	8.80	[8.16; 9.44]	84.1%	15.0%
2021_Zheng	25	66	24	25	47	18	19.22	[7.66; 30.78]	0.3%	9.5%
**Common effect model**	**124**			**124**			**9.16**	**[8.54; 9.77]**	**89.0%**	**--**
**Random effects model**							**12.91**	**[7.34; 18.48]**	**--**	**39.1%**
Heterogeneity: $I^2 = 91\%$, $\tau^2 = 18.1010$, $p < 0.01$										
**Common effect model**	**311**			**310**			**10.17**	**[9.58; 10.75]**	**100.0%**	**--**
**Random effects model**							**14.12**	**[8.22; 20.01]**	**--**	**100.0%**

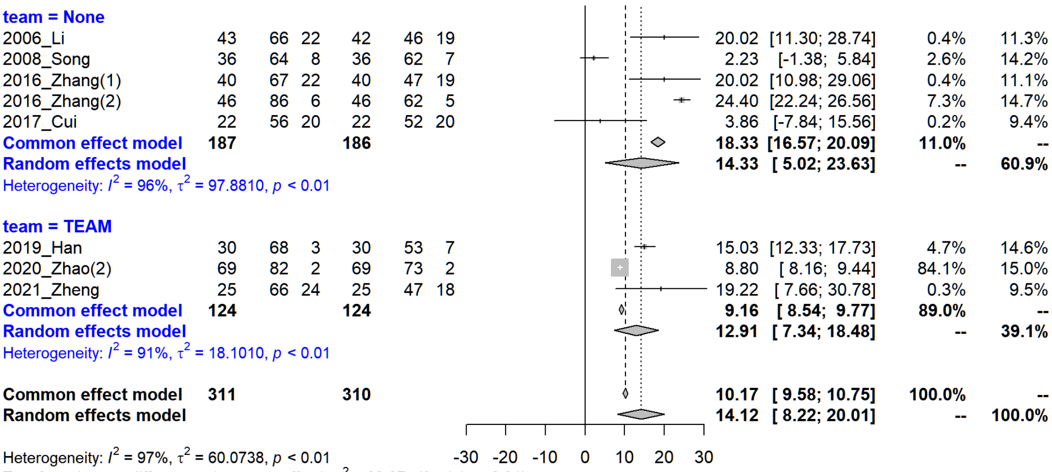

Heterogeneity: $I^2 = 97\%$, $\tau^2 = 60.0738$, $p < 0.01$
Test for subgroup differences (common effect): $\chi^2_1 = 93.07$, df = 1 ($p < 0.01$)
Test for subgroup differences (random effects): $\chi^2_1 = 0.07$, df = 1 ($p = 0.80$)

Study	Experimental Total	Experimental Mean	Experimental SD	Control Total	Control Mean	Control SD	MD	95%-CI	Weight (common)	Weight (random)
**substance = decoction**										
2006_Li	43	66	22	42	46	19	20.02	[11.30; 28.74]	0.4%	11.3%
2008_Song	36	64	8	36	62	7	2.23	[-1.38; 5.84]	2.6%	14.2%
2016_Zhang(2)	46	86	6	46	62	5	24.40	[22.24; 26.56]	7.3%	14.7%
2017_Cui	22	56	20	22	52	20	3.86	[-7.84; 15.56]	0.2%	9.4%
2019_Han	30	68	3	30	53	7	15.03	[12.33; 17.73]	4.7%	14.6%
2020_Zhao(2)	69	82	2	69	73	2	8.80	[8.16; 9.44]	84.1%	15.0%
2021_Zheng	25	66	24	25	47	18	19.22	[7.66; 30.78]	0.3%	9.5%
**Common effect model**	**271**			**270**			**10.12**	**[9.54; 10.71]**	**99.6%**	**--**
**Random effects model**							**13.38**	**[6.91; 19.85]**	**--**	**88.9%**
Heterogeneity: $I^2 = 97\%$, $\tau^2 = 65.0510$, $p < 0.01$										
**substance = granule**										
2016_Zhang(1)	40	67	22	40	47	19	20.02	[10.98; 29.06]	0.4%	11.1%
**Common effect model**	**311**			**310**			**10.17**	**[9.58; 10.75]**	**100.0%**	**--**
**Random effects model**							**14.12**	**[8.22; 20.01]**	**--**	**100.0%**

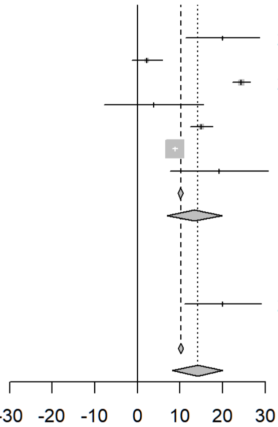

Heterogeneity: $I^2 = 97\%$, $\tau^2 = 60.0738$, $p < 0.01$
Test for subgroup differences (common effect): $\chi^2_1 = 4.58$, df = 1 ($p = 0.03$)
Test for subgroup differences (random effects): $\chi^2_1 = 1.37$, df = 1 ($p = 0.24$)

Study	Experimental Total	Experimental Mean	Experimental SD	Control Total	Control Mean	Control SD	MD	95%-CI	Weight (common)	Weight (random)
**criteria = Yes**										
2006_Li	43	66	22	42	46	19	20.02	[11.30; 28.74]	0.4%	11.3%
2008_Song	36	64	8	36	62	7	2.23	[-1.38; 5.84]	2.6%	14.2%
2016_Zhang(2)	46	86	6	46	62	5	24.40	[22.24; 26.56]	7.3%	14.7%
2017_Cui	22	56	20	22	52	20	3.86	[-7.84; 15.56]	0.2%	9.4%
2019_Han	30	68	3	30	53	7	15.03	[12.33; 17.73]	4.7%	14.6%
2020_Zhao(2)	69	82	2	69	73	2	8.80	[8.16; 9.44]	84.1%	15.0%
**Common effect model**	**246**			**245**			**10.10**	**[9.52; 10.69]**	**99.3%**	**--**
**Random effects model**							**12.65**	**[5.47; 19.83]**	**--**	**79.4%**
Heterogeneity: $I^2 = 98\%$, $\tau^2 = 72.0095$, $p < 0.01$										
**criteria = No**										
2016_Zhang(1)	40	67	22	40	47	19	20.02	[10.98; 29.06]	0.4%	11.1%
2021_Zheng	25	66	24	25	47	18	19.22	[7.66; 30.78]	0.3%	9.5%
**Common effect model**	**65**			**65**			**19.72**	**[12.59; 26.84]**	**0.7%**	**--**
**Random effects model**							**19.72**	**[12.59; 26.84]**	**--**	**20.6%**
Heterogeneity: $I^2 = 0\%$, $\tau^2 = 0$, $p = 0.91$										
**Common effect model**	**311**			**310**			**10.17**	**[9.58; 10.75]**	**100.0%**	**--**
**Random effects model**							**14.12**	**[8.22; 20.01]**	**--**	**100.0%**

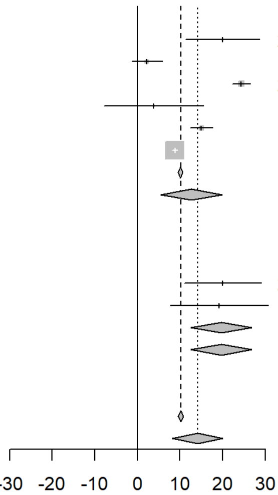

Heterogeneity: $I^2 = 97\%$, $\tau^2 = 60.0738$, $p < 0.01$
Test for subgroup differences (common effect): $\chi^2_1 = 6.95$, df = 1 ($p < 0.01$)
Test for subgroup differences (random effects): $\chi^2_1 = 1.88$, df = 1 ($p = 0.17$)

2.1.8

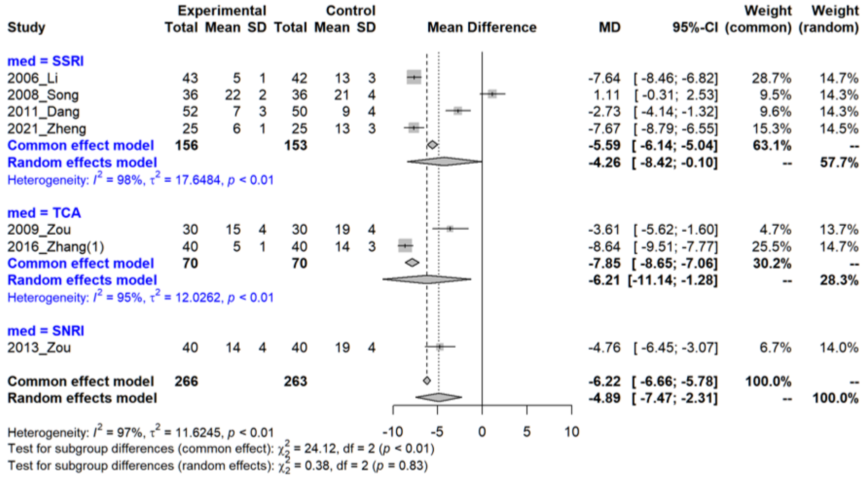

Study | Experimental Total Mean SD | Control Total Mean SD | Mean Difference | MD | 95%-CI | Weight (common) | Weight (random)
med = SSRI
2006_Li 43 5 1 42 13 3 -7.64 [-8.46; -6.82] 28.7% 14.7%
2008_Song 36 22 2 36 21 4 1.11 [-0.31; 2.53] 9.5% 14.3%
2011_Dang 52 7 3 50 9 4 -2.73 [-4.14; -1.32] 9.6% 14.3%
2021_Zheng 25 6 1 25 13 3 -7.67 [-8.79; -6.55] 15.3% 14.5%
Common effect model 156 153 -5.59 [-6.14; -5.04] 63.1% --
Random effects model -4.26 [-8.42; -0.10] -- 57.7%
Heterogeneity: I^2 = 98%, τ^2 = 17.6484, p < 0.01
med = TCA
2009_Zou 30 15 4 30 19 4 -3.61 [-5.62; -1.60] 4.7% 13.7%
2016_Zhang(1) 40 5 1 40 14 3 -8.64 [-9.51; -7.77] 25.5% 14.7%
Common effect model 70 70 -7.85 [-8.65; -7.06] 30.2% --
Random effects model -6.21 [-11.14; -1.28] -- 28.3%
Heterogeneity: I^2 = 95%, τ^2 = 12.0262, p < 0.01
med = SNRI
2013_Zou 40 14 4 40 19 4 -4.76 [-6.45; -3.07] 6.7% 14.0%
Common effect model 266 263 -6.22 [-6.66; -5.78] 100.0% --
Random effects model -4.89 [-7.47; -2.31] -- 100.0%
-10 -5 0 5 10
Heterogeneity: I^2 = 97%, τ^2 = 11.6245, p < 0.01
Test for subgroup differences (common effect): χ^2_2 = 24.12, df = 2 (p < 0.01)
Test for subgroup differences (random effects): χ^2_2 = 0.38, df = 2 (p = 0.83)

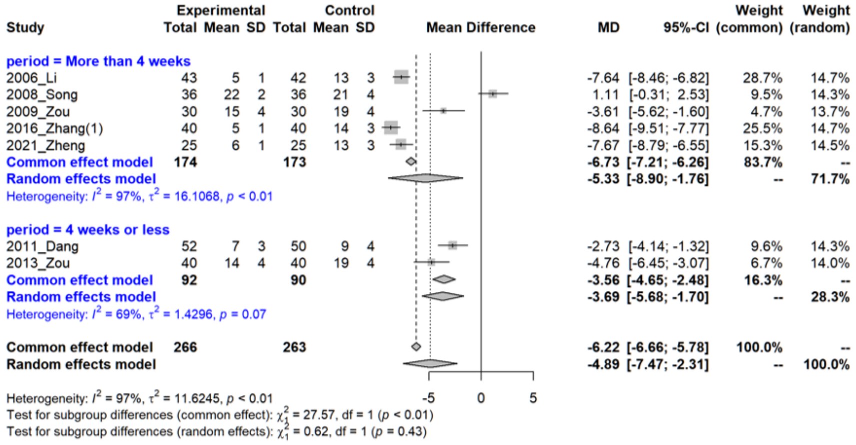

Study | Experimental Total Mean SD | Control Total Mean SD | Mean Difference | MD | 95%-CI | Weight (common) | Weight (random)
period = More than 4 weeks
2006_Li 43 5 1 42 13 3 -7.64 [-8.46; -6.82] 28.7% 14.7%
2008_Song 36 22 2 36 21 4 1.11 [-0.31; 2.53] 9.5% 14.3%
2009_Zou 30 15 4 30 19 4 -3.61 [-5.62; -1.60] 4.7% 13.7%
2016_Zhang(1) 40 5 1 40 14 3 -8.64 [-9.51; -7.77] 25.5% 14.7%
2021_Zheng 25 6 1 25 13 3 -7.67 [-8.79; -6.55] 15.3% 14.5%
Common effect model 174 173 -6.73 [-7.21; -6.26] 83.7% --
Random effects model -5.33 [-8.90; -1.76] -- 71.7%
Heterogeneity: I^2 = 97%, τ^2 = 16.1068, p < 0.01
period = 4 weeks or less
2011_Dang 52 7 3 50 9 4 -2.73 [-4.14; -1.32] 9.6% 14.3%
2013_Zou 40 14 4 40 19 4 -4.76 [-6.45; -3.07] 6.7% 14.0%
Common effect model 92 90 -3.56 [-4.65; -2.48] 16.3% --
Random effects model -3.69 [-5.68; -1.70] -- 28.3%
Heterogeneity: I^2 = 69%, τ^2 = 1.4296, p = 0.07
Common effect model 266 263 -6.22 [-6.66; -5.78] 100.0% --
Random effects model -4.89 [-7.47; -2.31] -- 100.0%
-5 0 5
Heterogeneity: I^2 = 97%, τ^2 = 11.6245, p < 0.01
Test for subgroup differences (common effect): χ^2_1 = 27.57, df = 1 (p < 0.01)
Test for subgroup differences (random effects): χ^2_1 = 0.62, df = 1 (p = 0.43)

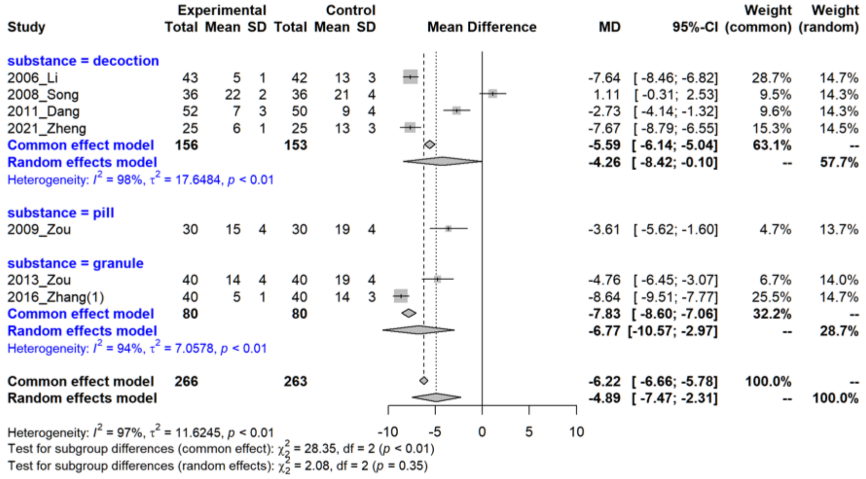

Study | Experimental Total Mean SD | Control Total Mean SD | Mean Difference | MD | 95%-CI | Weight (common) | Weight (random)
substance = decoction
2006_Li 43 5 1 42 13 3 -7.64 [-8.46; -6.82] 28.7% 14.7%
2008_Song 36 22 2 36 21 4 1.11 [-0.31; 2.53] 9.5% 14.3%
2011_Dang 52 7 3 50 9 4 -2.73 [-4.14; -1.32] 9.6% 14.3%
2021_Zheng 25 6 1 25 13 3 -7.67 [-8.79; -6.55] 15.3% 14.5%
Common effect model 156 153 -5.59 [-6.14; -5.04] 63.1% --
Random effects model -4.26 [-8.42; -0.10] -- 57.7%
Heterogeneity: I^2 = 98%, τ^2 = 17.6484, p < 0.01
substance = pill
2009_Zou 30 15 4 30 19 4 -3.61 [-5.62; -1.60] 4.7% 13.7%
substance = granule
2013_Zou 40 14 4 40 19 4 -4.76 [-6.45; -3.07] 6.7% 14.0%
2016_Zhang(1) 40 5 1 40 14 3 -8.64 [-9.51; -7.77] 25.5% 14.7%
Common effect model 80 80 -7.83 [-8.60; -7.06] 32.2% --
Random effects model -6.77 [-10.57; -2.97] -- 28.7%
Heterogeneity: I^2 = 94%, τ^2 = 7.0578, p < 0.01
Common effect model 266 263 -6.22 [-6.66; -5.78] 100.0% --
Random effects model -4.89 [-7.47; -2.31] -- 100.0%
-10 -5 0 5 10
Heterogeneity: I^2 = 97%, τ^2 = 11.6245, p < 0.01
Test for subgroup differences (common effect): χ^2_2 = 28.35, df = 2 (p < 0.01)
Test for subgroup differences (random effects): χ^2_2 = 2.08, df = 2 (p = 0.35)

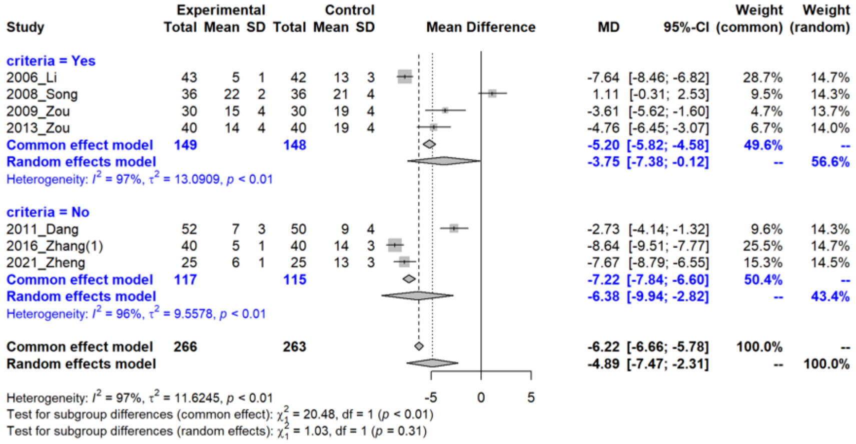

Study | Experimental Total Mean SD | Control Total Mean SD | Mean Difference | MD | 95%-CI | Weight (common) | Weight (random)
criteria = Yes
2006_Li 43 5 1 42 13 3 -7.64 [-8.46; -6.82] 28.7% 14.7%
2008_Song 36 22 2 36 21 4 1.11 [-0.31; 2.53] 9.5% 14.3%
2009_Zou 30 15 4 30 19 4 -3.61 [-5.62; -1.60] 4.7% 13.7%
2013_Zou 40 14 4 40 19 4 -4.76 [-6.45; -3.07] 6.7% 14.0%
Common effect model 149 148 -5.20 [-5.82; -4.58] 49.6% --
Random effects model -3.75 [-7.38; -0.12] -- 56.6%
Heterogeneity: I^2 = 97%, τ^2 = 13.0909, p < 0.01
criteria = No
2011_Dang 52 7 3 50 9 4 -2.73 [-4.14; -1.32] 9.6% 14.3%
2016_Zhang(1) 40 5 1 40 14 3 -8.64 [-9.51; -7.77] 25.5% 14.7%
2021_Zheng 25 6 1 25 13 3 -7.67 [-8.79; -6.55] 15.3% 14.5%
Common effect model 117 115 -7.22 [-7.84; -6.60] 50.4% --
Random effects model -6.38 [-9.94; -2.82] -- 43.4%
Heterogeneity: I^2 = 96%, τ^2 = 9.5578, p < 0.01
Common effect model 266 263 -6.22 [-6.66; -5.78] 100.0% --
Random effects model -4.89 [-7.47; -2.31] -- 100.0%
-5 0 5
Heterogeneity: I^2 = 97%, τ^2 = 11.6245, p < 0.01
Test for subgroup differences (common effect): χ^2_1 = 20.48, df = 1 (p < 0.01)
Test for subgroup differences (random effects): χ^2_1 = 1.03, df = 1 (p = 0.31)

Supplementary Figure 2-2. Outcomes other than HAMD, TER(RR)

2.2.1 Odds ratio of TER

2.2.2 Mean difference of 5-HT

2.2.3 Standardized Mean difference of 5-HT

2.2.4 Mean difference of FMA

2.2.5 Mean difference of MBI

2.2.6 Mean difference of MMSE

2.2.7 Mean difference of NIHSS

2.2.8 Mean difference of PSQI

2.2.9 Mean difference of SSS

Study	Experimental Events	Experimental Total	Control Events	Control Total	OR	95%-CI	Weight (common)	Weight (random)
2006_Li	41	43	31	42	7.27	[1.50; 35.22]	2.1%	2.1%
2006_Xu	33	35	26	35	5.71	[1.13; 28.75]	2.0%	2.0%
2008_Song	34	36	32	36	2.12	[0.36; 12.41]	1.6%	1.6%
2008_Wang	33	36	32	36	1.38	[0.28; 6.64]	2.1%	2.1%
2009_Zou	27	30	22	30	3.27	[0.77; 13.83]	2.5%	2.5%
2010_Ma	38	40	30	40	6.33	[1.29; 31.11]	2.0%	2.0%
2010_Zhang	32	36	11	18	5.09	[1.25; 20.78]	2.6%	2.6%
2011_Xu	28	30	21	30	6.00	[1.17; 30.72]	1.9%	1.9%
2011_Li	32	34	24	32	5.33	[1.04; 27.42]	1.9%	1.9%
2012_Pan	25	30	22	30	1.82	[0.52; 6.38]	3.3%	3.3%
2013_Zou	37	40	30	40	4.11	[1.04; 16.29]	2.7%	2.7%
2013_Lu	36	39	28	37	3.86	[0.95; 15.59]	2.6%	2.6%
2013_Li	29	32	19	32	6.61	[1.66; 26.35]	2.7%	2.7%
2013_Gao	30	32	24	31	4.38	[0.83; 23.02]	1.9%	1.9%
2014_Zhang(1)	48	50	37	50	8.43	[1.79; 39.70]	2.1%	2.1%
2014_Zhang(2)	39	40	33	40	8.27	[0.97; 70.73]	1.1%	1.1%
2014_Zhi	44	54	40	54	1.54	[0.62; 3.85]	6.1%	6.1%
2014_Wang	57	60	43	52	3.98	[1.02; 15.58]	2.8%	2.8%
2015_Ma	64	68	48	64	5.33	[1.68; 16.98]	3.8%	3.8%
2015_Lin	26	29	24	29	1.81	[0.39; 8.38]	2.2%	2.2%
2016_Zhang(1)	37	40	29	40	4.68	[1.19; 18.34]	2.7%	2.7%
2017_Shao	26	29	24	29	1.81	[0.39; 8.38]	2.2%	2.2%
2017_Zhou	32	34	24	34	6.67	[1.34; 33.28]	2.0%	2.0%
2017_Xu	184	200	142	200	4.70	[2.59; 8.52]	14.5%	14.5%
2017_Sun	47	51	36	51	4.90	[1.50; 16.02]	3.7%	3.7%
2017_Cui	21	22	19	22	3.32	[0.32; 34.65]	0.9%	0.9%
2018_Zeng	39	43	33	43	2.95	[0.85; 10.30]	3.3%	3.3%
2019_Han	28	30	22	30	5.09	[0.98; 26.43]	1.9%	1.9%
2019_Jiang	65	74	55	74	2.49	[1.04; 5.96]	6.8%	6.8%
2019_Wang	39	40	29	40	14.79	[1.81; 121.14]	1.2%	1.2%
2020_Zhao(1)	33	35	26	35	5.71	[1.13; 28.75]	2.0%	2.0%
2020_Gong	46	50	36	50	4.47	[1.36; 14.76]	3.6%	3.6%
2021_Chen	46	50	39	50	3.24	[0.96; 11.00]	3.4%	3.4%
2024_Hu	52	54	45	54	5.20	[1.07; 25.33]	2.0%	2.0%
**Fixed effect model $p < 0.001$**		**1546**		**1510**	**3.89**	**[3.10; 4.88]**	**100.0%**	.
**Random effects model $p < 0.001$**					**3.89**	**[3.10; 4.88]**	.	**100.0%**

Heterogeneity: $I^2 = 0.0\%$, $\tau^2 = 0$, $p = 0.9853$

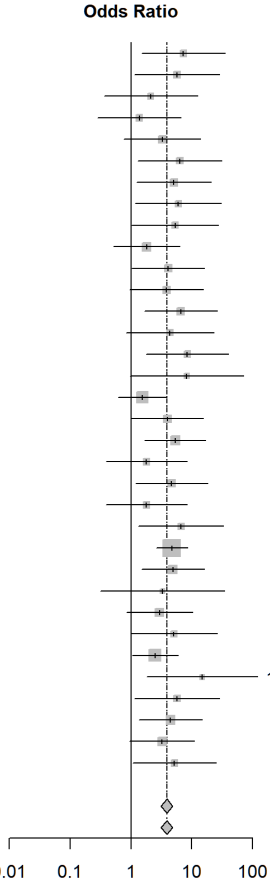

Study	Experimental Total	Experimental Mean	Experimental SD	Control Total	Control Mean	Control SD	MD	95%-CI	Weight (common)	Weight (random)
2018_Zeng	43	1462.90	67.80	43	1218.20	47.30	244.70	[219.99; 269.41]	4.6%	16.8%
2019_Han	30	395.17	10.45	30	316.07	14.42	79.10	[72.73; 85.47]	68.7%	17.4%
2019_Jiang	74	512.86	102.73	74	432.37	67.41	80.49	[52.49; 108.49]	3.6%	16.7%
2019_Wang	40	657.40	133.20	40	602.30	128.00	55.10	[-2.15; 112.35]	0.9%	14.8%
2020_Zhao(1)	35	395.23	32.76	35	328.45	31.97	66.78	[51.62; 81.94]	12.1%	17.2%
2021_Zheng	25	348.86	31.86	25	246.67	27.87	102.19	[85.60; 118.78]	10.1%	17.1%
**Fixed effect model $p < 0.001$**	**247**			**247**			**87.36**	**[82.08; 92.65]**	**100.0%**	.
**Random effects model $p < 0.001$**							**105.53**	**[49.19; 161.86]**	.	**100.0%**

Heterogeneity: $I^2 = 97.1\%$, $\tau^2 = 4746.2597$, $p < 0.0001$

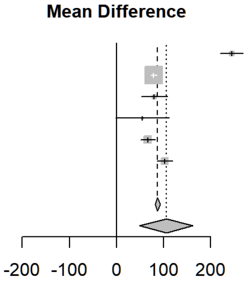

Study	Experimental Total	Experimental Mean	Experimental SD	Control Total	Control Mean	Control SD	SMD	95%-CI	Weight (common)	Weight (random)
2018_Zeng	43	1462.90	67.80	43	1218.20	47.30	4.15	[3.39; 4.91]	8.5%	16.7%
2019_Han	30	395.17	10.45	30	316.07	14.42	6.20	[4.95; 7.45]	3.1%	15.8%
2019_Jiang	74	512.86	102.73	74	432.37	67.41	0.92	[0.58; 1.26]	42.7%	17.1%
2019_Wang	40	657.40	133.20	40	602.30	128.00	0.42	[-0.03; 0.86]	25.0%	17.0%
2020_Zhao(1)	35	395.23	32.76	35	328.45	31.97	2.04	[1.46; 2.62]	14.4%	16.9%
2021_Zheng	25	348.86	31.86	25	246.67	27.87	3.36	[2.48; 4.24]	6.3%	16.5%
**Fixed effect model p < 0.001**	**247**			**247**			**1.55**	**[1.33; 1.77]**	**100.0%**	.
**Random effects model p = 0.001**							**2.80**	**[1.09; 4.50]**	.	**100.0%**

Heterogeneity: $I^2 = 96.8\%$, $\tau^2 = 4.3961$, $p < 0.0001$

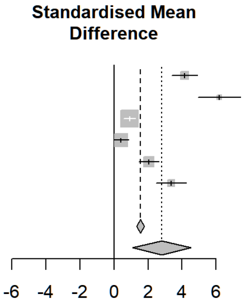

Study	Experimental Total	Experimental Mean	Experimental SD	Control Total	Control Mean	Control SD	MD	95%-CI	Weight (common)	Weight (random)
2018_Yang	20	78.20	10.33	20	70.18	12.47	8.02	[0.92; 15.12]	40.6%	40.6%
2021_Zheng	25	77.42	11.25	25	69.56	9.88	7.86	[1.99; 13.73]	59.4%	59.4%
**Fixed effect model p < 0.001**	**45**			**45**			**7.92**	**[3.40; 12.45]**	**100.0%**	.
**Random effects model p < 0.001**							**7.92**	**[3.40; 12.45]**	.	**100.0%**

Heterogeneity: $I^2 = 0.0\%$, $\tau^2 = 0$, $p = 0.9728$

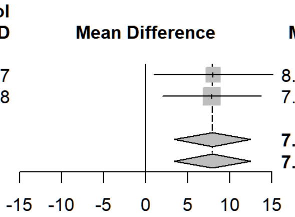

Study	Experimental Total	Experimental Mean	Experimental SD	Control Total	Control Mean	Control SD	MD	95%-CI	Weight (common)	Weight (random)
2006_Li	43	66.35	22.19	42	46.33	18.73	20.02	[11.30; 28.74]	0.4%	11.3%
2008_Song	36	64.36	8.23	36	62.13	7.38	2.23	[-1.38; 5.84]	2.6%	14.2%
2016_Zhang(1)	40	67.45	22.29	40	47.43	18.83	20.02	[10.98; 29.06]	0.4%	11.1%
2016_Zhang(2)	46	86.25	5.68	46	61.85	4.87	24.40	[22.24; 26.56]	7.3%	14.7%
2017_Cui	22	55.91	19.50	22	52.05	20.10	3.86	[-7.84; 15.56]	0.2%	9.4%
2019_Han	30	68.00	3.26	30	52.97	6.80	15.03	[12.33; 17.73]	4.7%	14.6%
2020_Zhao(2)	69	81.96	1.75	69	73.16	2.05	8.80	[8.16; 9.44]	84.1%	15.0%
2021_Zheng	25	66.48	23.68	25	47.26	17.58	19.22	[7.66; 30.78]	0.3%	9.5%
**Fixed effect model p < 0.001**	**311**			**310**			**10.17**	**[9.58; 10.75]**	**100.0%**	.
**Random effects model p < 0.001**							**14.12**	**[8.22; 20.01]**	.	**100.0%**

Heterogeneity: $I^2 = 96.9\%$, $\tau^2 = 60.0738$, $p < 0.0001$

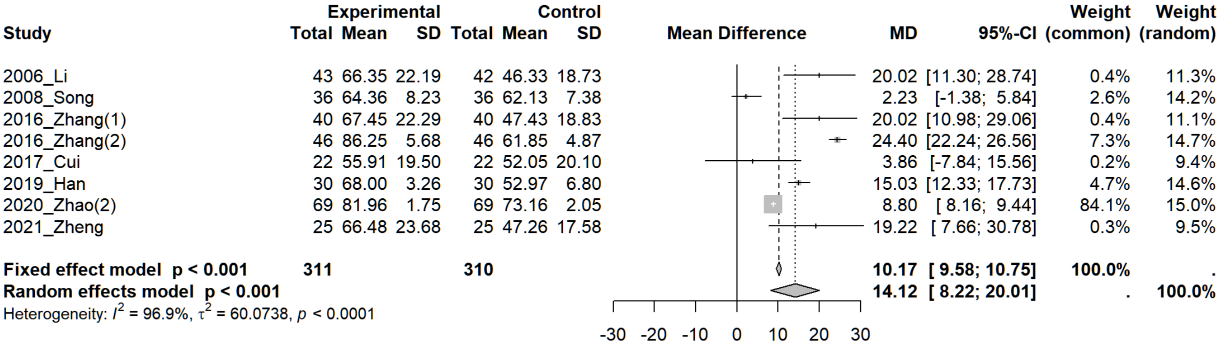

Study	Experimental Total	Experimental Mean	Experimental SD	Control Total	Control Mean	Control SD	MD	95%-CI	Weight (common)	Weight (random)
2008_Song	36	28.36	2.57	36	27.31	5.88	1.05	[-1.05; 3.15]	5.1%	43.0%
2020_Zhao(2)	69	29.14	0.78	69	25.30	1.90	3.84	[3.36; 4.32]	94.9%	57.0%
**Fixed effect model p < 0.001**	**105**			**105**			**3.70**	**[3.23; 4.17]**	**100.0%**	.
**Random effects model p = 0.056**							**2.64**	**[-0.07; 5.35]**	.	**100.0%**

Heterogeneity: $I^2 = 84.5\%$, $\tau^2 = 3.2895$, $p = 0.0110$

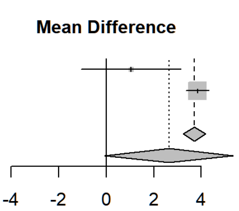

Study	Experimental Total	Experimental Mean	Experimental SD	Control Total	Control Mean	Control SD	MD	95%-CI	Weight (common)	Weight (random)
2017_Cui	22	4.55	1.60	22	5.14	1.64	-0.59	[-1.55; 0.37]	37.1%	33.7%
2019_Wang	40	5.60	1.20	40	12.50	2.40	-6.90	[-7.73; -6.07]	49.2%	33.9%
2020_Zhao(1)	35	8.63	3.26	35	12.12	3.45	-3.49	[-5.06; -1.92]	13.7%	32.4%
**Fixed effect model p < 0.001**	**97**			**97**			**-4.09**	**[-4.67; -3.51]**	**100.0%**	.
**Random effects model p = 0.047**							**-3.67**	**[-7.29; -0.04]**	.	**100.0%**

Heterogeneity: $I^2 = 97.9\%$, $\tau^2 = 9.9090$, $p < 0.0001$

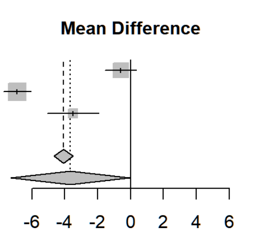

Study	Experimental Total	Experimental Mean	Experimental SD	Control Total	Control Mean	Control SD	MD	95%-CI	Weight (common)	Weight (random)
2018_Yang	20	4.78	1.26	20	8.14	1.37	-3.36	[-4.18; -2.54]	29.9%	31.7%
2020_Zhao(1)	35	7.31	1.68	35	9.54	1.79	-2.23	[-3.04; -1.42]	30.1%	31.8%
2021_Zheng	25	8.12	1.04	25	10.65	1.47	-2.53	[-3.24; -1.82]	40.0%	36.5%
**Fixed effect model p < 0.001**	**80**			**80**			**-2.69**	**[-3.13; -2.24]**	**100.0%**	.
**Random effects model p < 0.001**							**-2.70**	**[-3.33; -2.06]**	.	**100.0%**

Heterogeneity: $I^2 = 50.2\%$, $\tau^2 = 0.1585$, $p = 0.1341$

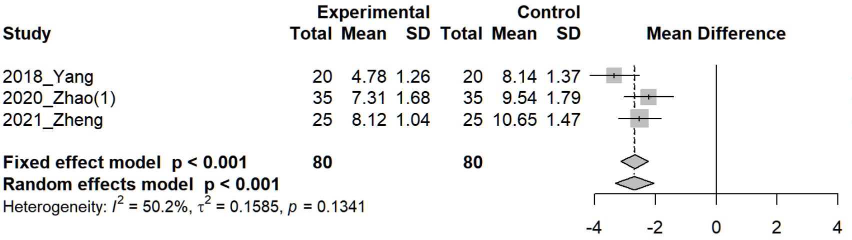

Study	Experimental Total	Experimental Mean	Experimental SD	Control Total	Control Mean	Control SD	MD	95%-CI	Weight (common)	Weight (random)
2006_Li	43	5.13	0.97	42	12.77	2.52	-7.64	[-8.46; -6.82]	28.7%	14.7%
2008_Song	36	21.89	1.57	36	20.78	4.06	1.11	[-0.31; 2.53]	9.5%	14.3%
2009_Zou	30	14.91	3.94	30	18.52	4.01	-3.61	[-5.62; -1.60]	4.7%	13.7%
2011_Dang	52	6.53	3.43	50	9.26	3.82	-2.73	[-4.14; -1.32]	9.6%	14.3%
2013_Zou	40	13.88	3.75	40	18.64	3.96	-4.76	[-6.45; -3.07]	6.7%	14.0%
2016_Zhang(1)	40	5.23	0.97	40	13.87	2.62	-8.64	[-9.51; -7.77]	25.5%	14.7%
2021_Zheng	25	5.57	0.75	25	13.24	2.75	-7.67	[-8.79; -6.55]	15.3%	14.5%
**Fixed effect model p < 0.001**	**266**			**263**			**-6.22**	**[-6.66; -5.78]**	**100.0%**	**.**
**Random effects model p < 0.001**							**-4.89**	**[-7.47; -2.31]**	**.**	**100.0%**

Heterogeneity: I^2 = 96.7%, τ^2 = 11.6245, p < 0.0001

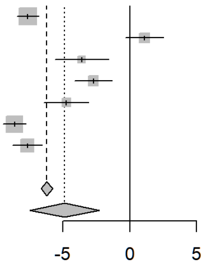

Supplementary Figure 3. Meta regression analysis

3.1 HAMD meta regression : by days

3.2 TER(RR) meta regression : by days

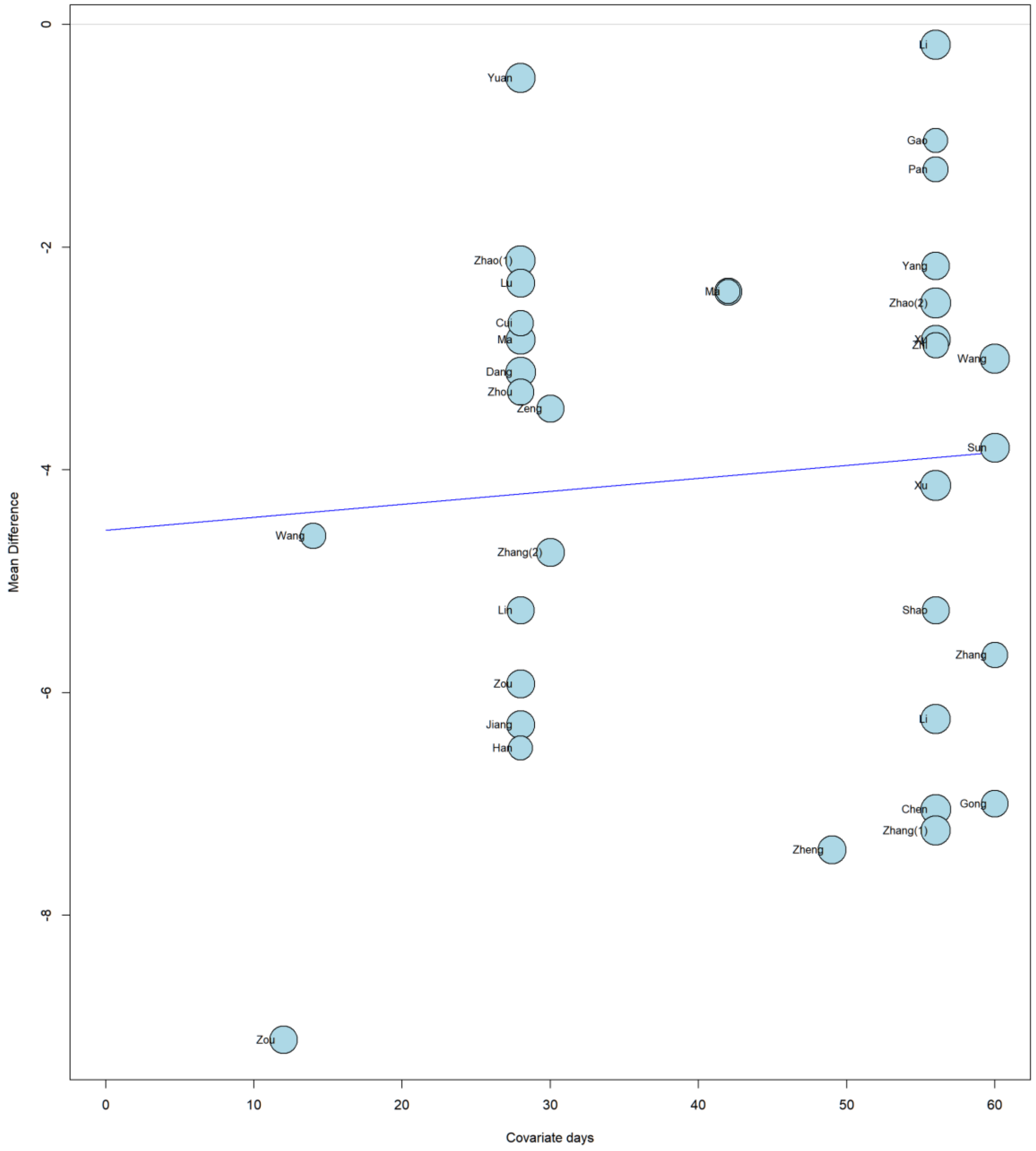

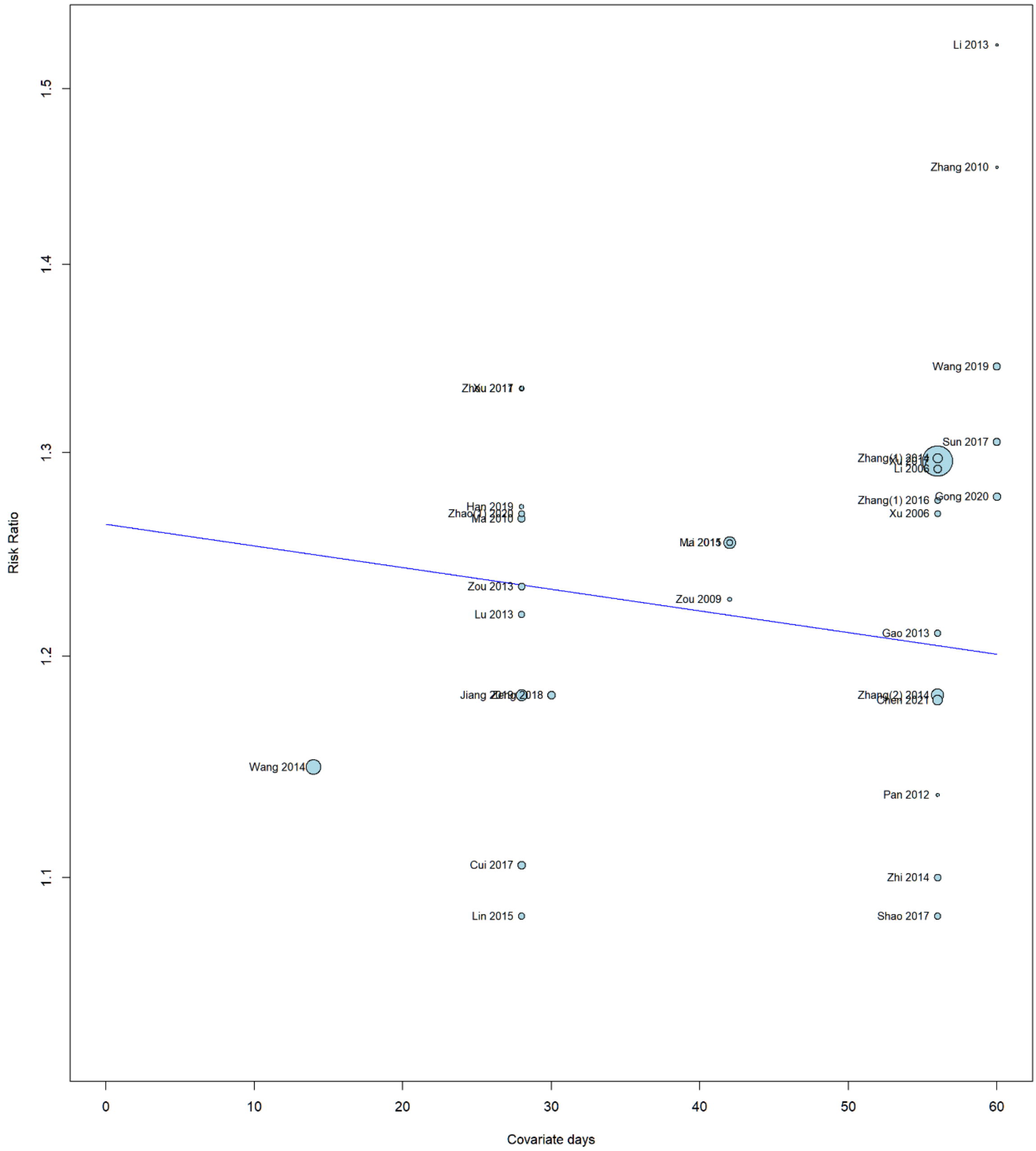

Risk Ratio
Covariate days
1.1
1.2
1.3
1.4
1.5
0
10
20
30
40
50
60
Li 2013
Zhang 2010
Wang 2019
Sun 2017
Gong 2020
Zhang(1) 2016
Xu 2006
Li 2006
Ma 2015
Zou 2009
Gao 2013
Zou 2013
Lu 2013
Han 2019
Ma 2010
Zhang(2) 2014
Chen 2021
Wang 2014
Pan 2012
Cui 2017
Zhi 2014
Lin 2015
Shao 2017

Supplementary Figure 4. Publication bias : TER(RR)

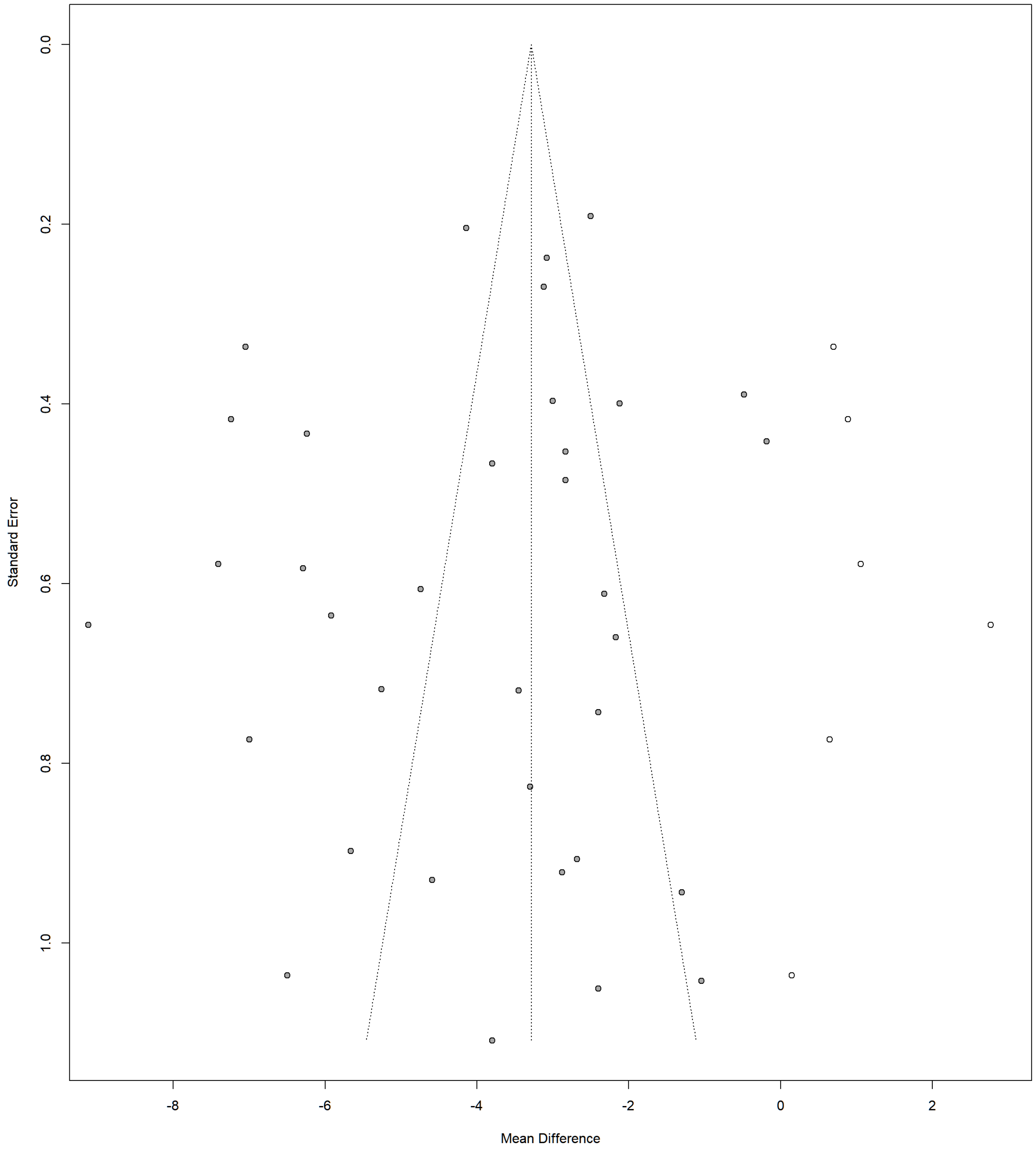

Supplementary Figure 5. Sensitivity analysis : HAMD

5.1 Leave-one-out analysis : Sorted by Effect Size.

5.2 Leave-one-out analysis : Sorted by heterogeneity

5.3 Cumulative meta-analysis : by year

5.4 Cumulative meta-analysis : by number

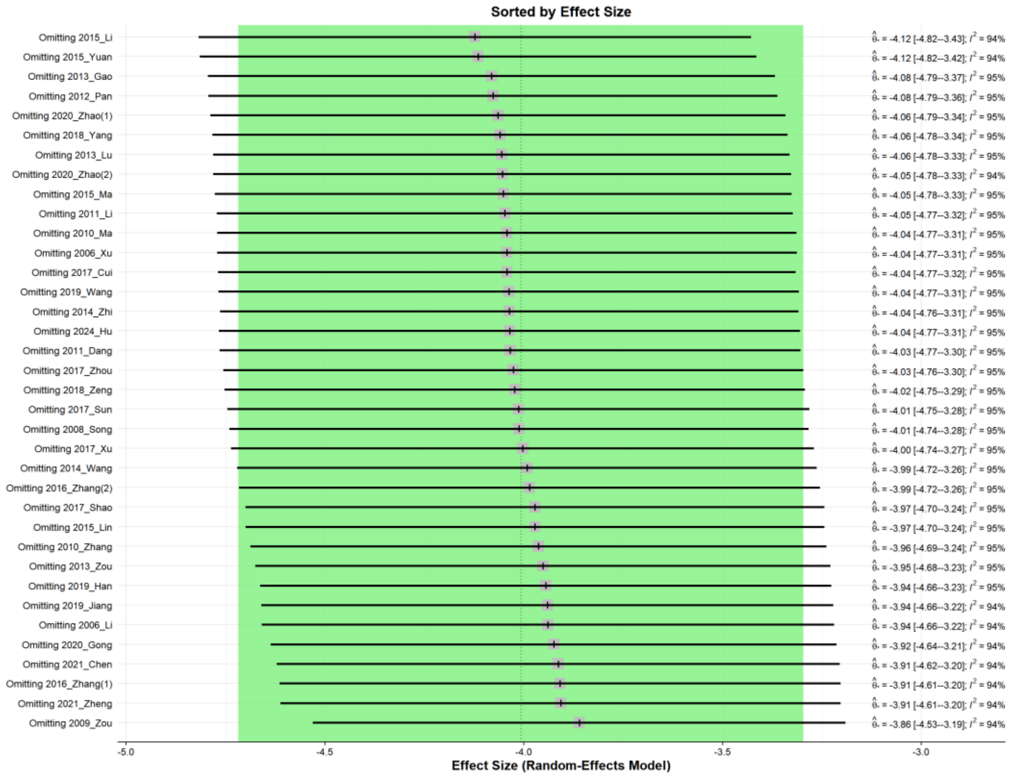

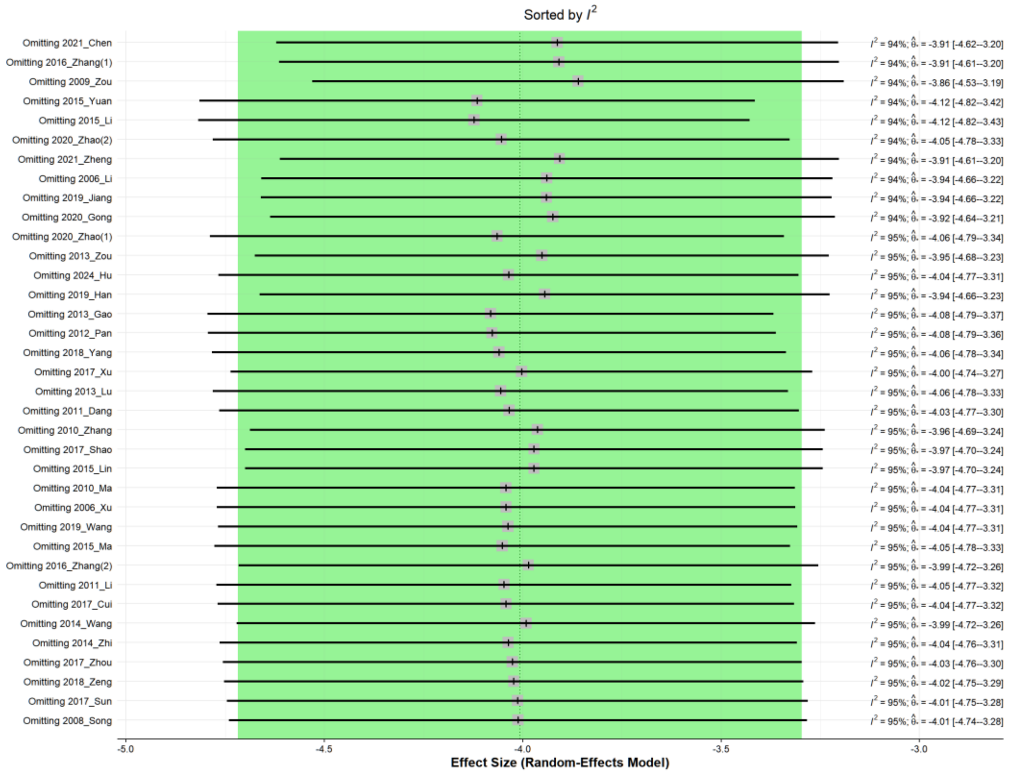

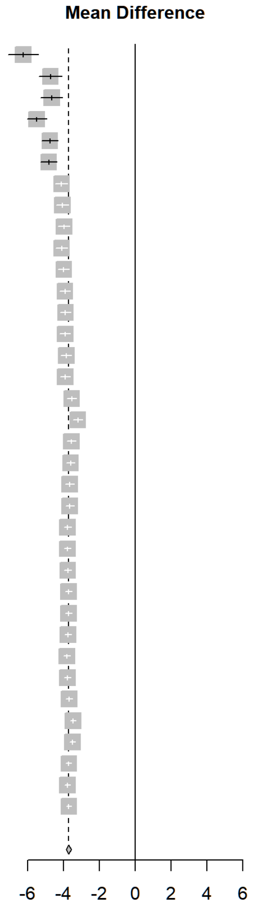

Study	MD	95%-CI	P-value	Tau2	Tau	I2
Adding 2006_Li (k=1)	-6.24	[-7.09; -5.39]	< 0.01	.	.	.
Adding 2006_Xu (k=2)	-4.73	[-5.36; -4.09]	< 0.01	5.6028	2.3670	96%
Adding 2008_Song (k=3)	-4.65	[-5.26; -4.05]	< 0.01	3.1106	1.7637	93%
Adding 2009_Zou (k=4)	-5.49	[-6.04; -4.94]	< 0.01	7.5089	2.7402	96%
Adding 2010_Ma (k=5)	-4.76	[-5.22; -4.29]	< 0.01	7.0346	2.6523	96%
Adding 2010_Zhang (k=6)	-4.82	[-5.27; -4.37]	< 0.01	5.6917	2.3857	95%
Adding 2011_Dang (k=7)	-4.10	[-4.45; -3.76]	< 0.01	5.2375	2.2886	95%
Adding 2011_Li (k=8)	-4.06	[-4.40; -3.72]	< 0.01	5.0754	2.2529	94%
Adding 2012_Pan (k=9)	-3.97	[-4.30; -3.63]	< 0.01	5.4419	2.3328	94%
Adding 2013_Zou (k=10)	-4.10	[-4.42; -3.78]	< 0.01	5.0753	2.2528	93%
Adding 2013_Lu (k=11)	-3.98	[-4.29; -3.67]	< 0.01	4.9107	2.2160	93%
Adding 2013_Gao (k=12)	-3.91	[-4.22; -3.61]	< 0.01	5.1513	2.2696	93%
Adding 2014_Zhi (k=13)	-3.88	[-4.19; -3.58]	< 0.01	4.7831	2.1870	92%
Adding 2014_Wang (k=14)	-3.90	[-4.20; -3.60]	< 0.01	4.4260	2.1038	91%
Adding 2015_Ma (k=15)	-3.84	[-4.13; -3.55]	< 0.01	4.2318	2.0571	91%
Adding 2015_Lin (k=16)	-3.90	[-4.19; -3.61]	< 0.01	4.0386	2.0096	91%
Adding 2015_Li (k=17)	-3.53	[-3.81; -3.26]	< 0.01	4.6692	2.1608	93%
Adding 2015_Yuan (k=18)	-3.19	[-3.45; -2.93]	< 0.01	4.9817	2.2320	94%
Adding 2016_Zhang(1) (k=19)	-3.55	[-3.80; -3.31]	< 0.01	5.4784	2.3406	95%
Adding 2016_Zhang(2) (k=20)	-3.60	[-3.84; -3.36]	< 0.01	5.2232	2.2854	95%
Adding 2017_Shao (k=21)	-3.65	[-3.88; -3.41]	< 0.01	5.0521	2.2477	95%
Adding 2017_Zhou (k=22)	-3.64	[-3.87; -3.41]	< 0.01	4.8163	2.1946	94%
Adding 2017_Xu (k=23)	-3.77	[-3.97; -3.57]	< 0.01	4.5689	2.1375	94%
Adding 2017_Sun (k=24)	-3.77	[-3.97; -3.57]	< 0.01	4.3457	2.0846	94%
Adding 2017_Cui (k=25)	-3.76	[-3.95; -3.56]	0	4.2079	2.0513	94%
Adding 2018_Yang (k=26)	-3.72	[-3.91; -3.53]	0	4.1234	2.0306	94%
Adding 2018_Zeng (k=27)	-3.71	[-3.91; -3.52]	0	3.9568	1.9892	93%
Adding 2019_Han (k=28)	-3.74	[-3.93; -3.55]	0	4.0273	2.0068	93%
Adding 2019_Jiang (k=29)	-3.81	[-4.00; -3.62]	0	4.0892	2.0222	93%
Adding 2019_Wang (k=30)	-3.76	[-3.95; -3.58]	0	3.9535	1.9883	93%
Adding 2020_Zhao(1) (k=31)	-3.68	[-3.86; -3.50]	0	3.9071	1.9766	93%
Adding 2020_Zhao(2) (k=32)	-3.46	[-3.62; -3.30]	0	3.8164	1.9536	93%
Adding 2020_Gong (k=33)	-3.50	[-3.66; -3.34]	0	3.9876	1.9969	93%
Adding 2021_Chen (k=34)	-3.70	[-3.85; -3.54]	0	4.1933	2.0478	94%
Adding 2021_Zheng (k=35)	-3.77	[-3.92; -3.61]	0	4.4099	2.1000	95%
Adding 2024_Hu (k=36)	-3.70	[-3.85; -3.55]	0	4.2923	2.0718	94%
**Common effect model**	**-3.70**	**[-3.85; -3.55]**	**0**	**4.2923**	**2.0718**	**94%**

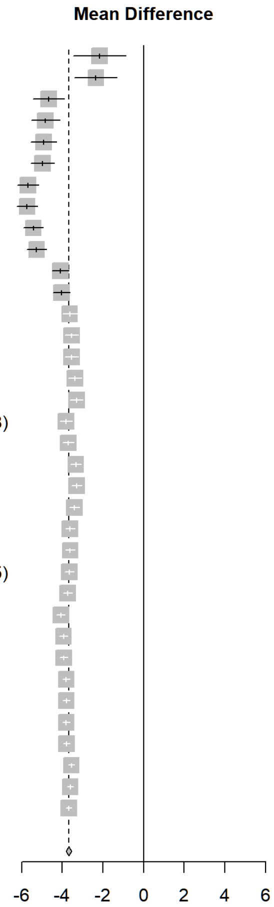

Study	MD	95%-CI	P-value	Tau2	Tau	I2
Adding 2018_Yang (k=1)	-2.17	[-3.46; -0.88]	< 0.01	.	.	.
Adding 2017_Cui (k=2)	-2.35	[-3.39; -1.30]	< 0.01	0	0	0%
Adding 2021_Zheng (k=3)	-4.67	[-5.44; -3.91]	< 0.01	8.0491	2.8371	95%
Adding 2010_Zhang (k=4)	-4.83	[-5.54; -4.13]	< 0.01	5.8247	2.4134	93%
Adding 2015_Lin (k=5)	-4.92	[-5.55; -4.29]	< 0.01	4.3668	2.0897	91%
Adding 2017_Shao (k=6)	-4.97	[-5.55; -4.40]	< 0.01	3.4587	1.8598	88%
Adding 2009_Zou (k=7)	-5.68	[-6.21; -5.16]	< 0.01	5.5330	2.3522	92%
Adding 2019_Han (k=8)	-5.73	[-6.24; -5.23]	< 0.01	4.8590	2.2043	91%
Adding 2012_Pan (k=9)	-5.42	[-5.91; -4.94]	< 0.01	6.0266	2.4549	92%
Adding 2011_Li (k=10)	-5.26	[-5.74; -4.79]	< 0.01	5.9762	2.4446	92%
Adding 2015_Li (k=11)	-4.09	[-4.50; -3.67]	< 0.01	7.4550	2.7304	95%
Adding 2017_Zhou (k=12)	-4.04	[-4.44; -3.63]	< 0.01	6.8412	2.6156	95%
Adding 2020_Zhao(1) (k=13)	-3.63	[-3.99; -3.28]	< 0.01	6.5973	2.5685	95%
Adding 2006_Xu (k=14)	-3.53	[-3.87; -3.20]	< 0.01	6.1595	2.4818	94%
Adding 2008_Song (k=15)	-3.54	[-3.87; -3.21]	< 0.01	5.7346	2.3947	94%
Adding 2008_Wang (k=16)	-3.37	[-3.70; -3.05]	< 0.01	8.6213	2.9362	94%
Adding 2013_Lu (k=17)	-3.30	[-3.62; -2.98]	< 0.01	8.1128	2.8483	94%
Adding 2016_Zhang(1) (k=18)	-3.81	[-4.11; -3.52]	< 0.01	8.4430	2.9057	95%
Adding 2010_Ma (k=19)	-3.72	[-4.00; -3.44]	< 0.01	7.9557	2.8206	95%
Adding 2015_Yuan (k=20)	-3.33	[-3.59; -3.07]	< 0.01	8.0436	2.8361	95%
Adding 2019_Wang (k=21)	-3.30	[-3.55; -3.05]	< 0.01	7.5990	2.7566	95%
Adding 2013_Zou (k=22)	-3.40	[-3.64; -3.15]	< 0.01	7.4860	2.7361	95%
Adding 2006_Li (k=23)	-3.62	[-3.85; -3.38]	< 0.01	7.4397	2.7276	95%
Adding 2018_Zeng (k=24)	-3.61	[-3.84; -3.38]	< 0.01	7.0891	2.6625	95%
Adding 2016_Zhang(2) (k=25)	-3.65	[-3.88; -3.43]	< 0.01	6.8052	2.6087	95%
Adding 2020_Gong (k=26)	-3.73	[-3.95; -3.50]	< 0.01	6.9177	2.6301	95%
Adding 2021_Chen (k=27)	-4.07	[-4.28; -3.86]	0	7.0369	2.6527	96%
Adding 2011_Dang (k=28)	-3.94	[-4.14; -3.74]	0	6.7666	2.6013	95%
Adding 2017_Sun (k=29)	-3.93	[-4.13; -3.74]	0	6.4923	2.5480	95%
Adding 2024_Hu (k=30)	-3.81	[-3.99; -3.63]	0	6.2598	2.5020	95%
Adding 2014_Zhi (k=31)	-3.80	[-3.98; -3.62]	0	6.0734	2.4644	95%
Adding 2014_Wang (k=32)	-3.81	[-3.98; -3.63]	0	5.8819	2.4253	95%
Adding 2015_Ma (k=33)	-3.79	[-3.96; -3.61]	0	5.7499	2.3979	95%
Adding 2020_Zhao(2) (k=34)	-3.56	[-3.71; -3.40]	0	5.6075	2.3680	95%
Adding 2019_Jiang (k=35)	-3.61	[-3.76; -3.45]	0	5.6059	2.3677	95%
Adding 2017_Xu (k=36)	-3.68	[-3.82; -3.53]	0	5.4166	2.3274	95%
**Common effect model**	**-3.68**	**[-3.82; -3.53]**	**0**	**5.4166**	**2.3274**	**95%**
